# Iron-Sulfur (Fe/S) Protein Biogenesis: Phylogenomic and Genetic Studies of A-Type Carriers

**DOI:** 10.1371/journal.pgen.1000497

**Published:** 2009-05-29

**Authors:** Daniel Vinella, Céline Brochier-Armanet, Laurent Loiseau, Emmanuel Talla, Frédéric Barras

**Affiliations:** 1 Laboratoire de Chimie Bactérienne, Institut Fédératif de Recherche 88 - Institut de Microbiologie de la Méditerranée, Centre National de la Recherche Scientifique, Marseille, France; 2 Aix-Marseille Université, Marseille, France; Stanford University, United States of America

## Abstract

Iron sulfur (Fe/S) proteins are ubiquitous and participate in multiple biological processes, from photosynthesis to DNA repair. Iron and sulfur are highly reactive chemical species, and the mechanisms allowing the multiprotein systems ISC and SUF to assist Fe/S cluster formation *in vivo* have attracted considerable attention. Here, A-Type components of these systems (ATCs for A-Type Carriers) are studied by phylogenomic and genetic analyses. ATCs that have emerged in the last common ancestor of bacteria were conserved in most bacteria and were acquired by eukaryotes and few archaea via horizontal gene transfers. Many bacteria contain multiple ATCs, as a result of gene duplication and/or horizontal gene transfer events. Based on evolutionary considerations, we could define three subfamilies: ATC-I, -II and -III. *Escherichia coli*, which has one ATC-I (ErpA) and two ATC-IIs (IscA and SufA), was used as a model to investigate functional redundancy between ATCs *in vivo*. Genetic analyses revealed that, under aerobiosis, *E. coli* IscA and SufA are functionally redundant carriers, as both are potentially able to receive an Fe/S cluster from IscU or the SufBCD complex and transfer it to ErpA. In contrast, under anaerobiosis, redundancy occurs between ErpA and IscA, which are both potentially able to receive Fe/S clusters from IscU and transfer them to an apotarget. Our combined phylogenomic and genetic study indicates that ATCs play a crucial role in conveying ready-made Fe/S clusters from components of the biogenesis systems to apotargets. We propose a model wherein the conserved biochemical function of ATCs provides multiple paths for supplying Fe/S clusters to apotargets. This model predicts the occurrence of a dynamic network, the structure and composition of which vary with the growth conditions. As an illustration, we depict three ways for a given protein to be matured, which appears to be dependent on the demand for Fe/S biogenesis.

## Author Summary

Iron sulfur (Fe/S) proteins are found in all living organisms where they participate in a wide array of biological processes. Accordingly, genetic defects in Fe/S biogenesis yield pleiotropic phenotypes in bacteria and several syndromes in humans. Multiprotein systems that assist Fe/S cluster formation and insertion into apoproteins have been identified. Most systems include so-called A-type proteins (which we refer to as ATC proteins hereafter), which have an undefined role in Fe/S biogenesis. Phylogenomic analyses presented, here, reveal that the ATC gene is ancient, that it was already present in the last common ancestor of bacteria, and that it subsequently spread to eukaryotes via mitochondria or chloroplastic endosymbioses and to a few archaea via horizontal gene transfers. Proteobacteria are unusual in having multiple ATCs. We show by a genetic approach that the three ATC proteins of *E. coli* are potentially interchangeable, but that redundancy is limited *in vivo*, either because of gene expression control or because of inefficient Fe/S transfers between ATCs and other components within the Fe/S biogenesis pathway. The combined phylogenomic and genetic approaches allow us to propose that multiple ATCs enable *E. coli* to diversify the ways for conveying ready-made Fe/S clusters from components of the biogenesis systems to apotargets, and that environmental conditions influence which pathway is used.

## Introduction

Fe/S proteins are present in all living cells, where they participate in a wide range of physiological processes including respiration, photosynthesis, DNA repair, metabolism and regulation of gene expression [1],[2]. Since the discovery of a dedicated system for the maturation of nitrogenase in *Azotobacter vinelandii* [3],[4], the *in vivo* mechanism of Fe/S cluster formation and insertion into proteins has received increasing attention. A main conclusion is that this process involves several protein factors that are conserved throughout eukaryotes and prokaryotes [5]–[11].

Besides the NIF system dedicated to nitrogenase maturation, general systems involved in Fe/S cluster formation, called ISC and SUF, have been described. Sulfur is provided to these systems by cysteine desulfurases, referred to as NifS, IscS, and SufS [3], [12]–[16]. The role of CsdA, another *E. coli* cysteine desulfurase, remains to be assessed [17],[18]. In eukaryotes, iron is provided by frataxin [19], and its alteration causes pathological disorders, *e.g.* Friedreich ataxia in humans [20],[21]. In prokaryotes, frataxin-like, ferritin-like proteins and siderophores were proposed to provide iron for Fe/S biogenesis [22]–[25]. Scaffold proteins bind iron and sulfur and form Fe/S clusters that are eventually transferred to apotargets. Scaffold proteins were first described in the nitrogen fixing bacterium *A. vinelandii* [26], and subsequently in other bacteria, yeast, plants and humans, where they are referred to as IscU, Isu, NifU or Nfu [5]–[8], [27]–[29]. The SUF and ISC systems also include ATP-hydrolyzing proteins such as SufBCD, a pseudo ABC ATPase complex [24],[30], or HscBA, a DnaJK-like co/chaperone system [31],[32]. Recent results from the Fontecave lab showed that SufB contains an Fe/S cluster and forms a complex with SufC and SufD that can transfer this Fe/S cluster to apotargets ([30], M. Fontecave *et al.*, personal communication). These observations lead to the hypothesis that the SufBCD complex could fulfill the scaffold function within the SUF system. Last, there are the A-type proteins, *e.g.* IscA, SufA, IscA^Nif^, ErpA, ISA1 and ISA2, whose biochemical functions and cellular roles remain unclear despite their presence in most studied systems. They are the objects of the present study, where they will be referred to as A-Type Carrier (ATC).

An important issue regarding the role of ATCs pertains to their specificity *in vivo*. Indeed, genomes of model organisms, such as *E. coli* or *S. cerevisiae*, encode several ATC homologs, raising the question of whether they are functionally redundant. For instance, *E. coli* synthesizes three ATCs, IscA, SufA and ErpA, sharing 30% sequence identity [33]. Whereas the inactivation of *iscA* or *sufA* was almost neutral, an *erpA* mutation was found to be lethal under respiratory growth conditions [33]. Also, work on *A. vinelandii* revealed that an *iscA* mutation was lethal at high oxygen concentration [34]. Hence, these studies supported the notion that there are functional differences between the ATCs *in vivo*. On the other hand, functional redundancy between ATCs was indicated by other experiments: (i) in *E. coli*, an *iscA sufA* double mutant was found to exhibit a strongly decreased growth rate whereas single mutants grew almost like a wild type strain [35],[36, see Discussion], (ii) in *S. cerevisiae*, a double *ISA1 ISA2* knockout exhibited a series of mitochondrial phenotypes not shown by single mutants [37]–[39]. Importantly, the perception that ATCs are functionally redundant is consistent with *in vitro* studies where either of the ATCs tested was found to transfer Fe/S to apotargets with similar efficiency [7],[29].

In this work, we combined phylogenomic and genetic approaches to answer several of the questions raised by the presence of an ATC in each Fe/S biogenesis system studied so far. Phylogenomic studies allowed us to trace the emergence of ATCs in bacteria and to follow their evolution throughout living organisms. This analysis gave insight on the question of when and where genetic redundancy arose and allowed the identification of three subfamilies (-I, -II and -III) of ATC. In this regard, Gamma-Proteobacteria, including *E. coli*, stood out as being exceptional in containing three ATCs belonging to ATC-I and ATC-II subfamilies. The genetic study showed that these ATCs are potentially interchangeable but that this bacterium has evolved to prevent full redundancy by controlling the synthesis of each ATC and the efficiency of its partnership with other components of the Fe/S biogenesis line. Phylogenomic and genetic data indicate that the evolution of ATC-II members was constrained by their partnership with scaffolds from which they receive Fe/S clusters, whereas the evolution of ATC-I members was mainly driven by their partnership with the apotargets to which they transfer Fe/S clusters. The net result is that the cell exploits the multiple ATCs homologs by positioning them between the cysteine desulfurase/scaffold duo and the apotargets, where they act as connectors between different routes for conveying Fe/S clusters from the scaffold to apotargets.

## Results

### Distribution of the ATC Proteins

**Figure 1:**
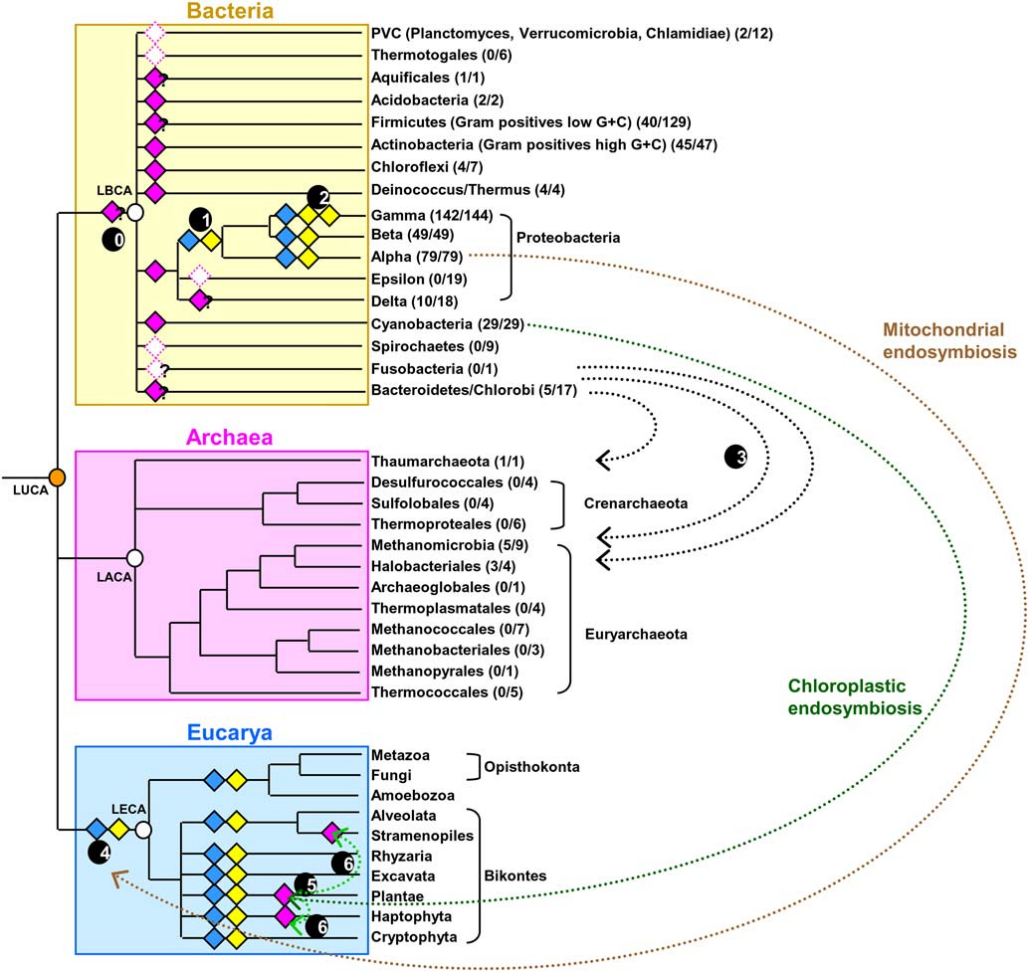
Model for ATC protein evolution. Schematic representation of the universal tree of life, for which complete genome sequences are available. LUCA (Last Universal Common Ancestor), LECA (Last Eukaryotic Common Ancestor), LACA (Last Archaeal Common Ancestor) and LBCA (Last Bacterial Common Ancestor) are indicated by orange and white circles. For each prokaryotic phylum, the number of genomes encoding at least one ATC homolog with respect to the number of complete available genomes is given between brackets. Filled-diamonds indicate the presence of an ATC encoding gene in the ancestor of a given lineage: pink diamonds designate ATCs; blue diamonds symbolize ATC-I and yellow diamonds represent ATC-II. Dotted empty diamonds symbolize the loss of the corresponding ATC ancestor encoding gene in the lineage. Arrows schematize horizontal gene transfer events (HGT). The distribution and phylogeny of ATC proteins suggest that they originated in the bacterial domain (black circle number 0) and were thus absent in LACA. The inference of ATC encoding genes in the ancestor of most bacterial phyla (*i.e.* Acidobacteria, Chloroflexi, Actinobacteria, Cyanobacteria, Deinococcus/Thermus, Alpha-, Beta and Gamma-Proteobacteria) suggests that an ATC encoding gene was present in LBCA. Accordingly, the absence of any ATC encoding gene in PVC, Thermotogae, Epsilon-Proteobacteria, and Spirochaetes suggests ancestral losses whereas the ancestral presence or absence of ATC encoding genes in Bacteroidetes/Chlorobi, Delta-Proteobacteria, Aquificae, Fusobacteria and Firmicutes cannot be definitively inferred. In nearly all Alpha-, Beta- and Gamma-Proteobacteria, at least two ATC encoding genes are present, suggesting their presence in the ancestor of these lineages (blue and yellow filled-diamonds). The evolutionary event at the origin of these two copies cannot be definitively inferred (acquisition through HGT of a non-proteobacterial bacterial sequence or duplication of the native copy, black circle number 1). The acquisition of a third copy in Gamma-Proteobacteria through a duplication event or an HGT occurred later (black circle number 2). The presence of few ATC encoding genes in Archaea likely results from several independent HGTs from different bacterial donors (black arrows and black circle number 3). The two ATC encoding genes found in nearly all eukaryotes are orthologs to the two copies found in Alpha-Proteobacteria and were very likely acquired through the mitochondrial endosymbiosis by their last common ancestor (brown arrow, black circle number 4). The third ATC encoding gene found in Plantae was likely acquired through the primary chloroplastic endosymbiosis (dark green arrow and black circle number 5) and spread to other photosynthetic eukaryotes through secondary chloroplastic endosymbioses (black circles number 6).

Experimentally characterized ATCs (*e.g.* ErpA, SufA, IscA, IscA^Nif^, ISA1 and ISA2) contain a single domain of ∼100 residues referred to as PF01521 in the Pfam database. By using this domain as a query, we detected 991 proteins within 622 complete prokaryotic genomes. Among these proteins, 192 contained a truncated PF01521 C-terminal domain, *e.g.* in Firmicutes, or additional domains, *e.g.* NfuA proteins found in Gamma-Proteobacteria that contained an Nfu domain fused to PF01521 (see “other” category in Table S1). Similar to the well-characterized ATCs, 799 proteins harbored a single complete PF01521 domain, and were thus considered ATC proteins *sensu stricto*. These 799 ATC proteins were present in a majority of prokaryotes (436 of 622, ∼70%, Table S1 and Figure 1). In contrast, ATC proteins are absent in all Epsilon-Proteobacteria, Spirochaetes, Fusobacteria, Thermotogae and nearly all Archaea and PVC (Planctomycetales-Verrucomicrobia-Chlamydiae) as well as in many Firmicutes, Delta-Proteobacteria and Bacteroidetes/Chlorobi (Table S1 and Figure 1). Except in the amitochondria *Entamoeba* and the highly divergent amitochondriate Microsporidia, ATC encoding genes have been detected in all eukaryotic genomes. More precisely, amitochondriate anaerobic eukaryotes such as *Giardia lamblia* or *Trichomonas vaginalis* were found to possess only one ATC encoding gene whereas all other eukaryotes encoded at least two ATCs. A third copy is present in the genome of photosynthetic eukaryotes (*e.g.* Viridiplantae, Rhodophyta and Stramenopiles, see Table S1). The high conservation of ATC highlights the functional importance of these proteins in eukaryotes and bacteria.

### Phylogenomic Analysis of the ATC Proteins

In order to study the origin and the evolution of the ATC genes, we performed a phylogenomic analysis of the ATC proteins. We inferred the presence of an ATC in the ancestor of a phylum if, in the ATC phylogeny, we found a monophyletic group of ATC that corresponded to the phylum and if the corresponding genes were well distributed within that phylum. The exhaustive phylogenomic analysis of the 911 ATCs from complete prokaryotic genomes showed that the bacterial and the few archaeal sequences did not form two distinct clusters (Figure S1). In fact, archaeal homologs emerged from within different bacterial subgroups. For instance, sequences from *Methanosarcina* are close to Firmicutes whereas those from Halobacteriales and Delta-Proteobacteria group together (Figure S1). This result, combined with the scarcity of ATC sequences in archaea strongly suggested that the Last Archaeal Common Ancestor (LACA) had no ATC and that a few archaea independently acquired their ATC through horizontal gene transfer (HGT) from bacteria (Figure 1, black circle number 3).

**Figure 2:**
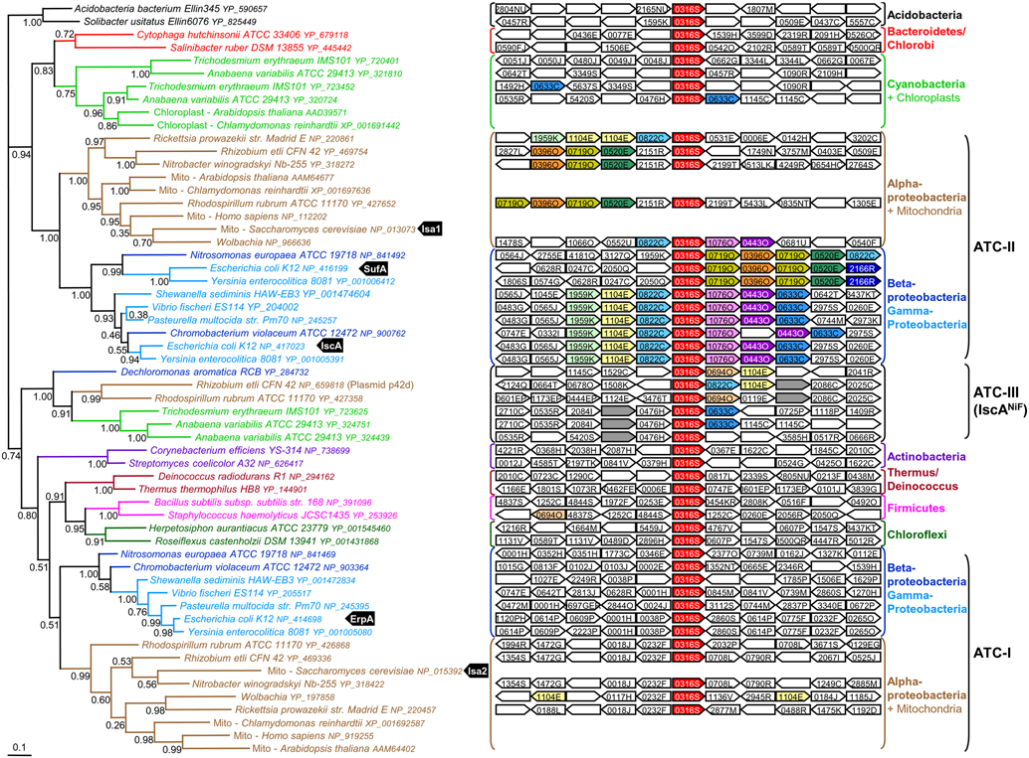
Phylogeny of the ATC proteins. Left part: Bayesian tree of a sample of 58 ATC sequences representative of the ATC family diversity. Numbers at nodes represent posterior probabilities. The scale bar represents the average number of substitutions per site. Right part: for each bacterial homolog, the genomic organization of regions surrounding ATC coding genes (ATC, COG0316S, red boxes) is shown. Numbers in boxes correspond to COG (Cluster of Othologous Groups) numbers. Colored boxes represent genes coding for components of the ISC system [HscB (COG1076O, pink boxes); HscA (COG0443O, purple boxes); Fdx (COG0633C, blue boxes); IscU (COG0822C, light blue boxes); IscS (COG1104E, yellow boxes) and IscR (COG1959K, light green boxes)], the SUF system [SufB/D (COG0719O, dark yellow boxes); SufS (COG0520E, dark green); SufC (COG0396O, orange boxes) and SufE (COG2166R, dark blue boxes)] or the NIF sytem [NifW (dark grey), NifU (COG0694O, light orange), NifS (COG1104E, yellow boxes)].

Next, in order to pinpoint the origin of bacterial and eukaryotic ATCs, we selected a subset of sequences representative of their diversity to perform a more in-depth phylogenomic analysis. The overall topology of the resulting Bayesian tree was in agreement with the exhaustive phylogeny of the ATC family (Figures 2 and S1). This tree showed a number of monophyletic groups, most of them well supported, which corresponded to major bacterial phyla (Figure 2), *i.e.* Actinobacteria (Posterior Probability = 1.0), Acidobacteria (PP = 1.00), Chloroflexi (PP = 0.91), Cyanobacteria (PP = 0.75), Deinococcus/Thermus (PP = 1.0), and two clusters of Alpha-, Beta- and Gamma-Proteobacteria (PP = 1.0 and PP = 0.51). The wide distribution of the ATC encoding genes in these phyla suggested the presence of such a gene in their ancestors (Figure 1, pink filled-in diamonds and Figure S1). In contrast, ATC proteins were probably absent in the ancestors of the PVC, Spirochaetes, Thermotogae and Epsilon-Proteobacteria (Figure 1, pink empty diamonds). The situation was less clear for the remaining bacterial groups (Figure 1, diamonds with “?” inside), in which ATCs did not form monophyletic groups (*e.g.* Delta-Proteobacteria and Bacteroidetes/Chlorobi, Figure S1) or are too scarce, (*e.g.* Firmicutes, Table S1) or because too few representatives of the phylum were available (*i.e.* only one representative for Aquificae and for Fusobacteria, Figure 1). The presence of an ATC-encoding gene in the ancestors of a number of bacterial phyla indicated that the corresponding protein is ancient in Bacteria and may already have been present in the last ancestor of this domain (Last Bacterial Common Ancestor, Figure 1, black circle number 0). Consequently, the absence of any ATC-encoding gene in some bacterial lineages likely reflects secondary losses (empty pink diamonds in Figure 1). Caution should be taken to not extrapolate these observations to other components of the SUF and ISC systems as their own history will require a thorough phylogenomic analysis.

Eukaryotic ATC sequences form three distinct groups in the phylogeny. Two of these emerged within each of the two Alpha-, Beta- and Gamma-Proteobacteria groups (PP = 1.0 and PP = 0.99), close to Alpha-Proteobacterial sequences (Figure 2). This did not support the hypothesis that genome duplication was responsible for the appearance of the two eucaryotic ATC copies but rather indicated that the Last Eukaryotic Common Ancestor (LECA) acquired two ATC from these Proteobacteria, likely through endosymbiosis (Figure 1, black circle number 4). The third group of eukaryotic sequences corresponded to the additional copy that is present only in photosynthetic eukaryotes and groups with cyanobacterial sequences (PP = 0.96), confirming its acquisition through the primary chloroplastic endosymbiosis in the ancestor of Plantae (Figure 1, black circle number 5). The presence of this gene in photosynthetic protists such as Stramenopiles indicated that it has been conserved through secondary chloroplastic endosymbioses (Figure 1, black circle number 6).

### Defining Three Classes of ATC Proteins

Our phylogenomic analysis showed that ATC likely originated in the Last Bacterial Common Ancestor and were conserved in most bacterial lineages (Figure 1). Surprisingly, the genomes of nearly all Alpha-, Beta- and Gamma-Proteobacteria were found to code for at least two ATCs *sensu stricto* (Table S1). The phylogenomic analysis of ATCs showed that these sequences formed two distinct monophyletic clusters (PP = 1.0 and PP = 0.51, Figure 2). In agreement with the phylogeny of proteobacteria within each cluster, Alpha-, Beta- plus Gamma-Proteobacteria each formed a monophyletic group. These observations indicated that the common ancestor of these Proteobacteria already contained two distinct ATCs that we propose to call ATC-I and ATC-II (Figure 1, blue and yellow filled-in diamonds). ATC-I includes ErpA from *E. coli* as well as yeast ISA2 whereas ATC-II includes both the SufA and IscA proteins from *E. coli* and yeast ISA1. ATC-I and ATC-II were conserved during the evolution of these Proteobacteria and in nearly all eukaryotes after their acquisition through the mitochondrial endosymbiosis. The poor resolution of the ATC phylogeny, in particular for the most basal nodes due to the small size of the set of sequences, did not allow us to identify the evolutionary event at the origin of ATC-I and ATC-II in Proteobacteria (Figure 1, black circle number 1). Two hypotheses can be proposed: (i) one of these two ATCs was acquired by the ancestor of Alpha-, Beta- and Gamma-Proteobacteria *via* a HGT of an ATC gene from a non-proteobacterial donor, or (ii) these two ATCs arose from duplication of the native proteobacterial ATC gene. Few Gamma-Proteobacteria, mainly Enterobacteriales, harbor two ATC-II homologs, suggesting that these two copies emerged recently in this group, possibly through the duplication of an ancestral ATC-II copy (Figure 1, black circle number 2). This led us to divide the Gamma-proteobacterial ATC-II in two groups: ATC-IIa that includes *E. coli* IscA and ATC-IIb that contains *E. coli* SufA. Interestingly, the comparison of evolutionary distances within the ATC-I, IIa and IIb subfamilies reveals that the corresponding ATC sequences did not evolve at the same rate. This is highlighted by the fact that the lengths of the branches separating ATC sequences from two organisms are different according to the considered family. For example, the evolutionary distances deduced from the tree of Figure 2 between ATC sequences from *E. coli* K12 and its close relative *Yersinia enterolitica* 8081 are 0.32 substitutions per site for ATC-IIb, 0.163 for ATC-IIa and 0.068 for ATC-I. This clearly indicated that ATC-IIb sequences are the fastest evolving sequences whereas ATC-I sequences are the slowest.

Finally, the ATC phylogeny showed another well supported subfamily of ATCs that includes sequences annotated as IscA^Nif^ involved in nitrogenase maturation. We propose classifying them as a third ATC subfamily (ATC-III, PP = 1.0, Figure 2). These sequences are present in organisms from various bacterial phyla (Supplementary Table S1), suggesting that they spread to various bacteria via HGT. Based on our data, it is not possible to determine in which bacterial phylum this third ATC family originated.

### Relationships between ATC Encoding Genes and Their Genetic Contexts

The analysis of the genomic context of ATC genes led us to several new considerations. We first observed that the genetic context of non-proteobacterial ATC and proteobacterial ATC-I encoding genes was not conserved and very rarely contained genes encoding ISC or SUF components (Figure 2). In contrast, most of the ATC-II encoding genes were surrounded by genes encoding ISC or SUF components (Figure 2). More precisely, in Beta- and Gamma-Proteobacteria, most ATC-II encoding genes, including *E. coli* ATC-IIa member *iscA*, lie close to genes coding for the ISC system. In contrast, ATC-IIb genes are associated with genes coding for components of the SUF system as it is the case for *E. coli sufA*. This indicated that the association of ATC-II with genes encoding components of the ISC system is likely ancestral in these two proteobacterial subdivisions. Our phylogenomic analysis of the ATCs suggested that the two ATC-II copies observed in some Gamma-Proteobacteria resulted from a recent evolutionary event. It is possible that one of the two resulting copies (*i.e.* ATC-IIb) was subsequently associated with the SUF system, whereas the other (*i.e.* ATC-IIa) remained associated with the ISC system. The situation is less clear for the single ATC-II from Alpha-Proteobacteria since the type of association seemed to have changed many times during the evolution of this subdivision. For example, ATC-II encoding genes from Rickettsiales (*e.g.* from *Rickettsia* or *Wolbachia*) are associated with genes from the ISC system, whereas their close relatives (*e.g.* from *Rhizobium*, *Nitrobacter* or *Rhodospirilum*) are in the neighborhood of the SUF system encoding genes (Figure 2).

The genes belonging to the ATC-III family are surrounded by genes annotated as *nif* (Figure 2). This strongly suggests that all these ATC-III encoding genes are indeed involved in nitrogenase maturation. Because we showed that ATC-III genes spread by HGT among bacteria from different phyla, this suggests that these HGTs may have involved the whole NIF gene cluster.

### *In vivo* Redundancy versus Specificity: a Genetic Approach in *E. coli*

*E. coli* emerged from the phylogenomic approach as representative of the subset of bacteria that synthesize three ATCs: one type I, ErpA, and two type II, IscA and SufA. We next exploit the genetic amenability of this organism to investigate the redundancy *versus* specificity of the ATC proteins. Results are presented in the sections thereafter.

### *iscA* and *sufA* Mutations Are Synthetically Lethal under Aerobic Conditions

To investigate a potential redundancy between *iscA* and *sufA*, we sought to construct a strain lacking both genes. For this purpose, the Δ*iscA*::*cat* mutation present in strain DV698 was transduced into strain DV701 (Δ*sufA*). The number of tranductants was surprisingly low, two orders of magnitude less, at best, than with the wild type MG1655 used as recipient (Table 1). Similarly, the Δ*sufA*::*kan* mutation could not be P1-transduced into the Δ*iscA* mutant strain DV699. These observations suggested that combining the *iscA* and *sufA* mutations in the same background was lethal.

**Table 1:** *iscA* and *sufA* mutations are synthetically lethal in the presence of oxygen.

	Number of clones on selective plates		Observed co-transduction frequency	
	P1/*sufA*::*kan*	P1/*iscA*::*cat*	P1/*sufA*::*kan zdi-925*::Tn*10*	P1/*iscA*::*cat zfh-208*::Tn*10*
Recipient strains	Selection LB Kan	Selection LB Cam	Selection LB Tet	Selection LB Tet
MG1655	>200	>200	35%+/−5	75%+/−5
Δ*sufA* (DV701)	n.d.	0	n.d.	<1%
Δ*iscA* (DV699)	0	n.d.	<1%	n.d.

All transductions were carried out as described [62] using overnight grown LB culture of the recipient strains. Sodium citrate (10^−2^ M) was added in all the selective media. Clones were counted and/or purified on the same medium after 5 days. Direct transduction (1^st^ and 2^sd^ column): 10^9^ WT and mutant cells were infected by the 10^8^ phages from the same P1 stock. In a control experiment carried out under the same conditions but using a P1 stock made on strain *zdi-92*5::Tn*10*, the WT and the mutants gave similar numbers of Tet^R^ clones (data not shown). Co-transduction experiments (3^rd^ and 4^th^ column): 100 transductants were first selected on plates containing tetracycline, purified and subsequently tested for the co-transduction of the *iscA*::*cat* or the *sufA*::*kan* mutations by streaking onto plates containing both tetracyclin and chloramphenicol or tetracycline and kanamycin, respectively. n.d. stands for not determined.

To test the hypothesis that the *iscA* and *sufA* mutations were synthetically lethal, we used a co-transduction strategy, wherein the acquisition of the mutation to be tested is not used as a direct marker in the selection procedure. Hence, we used strain DV1239 (Δ*iscA*::*cat zfh-208*::Tn*10*) in which a Tn*10* transposon (Tet^R^) was inserted near the Δ*iscA*::*cat* (Cam^R^) mutation. We selected Tet^R^ transductants and co-transduction of the Δ*iscA*::*cat* (Cam^R^) was subsequently analyzed. The co-transduction frequency of the Δ*iscA*::*cat* mutation with *zfh*-*208*::Tn*10* was about 75% in the wild type and <1% in the Δ*sufA* DV701 strain (Table 1). This result, in agreement with other data [36],[40], indicated that the presence of a Δ*sufA* mutation in the recipient strain counterselected the subsequent acquisition of the Δ*iscA*::*cat* mutation. The same conclusion could be drawn from the reciprocal experiment carried out using strain DV1230 (Δ*sufA*::*kan zdi-925*::Tn*10*) with a Tn*10* located in the vicinity of the *sufA* gene as a P1 donor, and DV699 (Δ*iscA*) as recipient (Table 1). We verified that the incompatibility between the *iscA* and *sufA* mutations was not due to polar effects on downstream genes in the *isc* or *suf* operon (*see* Materials and Methods). Taken together, these experiments established that *iscA* and *sufA* null mutations are synthetically lethal under aerobic conditions, suggesting that the IscA and SufA proteins are redundant for some function(s) essential for cell survival.

### An *iscA sufA* Double Mutant Is Defective for IPP Biosynthesis under Aerobiosis

IspG/H are essential Fe/S enzymes in *E. coli* because they participate to the isoprenoid (IPP) synthesis pathway. We therefore speculated that an *iscA sufA* mutant is not viable because IspG/H proteins are not matured. We demonstrated that this is actually the case by introducing eukaryotic genes that allow IPP synthesis from exogenously added mevalonate (hereafter referred to as the MVA pathway). The presence of this pathway allowed us to co-transduce the Δ*iscA*::*cat* mutation with the *zfh-208*::Tn*10* locus into the Δ*sufA* MVA^+^ strain DV731 (Table 2). Moreover, none of the Tet^R^ Cam^R^ transductants selected was able to grow in the absence of mevalonate. These results indicate that the heterologous MVA pathway suppresses the defects of an *iscA sufA* mutant and, by inference that the synthetic lethality under aerobiosis of combining the *iscA* and *sufA* mutations comes from a lack of IPP. It is important to underscore that we checked that none of the mutants analyzed is altered in the expression of the *ispG* or *ispH* cognate structural genes (data not shown). Hence, we can safely conclude that the molecular bases of the defects exhibited by the *iscA sufA* mutant in aerobiosis are related to a lack of maturation of the 4Fe/4S-containing IspG and/or IspH proteins.

**Table 2:** *iscA* and *sufA* mutations are synthetically lethal under aerobiosis because of defective IPP biosynthesis.

	P1/*iscA*::*cat zfh-208*::Tn*10*			
	Number of clones on LB Ara Cam		Observed co-transduction frequency after selection on LB Ara Tet	
Recipient strains	−Mev	+Mev	−Mev	+Mev_‵_
MG1655 MVA^+^	>200	>200	75%+/−5	75%+/−5
Δ*sufA* MVA^+^ (DV731)	0	>200	<1%	75%+/−5

The experiments were carried out as described in Table 2 but arabinose (Ara) was added to all the plates. Direct transduction (1^st^ and 2^sd^ column) or co-transduction experiments (3^rd^ and 4^sd^ column) were carried out in the absence or presence of mevalonate (−Mev and +Mev, respectively).

### An *iscA sufA* Double Mutant Is Viable under Anaerobic Conditions

The phenotype of an *iscA sufA* mutant, *i.e.* lethality under aerobiosis suppressible by the MVA pathway, is identical to that of an *erpA* mutant [33]. We therefore tested whether an *iscA sufA* mutant was viable under anaerobiosis, like an *erpA* mutant. In these conditions, we could co-transduce the Δ*sufA*::*kan* mutation with the *zdi-925*::Tn*10* marker into either the wild type strain or the Δ*iscA* mutant (Table 3). Conversely, we could transduce the Δ*iscA*::*cat* mutation into the wild type or the Δ*sufA* DV701 strains (Table 3). Interestingly, the Δ*sufA* Δ*iscA* transductants selected under anaerobiosis were unable to form colonies when subsequently incubated aerobically (data not shown). Similarly, the *iscA sufA* MVA^+^ strains were able to grow in the absence of mevalonate only in anaerobiosis (Table 4). These results extended the similarity between the conditional phenotype of *erpA* and *iscA sufA* mutants.

**Table 3:** *iscA* and *sufA* mutations are not synthetically lethal in the absence of oxygen.

	Number of clones on selective plates		Observed co-transduction frequency	
	P1/*sufA*::*kan*	P1/*iscA*::*cat*	P1/*sufA*::*kan zdi-925*::Tn*10*	P1/*iscA*::*cat zfh-208*::Tn*10*
Recipient strains	Selection LB Kan	Selection LB Cam	Selection LB Tet	Selection LB Tet
MG1655	>200	>200	35%+/−5	75%+/−5
Δ*sufA* (DV701)	n.d.	>200	n.d.	75%+/−5
Δ*iscA* (DV699)	>200	n.d.	35%+/−5	n.d.

The experiments were carried out as described in Table 2 but in anaerobiosis.

**Table 4:** Functional redundancy between *sufA*, *iscA* and *erpA*.

Strains	Plating efficiency in the absence of mevalonate^1^	
	−O_2_	+O_2_
MG1655 MVA^+^	≥0.5	1
Δ*sufA* MVA^+^	≥0.5	≥0.5
Δ*iscA* MVA^+^	≥0.5	≥0.5
*ΔerpA* MVA^+^	≥0.5	≤10^−5^
*ΔsufA iscA* MVA^+^	≥0.5	≤10^−4^
*ΔsufA erpA* MVA^+^	≥0.5	≤10^−5^
Δ*iscA erpA* MVA^+^	≤10^−6^	≤10^−6^
*ΔsufA* Δ*iscA ΔerpA* MVA^+^	≤10^−6^	≤10^−6^

1 All strains were grown overnight aerobically in LB medium supplemented with arabinose (0.2%), thiamine (50 µg/ml), nicotinic acid (12.5 µg/ml) and mevalonate (Mev, 1 mM) then plated on the same medium with or without mevalonate. The plates were incubated at 37°C aerobically (+O_2_) or in limiting oxygen (−O_2_) for 3 days before counting. Plating efficiency was calculated as the ratio: number of colonies formed in tested conditions/number of colonies formed aerobically on LB plates containing mevalonate. Plating efficiencies in anaerobiosis and presence of mevalonate were all ≥0.5. The numbers did not significantly change with further incubation even after 7 days and same results were obtained with overnight culture incubated anaerobically.

### Investigating Redundancies under Anaerobic Conditions

The above results showed that the double *iscA sufA* and the single *erpA* mutants are each unable to grow under aerobiosis but fully viable under anaerobiosis. To test the hypothesis that, under anaerobiosis, the *erpA* gene compensates for the lack of *iscA* and *sufA*, we transduced the *erpA*::*cat* mutation into the *sufA iscA* strain. This proved to be impossible unless the *iscA sufA* recipient contained the MVA pathway and the transductants were selected in the presence of mevalonate (data not shown). Moreover, the triple mutant *erpA iscA sufA* MVA^+^ could grow aerobically and anaerobically only in the presence of mevalonate (Table 4). This showed that the growth of the *iscA sufA* mutant under anaerobiosis depended upon the presence of a functional copy of the *erpA* gene.

Conversely, we tested whether *iscA* and/or *sufA* was required for the anaerobic growth of the *erpA* mutant. First, the *erpA sufA* strain was found to be viable since we could transduce the *erpA*::*cat* mutation into DV701 (Δ*sufA*). Moreover, an *erpA sufA* MVA^+^ strain was able to grow anaerobically even in the absence of mevalonate (Table 4). Second, in contrast to *sufA*::*kan*, the *iscA*::*cat* mutation could not be transduced into the *erpA* strain unless it contained the MVA pathway and only if transductants were selected in the presence of mevalonate. The growth of the *iscA erpA* MVA^+^ strain was totally dependent on the addition of mevalonate in the medium (Table 4). This showed that a functional copy of the *iscA* gene was absolutely required for the anaerobic growth of the *erpA* mutant whereas *sufA* appeared to be dispensable.

**Figure 3:**
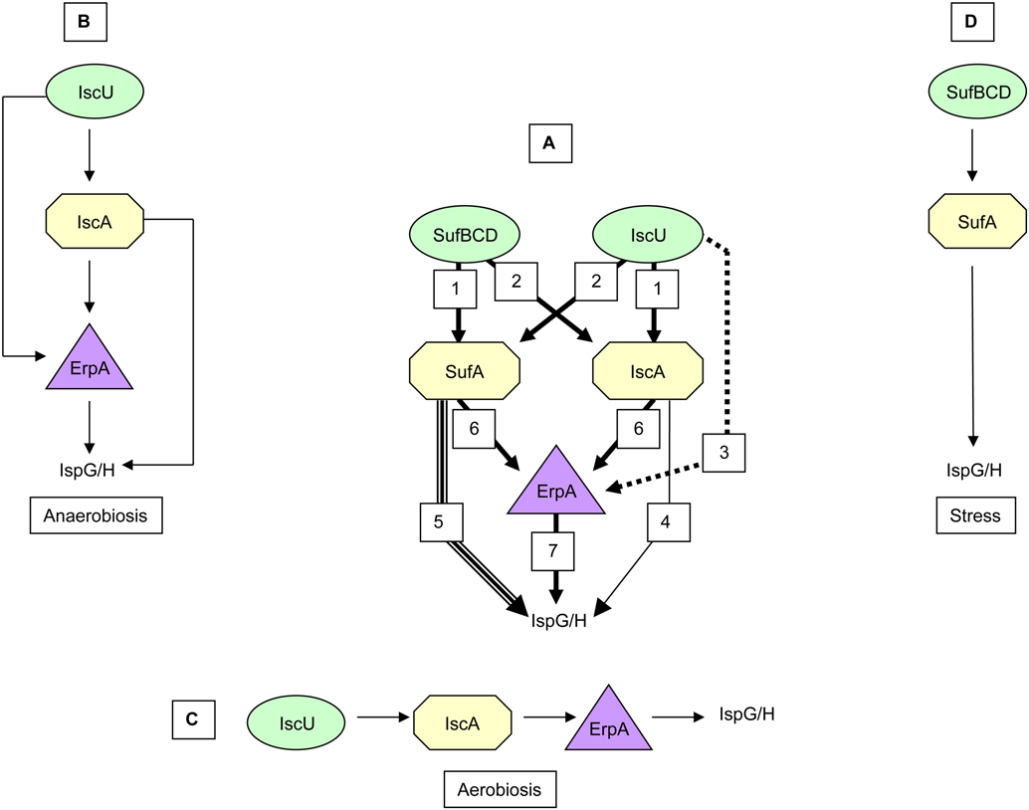
Model for Fe/S trafficking leading to IspG/H maturation in *E. coli*. Diagram depicted in panel (A) shows all possible Fe/S paths deduced from the present study and from the current literature. Path 1 was deduced from the observation that *sufA* acts as a multicopy suppressor of the *iscUA erpA* strain in a *sufB*-dependent manner and from *in vitro* studies ([48] and Fontecave's group personal communication). Path 2 was deduced from the viabilities of the *iscA sufB*, *iscA sufCD* and *iscU sufA* mutants. Path 3 was deduced from the observations that (i) *erpA* acts as a multicopy suppressor of the *iscA sufA* mutant in an *iscU*-dependent manner; (ii) the *sufA iscA* mutant is viable under anaerobiosis only in the presence of functional copies of *iscU* and *erpA*. Path 4 was deduced from the anaerobic-dependent growth of the Δ*suf erpA* mutant. Path 5 was deduced from the observation that *sufA* acts as a multicopy suppressor of *iscUA erpA*. Path 6 was deduced from the identity between the phenotypes of the *iscA sufA* and *erpA* mutants and phylogenomic analysis; other interpretations are that: (i) ErpA acts between the IscU/SufBCD and the IscA/SufA components; (ii) IscA, SufA and ErpA interact to build a heteromeric complex. Path 7 was deduced from the observation that multicopy *erpA* suppresses the *iscA sufA* conditional lethal phenotype. Diagrams depicted in panels (B), (C), (D) represent models for IspG/H maturation under different growth conditions as indicated under each panel. The situations depicted make use of a minimum number of Fe/S biogenesis components and are meant for describing a cell expressing the Fe/S biogenesis genes involved at their physiological level.

Because the inactivation of the *ispG* or the *ispH* gene is lethal in anaerobiosis, these results showed that ErpA and IscA were both able to ensure enough IspG/H maturation to produce sufficient IPP to sustain growth (Paths 4 and 7 in Figure 3A). Thus, ErpA and IscA are redundant under anaerobiosis.

### Extragenic Suppressors Strengthen the Functional Connection between *iscA*, *sufA*, and *erp*A Genes

Searching for genetic suppressors has long been a rewarding strategy for revealing functional overlap between different cellular components or pathways. We therefore used the mevalonate dependency of the strains lacking a combination of ATC encoding genes to isolate second site mutations that would render them mevalonate independent for growth.

First, we searched for secondary mutations that could suppress the double *iscA sufA* mutant. We used strain DV1145 (*sufA iscA* MVA^+^), which is mevalonate dependent for growth under aerobiosis, to select and analyze four revertants that could grow in the absence of mevalonate. The mutations that rendered strain DV1145 mevalonate independent were first localized by Hfr mapping and, second, by using a series of Tn*10* containing strains as donors in P1 transduction experiments (Materials and Methods). One suppressor mutation, referred to as *supSI-1*, was unexpectedly found within the *erpA* gene and led to a glycine-to-valine substitution at position 89 in the C-terminal part of the protein. There are several foreseeable possibilities to account for this by-pass, among which a gained (or optimized) ability to acquire Fe/S from natural (Path 3 in Figure 3A) or alternative sources, or, symmetrically, an increased ability to deliver Fe/S to IspG/H (see below, Path 7 in Figure 3A). The biochemical characterization of this variant is under way.

Second, we selected suppressors that allowed the *iscA erpA*::*cat* MVA^+^ strain to grow aerobically in LB in the absence of mevalonate. Three mutations, referred to as *supYI-1* to *3*, were found to modify the *iscR* coding sequence. Actually, in two independently selected suppressors (*supYI-1*, *supYI-2*), a single mutation changed the highly conserved Ile-37 residue to Val. The *supYI-3* mutant contained a His to Asn change at position 107. Interestingly, transducing a *sufA* but not *sufB* or *sufCD* mutation into any of these three *iscA erpA iscR*(*supYI*) mutants abolished colony formation on LB plates unless mevalonate was added, indicating that the suppressive effect of the *iscR* mutant alleles depended on an active *sufA* gene. Moreover, the expression of a P*_suf+_*::*lacZ* gene fusion (see Materials and Methods) was found to be induced in the presence of the *supYI* suppressor mutation (data not shown). Taken together, these results indicated that suppression of the mevalonate-dependence of the *iscA erpA* mutant was due to an increased expression of the IscR-activated *suf* operon, hence an increase in SufA protein synthesis (Path 5 in Figure 3A).

A fourth revertant (*supYI-4*) was found to be a single G-to-T substitution at position −27 in the promoter sequence of *sufA*. Remarkably, this mutation altered the putative binding site for the Fur repressor. A possibility was that this mutation led to a derepression of the *suf* operon, thereby increasing SufA protein synthesis (Path 5 in Figure 3A). This hypothesis was confirmed by fusing this mutant promoter sequence to a *lacZ* reporter gene (see Materials and Methods): the expression level of P*_suf_*_(*supYI-4*)_::*lacZ* in a wild type genetic context was about 150-fold higher than the expression level of the P*_suf+_*::*lacZ* fusion.

Overall, this hunt for extragenic suppressors fully confirmed that the three genes *iscA*, *sufA* and *erpA* are interchangeable. These data also revealed that, *in vivo*, one barrier to full functional redundancy lies in gene expression control.

### Analysis of ATC Redundancy via a Multicopy Suppressor Approach

The genetic analysis above suggested that increased synthesis of a given ATC protein could create bypasses, compensating for the loss of the others. We therefore investigated the potential redundancies between ErpA, IscA and SufA by a multicopy suppressor approach. Likewise, the *iscA* and *sufA* genes were each cloned under the control of the P*_ara_* promoter in the pBAD vector, and the resulting plasmids were tested for their ability to suppress the lethality caused by the *erpA* mutation under aerobiosis. The pLAS-A plasmid (SufA overproducer) suppressed the growth defect of strain LL402 (*erpA*), although not with wild type efficiency (Table 5). In contrast, the pLAI-A plasmid (IscA overproducer) was unable to suppress the *erpA* mutant's lethal phenotype (data not shown). A series of additional genetic experiments further strengthened the notion of a difference between IscA and SufA. For instance, transduction experiments showed that the otherwise lethal *erpA*::*cat* mutation could be transduced into MG1655 under aerobiosis if the recipient carried the pLAS-A plasmid, whereas no transductants were obtained if MG1655 carried pLAI-A (data not shown). Also, a genomic *E. coli* pUC-based library was screened for plasmids able to restore the growth of the Δ*erpA* mutant under aerobiosis. Of nine suppressing plasmids recovered, five carried the *erpA* gene, four carried the *sufA* gene, but none carried *iscA* (data not shown). These studies indicated that increased *sufA* gene dosage was able to sustain the aerobic growth of an *erpA* mutant (Path 5 in Figure 3A).

**Table 5:** Multicopy of *sufA* suppresses the phenotype of the *iscA erpA* double mutant *via* IscU or *via* the SUF system.

Strains	Plating efficiency in the absence of mevalonate^1^	
	−O_2_	+O_2_
Δ*erpA*::*cat* MVA^+^/pBAD^2^	≥0.5	≤10^−5^
Δ*erpA*::*cat* MVA^+^/pLAS-A^2^	≥0.5	≥0.5
Δ*iscA* Δ*erpA*::*cat* MVA^+^/pBAD	≤10^−6^	≤10^−6^
Δ*iscA* Δ*erpA*::*cat* MVA^+^/pLAS-A	≥0.5	≥0.5
*ΔiscUA ΔerpA*::*cat* MVA^+^/pBAD	≤10^−6^	≤10^−6^
*ΔiscUA ΔerpA*::*cat* MVA^+^/pLAS-A	≥0.5	≥0.5
Δ*iscA* Δ*suf* Δ*erpA*::*cat* MVA^+^/pBAD	≤10^−5^	≤10^−6^
Δ*iscA* Δ*suf* Δ*erpA*::*cat* MVA^+^/pLAS-A	≥0.5	≥0.5
Δ*suf*::*cat* Δ*iscA* MVA^+^/pBAD	≥0.5	≤10^−5^
Δ*suf*::*cat* Δ*iscA* MVA^+^/pLAS-A	≥0.5	≥0.5
Δ*suf*::*cat* Δ*iscUA* MVA^+^/pBAD	≤10^−6^	≤10^−6^
Δ*suf*::*cat* Δ*iscUA* MVA^+^/pLAS-A	≤10^−6^	≤10^−6^

1 Plating efficiency was calculated as in Table 6. Ampicillin, thiamine, nicotinic acid and arabinose were added to all plates. Plating efficiencies in anaerobiosis and presence of mevalonate were all ≥0.5.

2 Similar results were obtained with the Δ*sufA ΔerpA*::*cat* MVA^+^ strain.

Conversely, we tested whether increased *erpA* gene dosage was able to suppress the lethality of the *iscA sufA* mutant. Strain DV731 (*iscA sufA* MVA^+^) was grown in the presence of mevalonate and transformed with plasmid pLAE-A (ErpA overproducer). The transformants became able to grow under aerobiosis, even in the absence of added mevalonate (Table 5). Moreover, we were able to transduce the *iscA*::*cat* allele into strain DV701 (Δ*sufA*) carrying plasmid pLAE-A. Thus, these results indicate that ErpA, when overproduced, compensates for the defect of IPP synthesis due to the simultaneous absence of both SufA and IscA under aerobic conditions (Path 3 or 7 in Figure 3A).

*S. cerevisiae* encodes two ATCs, ISA1 and ISA2. Heterologous complementation was tested using the *E. coli* strains *erpA* and *sufA iscA* mutants. The plasmid p(Isa1) complemented *sufA iscA* but not *erpA*, like the pLAI-A plasmid carrying the *E. coli iscA* gene. The plasmid p(Isa2) complemented both the *iscA sufA* and the *erpA* mutants, like the pLAE-A plasmid carrying the *E. coli erpA* gene. These results were consistent with the classification of ISA1 and ISA2 being an ATC-II and ATC-I, respectively.

### Defining Two Separate Pathways for Maturation of IspG/IspH

Both the extragenic and the multicopy suppressor approaches provided data pointing to potential interchangeability among the different ATCs that the cell can use for IspG/H maturation. We were therefore interested in defining the genetic constraints required for these suppressing effects to occur. In particular, it was of great importance to identify which component of the cellular Fe/S biogenesis system (see Introduction) was required in each of the suppression cases described above.

We thus first sought to construct a set of strains in which deletions of each *suf* gene were combined with deletions of each *isc* gene. In transduction experiments similar to those described above to assess *iscA* and *sufA* incompatibility, we found that, under aerobiosis, the Δ*iscA* mutation could be combined with a deletion of any of the *suf* genes (*sufB*, *sufCD*, *sufS* and *sufE* were tested).

Conversely, the Δ*sufA* mutation could be introduced without loss of viability into the *iscU*, *iscS*, *fdx*, *hscA* and *hscB* mutants (data not shown). On the contrary, the *iscUA* deletion inactivating both *iscA* and *iscU* could not be combined with any *suf* mutation. Likewise, the Δ*suf* mutation deleting the whole *suf* operon was not compatible with a mutation in any of the *isc* genes. In addition, as was the case for the combination of *iscA* and *sufA* mutations, all combinations of mutations became possible in the presence of the MVA pathway genes in the strain and the addition of mevalonate in the medium. All together, these results indicate that IscA can function with the SUF system and, conversely, SufA can function with the ISC system (Path 2 in Figure 3A), consistent with the association of ATC-II encoding genes with either the *isc* or the *suf* operon in Alpha-Proteobacteria (Figure 1).

We then tested which elements were required for *sufA* to act as a multicopy suppressor of the *iscA erpA* double mutant (Table 6). The ability of pLAS-A to suppress the *iscA erpA* strain remained possible even in the absence of IscU because pLAS-A suppressed the lethality of the *iscUA erpA* strain (Table 6). This indicated that *sufA* could function in the absence of the ISC system. In addition, pLAS-A could suppress the lethality of the *iscA erpA* strain in the absence of other *suf* genes, *i.e.* strain *iscA erpA* Δ*suf*/pLAS-A was viable (Path 5 in Figure 3A). In fact, *iscA erpA* suppression by pLAS-A could be abolished only in strain *iscUA* Δ*suf erpA*. These results confirmed that SufA could function, not only with its “natural” partners of the SUF system, but also with IscU in the ISC system.

**Table 6:** Increased *erpA* gene dosage suppresses the *sufA iscA* double mutant phenotype *via* IscU only.

Strains	Plating efficiency in the absence of mevalonate^1^	
	−O_2_	+O_2_
Δ*sufA* Δ*iscA* MVA^+^/pBAD	≥0.5	≤10^−4^
Δ*sufA* Δ*iscA* MVA^+^/pLAE-A	≥0.5	≥0.5
Δ*suf*::*cat* Δ*iscA* MVA^+^/pBAD	≥0.5	≤10^−5^
Δ*suf*::*cat* Δ*iscA* MVA^+^/pLAE-A	≥0.5	≥0.5
*ΔsufA* Δ*iscUA* MVA^+^/pBAD	≤10^−5^	≤10^−5^
*ΔsufA* Δ*iscUA* MVA^+^/pLAE-A	≤10^−5^	≤10^−5^
Δ*suf*::*cat* Δ*iscUA* MVA^+^/pBAD	≤10^−6^	≤10^−6^
Δ*suf*::*cat* Δ*iscUA* MVA^+^/pLAE-A	≤10^−6^	≤10^−6^

1 Plating efficiencies were calculated as in Table 6. Plating efficiencies in anaerobiosis and presence of mevalonate were all ≥0.5.

We showed previously that the growth of the *iscA sufA* strain in the absence of O_2_ was *erpA* dependent, because the growth of the triple mutant *iscA erpA sufA* strictly depended on the presence of mevalonate (Table 3). An interesting observation was that the growth of *iscA sufA* in the absence of O_2_ was also IscU-dependent since the *iscUA sufA* strain (and Δ*suf iscUA*) was unable to grow in the absence of O_2_ unless mevalonate was added in the medium (Table 6). Altogether, these results indicated that the growth of the *iscA sufA* strain in anaerobiosis likely requires an IscU-ErpA connection (Path 3 in Figure 3A). In the absence of the *suf* operon, plasmid pLAE-A suppressed the lethality associated with the simultaneous inactivation of the *iscA* and *sufA* genes (Table 6). This indicated that ErpA-mediated suppression of the *iscA sufA* strain was Suf independent. On the contrary, ErpA-mediated suppression of *iscA sufA* was *iscU* dependent since plasmid pLAE-A lost its suppressive ability with the additional inactivation of *iscU*, *i.e.* strains *iscUA sufA*/pErpA or *iscUA* Δ*suf*/pErpA were inviable in the absence of mevalonate. This strengthened the hypothesis that ErpA could work with IscU (Path 3 in Figure 3) and, moreover, strongly suggested that ErpA could not use the SUF system without SufA.

## Discussion

Fe/S proteins rank among the most versatile proteins throughout all living organisms. Our present understanding of the biogenesis of Fe/S clusters rests mainly on the analysis of two multiprotein systems, ISC and SUF, present in model organisms. Among these proteins, the actual cellular role and biochemical function of the ATC proteins has been the subject of some debate. Here, results from a combined phylogenomic and genetic investigation allow us (i) to trace the emergence of multiple ATCs; (ii) to position ATCs as “carriers” acting between scaffolds and apotargets and (iii) to propose a model explaining how the cell controls and exploits potential ATC redundancy to build up different Fe/S trafficking routes in order to meet changes in environmental conditions.

### *sufA iscA* Conditional Lethality Is Most Likely Due to Insufficient Maturation of IPP Synthesizing Enzymes IspG/H

The *iscA* and *sufA* single mutants have repeatedly been found to cause marginal defects [5],[6]. However, we find here the *iscA sufA* double mutant to be conditional lethal in aerobiosis, in agreement with recent reports [36],[40]. In contrast, Lu *et al.* have found the *sufA iscA* double mutant to be non viable in synthetic medium but viable when aerobically cultivated in rich medium LB [35]. One reason for the discrepancy between these studies might lie in the fact that the *sufA iscA* strains generate survivors at a relatively high frequency when cultivated aerobically as we showed here (Table 3, Materials and Methods). Alternatively, the discrepancy might be due to differences in the genetic background: the *sufA/iscA* combination was found conditionally lethal in MG1655 whereas Lu *et al.* [35] worked with MC4100, a strain which went through multiple mutagenesis protocols [41].

The conditional phenotype of an *iscA sufA* mutant could formally be due to the general alteration of the SUF and ISC systems. This hypothesis, however, runs against a series of experimental observations reported in the present and previous studies: (i) analysis of the SUF system revealed the existence of various types of complexes such as SufBCD, SufSE, SufBCDSE, none of them containing SufA ([13],[42], our unpublished results); hence, a mutation in *sufA* is not predicted to destabilize the entire Suf complex and, indeed, mutations in *sufA* cause phenotypes much less severe than mutations in other *suf* genes; (ii) IscA was indeed found to interact with HscA and, because HscA was found to form a complex with IscSUHscB, a formal possibility is that a defect in IscA could alter the functioning of the ISC system; however, this is difficult to reconcile with the fact that a mutation in *iscA* causes minor phenotype, if at all, compared with mutations in other *isc* genes [12],[43]; (iii) control experiments allowed us to rule out the hypothesis of a polar effect of the *iscA* or *sufA* mutations on the expression of downstream *isc* or *suf* genes. Rather, we showed unambiguously in this work that insufficient synthesis of IPP is the cause of the lethality of the *sufA iscA* mutant. A similar lack of IPP had been put forward to account for the conditional phenotype of the *erpA* mutant [33]. The last steps of the synthesis of these compounds in *E. coli* involve two essential enzymes, IspG and IspH, which are known to contain 4Fe/4S clusters necessary for their activity [44],[45]. *erpA* gene expression was not altered in the *sufA iscA* mutant, and conversely, no decrease of *iscA* and *sufA* mRNA was observed in the *erpA* mutant (data not shown). Along the same lines, *ispG* and *ispH* genes expression was not altered by mutations in *sufA*, *iscA*, *erpA* or combinations thereof (data not shown). Therefore, we conclude that a defect in IspG/H Fe/S maturation is likely the cause of the lack of growth of *iscA sufA* and *erpA* mutants. Together with our previous characterization of ErpA, these observations reinforce the idea that IspG/H are the only Fe/S enzymes requiring ATCs essential for *E. coli* to survive under routine laboratory conditions, and that all three ATCs contribute to the maturation of IspG/H. Mettler *et al.* recently proposed that the ErpA activity was responsible for the viability of *iscA sufA* double mutant under anaerobiosis [40]. The present work, in which we showed the lethal phenotype of the *iscA sufA erpA* triple mutant, fully confirmed this interpretation.

### ATC Function as Fe/S Carriers

On the basis of *in vitro* studies, ATCs were first proposed to act as scaffolds [46], because they were shown to bind and eventually transfer Fe/S clusters to a wide series of apotargets, including 2Fe/2S proteins like Fdx and 4Fe/4S proteins like BioB, IspG and APS reductase [33], [47]–[49]. Later on, this view was challenged by a series of studies reporting the ability of IscA and SufA to bind *in vitro* iron only, and by the proposal that they acted as iron sources *in vivo* [35],[50],[51]. In turn, the “iron only” hypothesis was disputed by the discovery that the as-isolated IscA protein from *Acidithiobacillus ferrooxidans* contains a 4Fe/4S cluster [52] and by a new study on *E. coli* SufA [53]. Last, an *in vitro* study showed that IscU could assist Fe/S acquisition by IscA but the reverse was not true, indicating that IscA might intervene downstream of the IscU-controlled scaffolding step [48]. Our genetic analysis in *E. coli* showed that maturation of IspG/H could not take place in two situations: (i) the complete absence of the three ATCs and (ii) the simultaneous absence of IscU and the SufBCD complex. Conversely, in the presence of one ATC protein and IscU or SufBCD, we were always able to find at least one experimental condition supporting IspG/H maturation. The fact that the inactivation of *iscU* and *sufBCD* is lethal is fully consistent with the proposal that SufBCD acts as a scaffold within the SUF system [30]. A remote possibility is that SufBCD is necessary for the functioning of an as yet unidentified scaffold. Furthermore, the viability of the *iscU sufA* and *iscA sufB* (or *sufCD*) mutants indicates that the ATC proteins intervene at a different level than IscU/SufBCD. This suggests that ATCs are unlikely to have a scaffolding function, a conclusion also reached by other authors [54]. Our results are thus in agreement with a role of the ATCs as intermediates between the Fe/S synthesis systems and the apotargets, likely as carriers of ready made clusters (Figure 3). Despite their established involvement in the maturation of Fe/S proteins in yeast, the molecular function of ISA1 and ISA2 remains obscure. In particular, their ability to bind Fe/S clusters appears uncertain [8]. Importantly, we found *S. cerevisiae* ATCs ISA1 and ISA2 able to complement a lack of ATCs in *E. coli*, hence to presumably act as Fe/S carriers. Studies are however required to test the tentative idea that ISA1/2 can fulfil in the bacterial context a function that they do not carry on in the yeast mitochondria.

### Specificity and Redundancy among the ATCs

A mutant devoid of all three ATCs did not permit maturation of IspG/H. Furthermore, we were able to identify at least one experimental condition under which a single ATC could ensure apo-IspG/H maturation in the absence of the two other ATCs. Taken together, these two observations are best explained by postulating that all three ATCs are intrinsically able to mature IspG/H. The ATC proteins are thus clearly biochemically redundant, in agreement with results from the *in vitro* assays and the common evolutionary origin of their encoding genes.

The question then arises as to why the *erpA*, *erpA sufA* and *iscA sufA* mutants, which all produce at least one ATC, could not mature IspG/H under aerobiosis but could do so under anaerobiosis. One possibility is that under aerobiosis, the cellular need in IPP exceeds that under anaerobiosis and that the concentration of active IspG/H in these mutants is below the critical minimal level necessary for growth. The fact that overproduction of ErpA or SufA is sufficient to restore the growth of the *iscA sufA* or *iscA erpA* mutants, respectively, indeed supports the hypothesis that the limiting step for IPP synthesis in these mutants is the activity carried out by ATCs, namely maturation of IspG/H. However, this interpretation does not explain why oversynthesis of IscA does not allow IspG/H maturation (*i.e. iscA* does not act as a multicopy suppressor of the *erpA* mutant). This last observation indicates that, in the presence of O_2_, IscA does require ErpA to make active IspG/H. Hence, this leads us to propose a model in which the environment controls the specificity of partnerships. The molecular bases of this environmental control appear to lie either directly within the transfer process itself or with gene regulation. Under environmental circumstances that are harmless for Fe/S (Figure 3B), such as anaerobiosis, the IscU scaffold transfers its Fe/S to either ErpA or IscA, both of which can transfer the cluster to apo-IspG/H. In addition, ErpA can receive an Fe/S cluster from IscA. Biochemically, SufA is also potentially able to carry out this activity, but it is not synthesized in sufficiently large amounts in these conditions. A direct transfer of an Fe/S cluster from IscU to apo-IspG/H, bypassing IscA and ErpA, does not occur. When conditions are more hazardous for Fe/S cluster synthesis (Figure 3C), such as aerobiosis, IscA and ErpA cooperate to mature apo-IspG/H, presumably because of the O_2_-sensitivity of the direct transfer from IscA to apo-IspG/H and the poor synthesis of the SUF system. When conditions become stressful (Figure 3D), SufA recruits the whole SUF system for building up a new pathway for maturation of apo-IspG/H.

The model above derives from the interpretation of the phenotypes of mutants and of the effects of plasmids overproducing ATCs. Because we did not directly measure the level of each ATC protein, no definitive conclusion can thus be made on how efficient each ATC protein functions in a particular path in a wild type strain. For example, the transfer of Fe/S clusters from IscU to SufA was put forward to account for the viability of the *iscA sufB* strain. However, in this strain, the *sufA* gene expression is increased because of the *iscA* mutation: the transfer might not occur in a wild type strain with “normal” level of IscA and SufA. Moreover, showing that the proteins are present in normal amounts might not be conclusive either. For instance, a transfer from IscU to ErpA was proposed to occur in the *iscA sufA* strain but, again, such a transfer ought to be demonstrated to occur in the presence of a competing IscA protein. Hence, our model aims at listing all potential paths for transferring Fe/S from and to ATCs. The next challenge will be to identify conditions under which each path operates.

The ISC and SUF systems have been proposed for a long time to overlap and we showed here that they indeed share a common substrate, *i.e.* IspG/H. This overlap accounts for both the lethality of *isc suf* mutants [12],[36],[40],[55] and for the suppressing effect of overproducing the *suf* operon in *isc* mutants [12],[40]. On the other hand, recent data revealed that the maturation of Fnr is under the control of ISC only under aerobiosis [40]. Also, in *Azotobacter*, IscA is required for survival at high oxygen tension, implying that an Fe/S protein, essential for growth under these conditions, is specifically targeted by IscA [34]. Finally, in *Azotobacter*, IscU can be substituted under low oxygen tension by NifU, the scaffold of the NIF system [56]. Collectively, these observations indicate that substrate specificity might also intervene in deciding which Fe/S biogenesis pathway should be used for a given apoprotein. Clearly, more work is required to define the extent of the overlap between the Isc and Suf pathways. Solving the issue will require, in particular, the *in vivo* study of the maturation of a large set of Fe/S proteins, if not all, in various conditions and genetic backgrounds. Interestingly, it was recently suggested that the maturation of the 4Fe/4S proteins and 2Fe/2S proteins might follow different pathways [57].

### ATCs History

The present phylogenomic study lends credence to an evolutionary scenario in which ATC originated in bacteria and were conserved in main bacterial phyla (Figure 1, circle number 0 and pink filled-in diamonds). Subsequently, Alpha-, Beta- and Gamma-Proteobacteria acquired a second ATC gene *via* either gene duplication or horizontal gene transfer, and the two ATCs were later transferred to eukaryotes *via* the mitochondrial endosymbiosis. In proteobacteria and eukaryotes, ATCs were subdivided in two sub-families: ATC-I that contains the *E. coli* ErpA and ISA2 proteins, and ATC-II that contains the *E. coli* IscA and SufA and the *S. cerevisiae* ISA1 proteins. Based on genetic experiments, we propose that in *E. coli* at least, ATC-I became specialized in the transfer of Fe/S to apotargets, while the selection pressure exerted on ATC-IIs tended to keep them in close partnership with proteins of the Fe/S synthesis systems from which they receive Fe/S clusters (Figure 3). This proposal is supported by the fact that: (i) functional analysis of the *E. coli* system showed that Fe/S transfer does not occur between the ATC-I protein ErpA and SufBCD proteins of the SUF system, and is inefficient between ErpA and IscU (Path 3 in Figure 3); on the contrary, both *E. coli* ATC-II proteins IscA and SufA can function with the two systems, and (ii) the genomic context of ATC-I encoding genes is devoid of genes involved in Fe/S biosynthesis, in contrast to ATC-II encoding genes, always found associated with such genes in proteobacteria. This scenario led us to speculate that ATC-I (ErpA) intervenes at a different step from ATC-II (IscA/SufA) in the IspG/H maturation process in *E. coli* (Figure 3).

### Perspectives

Three main conclusions can be drawn from studying *E. coli* as a model: (i) ATCs are essential intermediates between Fe/S biogenesis systems and targets, (ii) ATCs possess highly related biochemical properties, and (iii) the seemingly redundant repertoire of ATCs is exploited by the cell as a function of environmental conditions and possibly substrate specificity. The phylogenomic analysis uncovered a wide diversity in the distribution of ATCs. Thus, the comparison of the *E. coli* system with these widely diverging systems yields immediate questions, which *E. coli* could help to solve both as a reference and as a tool. Hereafter are listed a few situations we think might be of great interest to investigate. First, there are organisms (*e.g.* some Archaea, PVC, Spirochaetes, Thermotogae) that lack ATC but encode SUF or ISC components, IscU and/or SufB/D proteins, in particular. It is conceivable that the scaffold(s) of these organisms evolved in such a way that they acquired the ability to directly transfer Fe/S clusters to apotargets, or, alternatively, that other proteins carry out the function of ATCs. One way to understand these cases would be to test whether IscU from ATC-less organisms can complement the lethality of an *E. coli* strain devoid of ATC and identify the determinants differentiating these IscUs and *E. coli* IscU. Second, there are organisms having only one ATC. This latter might have conserved its ancestral ability to interact with both Fe/S biogenesis systems and apotargets. Firmicutes rank among them and it will be a feasible task to test this prediction by using *Bacillus subtilis* as a model. Third, there are organisms that possess multiple ATCs, but with a repertoire different from that of *E. coli*. This is the case of *Anaebena*, which has 2 ATC-IIIs in addition to two ATCs, or *Rhodospirillum*, which has one ATC-I, one ATC-II and one ATC-III. This raises the question whether, like *E. coli*, these organisms have exploited their repertoire of ATCs by dedicating some ATCs to an interaction with apotargets while other ATCs were kept in closer connection with general components such as scaffolds. ATC-IIIs represent an interesting case since they seem to have evolved to become specific for a particular target, nitrogenase. It would be interesting to know whether ATC-III also evolved to receive Fe/S solely from the NIF system.

## Materials and Methods

### Media and Growth Conditions

The rich and synthetic media were LB broth and M9, respectively. Glucose (0.2%), arabinose (0.2%), casaminoacids (0.2%), amino acids (0.005%), thiamine (50 µg/ml), nicotinic acid (12.5 µg/ml) and mevalonate (1 mM) were added when required. Solid media contained 1.5% agar. Antibiotics were used at the following concentrations: chloramphenicol (Cam) 25 µg/ml, kanamycin (Kan) 25 µg/ml, tetracycline (Tet) 25 µg/ml, spectinomycin (Spc) 50 µg/ml and ampicillin (Amp) 50 µg/ml.

### Bacterial Strains, Phages, and Plasmids

All the strains are *E. coli* K-12 derivatives; principal strains are listed in Table 7. Strains carrying the Tn*10* transposons used for co-transduction experiments were from the Singer collection [58],[59]; strain JW1674 from the Keio collection [60] kindly provided by P. Moreau, was the donor of the Δ*sufA*::*kan* mutation; the same collection provided all the other *suf* mutations but Δ*suf*::*cat* and all the deletions of the *isc* genes but *iscA*::*cat* and *iscUA*::*cat*.

**Table 7:** Bacterial strains and plasmids.

Strain	Relevant genotype	Origin or construction
MG1655	Parental strain	Laboratory collection
DV1093	MVA^+^	MG1655+P1/EcAB1-5 [76], selection LB Kan
DV1094	Δ*erpA*::*cat* MVA^+^	DV1093+P1/LL402 [33], selection LB Cam ara mev +O_2_
DV1221	Δ*sufA*::*kan*	MG1655+P1/JW1674 [60], selection LB kan
DV698	Δ*iscA*::*cat*	See Materials and Methods
DV597	Δ*iscUA*::*cat*	See Materials and Methods
DV595	Δ*suf*::*cat* (*sufABCSE*::*cat*)	[24]
DV1239	Δ*iscA*::*cat zfh*-*208*::Tn*10*	DV698+P1/CAG18481 [58],[59], selection LB Tet. Clone Cam^R^
DV1230	Δ*sufA*::*kan zdi-925*::Tn*10*	DV1221+P1/CAG12151 [58],[59], selection LB Tet. Clone Cam^R^
DV699	Δ*iscA*	DV698 cured with pCP20. Clone Cam^S^
DV599	Δ*iscUA*	DV597 cured with pCP20. Clone Cam^S^
DV701	Δ*sufA*	DV1221 cured with pCP20. Clone Cam^S^
DV706	Δ*suf*	DV595 cured with pCP20. Clone Cam^S^
DV1199	Δ*erpA* MVA^+^	DV1094 cured with pCP20. Clone Cam^S^
DV700	Δ*iscA* MVA^+^	DV699+P1/DV1093, selection LB Kan
DV1200	Δ*iscUA* MVA^+^	DV599+P1/DV1093, selection LB Kan
DV731	Δ*sufA* MVA^+^	DV701+P1/DV1093, selection LB Kan
DV736	Δ*suf* MVA^+^	DV706+P1/DV1093, selection LB Kan
DV1145	Δ*sufA ΔiscA*::*cat* MVA^+^	DV731+P1/DV698, selection LB Cam ara mev +O_2_
DV1139	Δ*sufA* Δ*erpA*::*cat* MVA^+^	DV731+P1/DV1094, selection LB Cam ara mev +O_2_
DV1151	Δ*iscA* Δ*erpA*::*cat* MVA^+^	DV700+P1/DV1094, selection LB Cam ara mev +O_2_
DV1256	Δ*sufA ΔiscA* MVA^+^	DV1145 cured with pCP20. Clone Cam^S^
DV1257	Δ*iscA* MVA^+^ Δ*suf*::*cat*	DV700+P1/DV595, selection LB Cam ara mev +O_2_
DV1259	Δ*iscUA* MVA^+^ Δ*suf*::*cat*	DV1200+P1/DV595, selection LB Cam ara mev +O_2_
DV1258	Δ*iscA* MVA^+^ Δ*suf*	DV1257 cured with pCP20. Clone Cam^S^
DV1260	Δ*iscUA* MVA^+^ Δ*suf*	DV1259 cured with pCP20. Clone Cam^S^
DV1261	Δ*sufA* Δ*iscA erpA*::*cat* MVA^+^	DV1256+P1/DV1094, selection LB Cam ara mev +O_2_
DV1262	Δ*iscA* MVA^+^ Δ*suf* Δ*erpA*::*cat*	DV1258+P1/DV1094, selection LB Cam ara mev +O_2_
DV1263	Δ*iscUA* MVA^+^ Δ*suf* Δ*erpA*::*cat*	DV1260+P1/DV1094, selection LB Cam ara mev +O_2_
DV1294	Δ*lacZ* P*_suf+_*::*lacZ*	See Materials and Methods
DV1296	Δ*lacZ* P*_suf(YI-4)_*::*lacZ*	See Materials and Methods
pLAI-A	P*ara*::*iscA*, Amp^R^	pBAD-I* derivative (see Materials and Methods)
pLAS-A	P*ara*::*sufA*, Amp^R^	pBAD-I* derivative (see Materials and Methods)
pLAE-A	P*ara*::*erpA*, Amp^R^	pBAD-I* derivative (see Materials and Methods)
p(ISA1)	P*ara*::*erpA*, Amp^R^	pBAD-I* derivative (see Materials and Methods)
p(ISA2)	P*ara*::*erpA*, Amp^R^	pBAD-I* derivative (see Materials and Methods)

The *iscA*::*cat* mutation was generated in a one-step inactivation of the *iscA* gene as described [61]. A DNA fragment containing the *cat* gene flanked with a 5′ and 3′region bordering the *iscA* gene was PCR-amplified using pKD3 as a template and oligonucleotides iscAUP and iscADO (Table S2). Strain BW25113, carrying the pKD46 plasmid, was transformed by electroporation with the amplified linear fragment and Cam^R^ clones were selected; one of these clones was used to transduce the *iscA*::*cat* mutation into MG1655, giving strain DV698. To generate the *iscUA*::*cat* deletion, we first cloned the *iscUA* genes by PCR amplification from *E. coli* MG1655 chromosomal DNA using oligonucleotides iscU and iscA (Table S2) and subsequent insertion of the PCR product in pGEM-T deleted for the *Hinc*II restriction site by the T/A cloning method. The two genes were then disrupted by inserting the *cat* cassette in the coding region between the two *Hinc*II sites of *iscUA*. The resulting *iscUA*::*cat* disruption was then excised by restriction and the linear DNA electroporated into the BW25113/pKD46 strain; one Cam^R^ clone was used to transfer the *iscUA*::*cat* deletion into MG1655 creating strain DV1240. All mutations were introduced into strains by P1 *vir* transduction [62], selecting for the appropriate antibiotic resistance. The antibiotic resistance cassettes were eliminated when needed using plasmid pCP20 as described [63].

To verify that the *isc* and *suf* mutations had no polar effects, we checked that the *sufE* and *hscA* genes were correctly transcribed in the mutants. We first extracted the RNA from mutant strains grown overnight in LB medium. RNA was extracted using the SV Total RNA Isolation System according to the manufacturer's recommendations. To remove further DNA contaminations, the eluted RNA was treated with 2 units of RNase-free DNase and kept at −80°C. The amount of RNA was calculated from 260 nm absorption using a Biowave II spectrophotometer. Conversion of RNA to cDNA was performed using the SuperScript RT System from Invitrogen. For all RT reactions, 1 µg RNA was used and 100 ng random primer was added together with diethylpyrocarbonate (DEPC)-treated water to a final volume of 12 µl. The mixture was then incubated at 70°C for 10 min and transferred to room temperature. To continue the reverse transcription reaction, a master mix was prepared according to the manufacturers protocol, added to the RNA tube and incubated at 42°C for 1 hour, followed by a 15 min inactivation step at 70°C in a Robocycler. cDNA was kept at −80°C.

PCR were carried out in a standard PCR Master Mix reaction with 1/30 of the reverse transcription reactions and 100 ng of primers hscARevRT and hscAsensRT or sufErevRT and sufEsensRT in 10 µl final volume (Figure S2A and B). Lack of contaminating DNA from the RNA preparations was checked by performing parallel PCR reactions using RNA at a same concentration (Figure S2C). DNA products were analyzed by 1.5% agarose gel electrophoresis with Tris-acetate-EDTA (TAE) buffer.

To construct the P*_suf_*::*lacZ* fusions, PCR amplifications of chromosomal DNA from strains *iscA erpA* MVA^+^ and *iscA erpA* MVA^+^ *sup*(*YI-4*) were carried out using primers sufUP and sufDO (Table S2). The PCR products were digested by *Eco*R1 and *Bam*HI and cloned into pRS415 [64] to construct transcriptional fusions. The fusions were introduced into the bacterial chromosome of strain DV206 (MG1655 Δ*lacZ*, [65]) using the RS45 phage and we verified that strains DV1294 (MG1655 Δ*lacZ* P*_suf_*_+_::*lacZ*) and DV1296 (MG1655 Δ*lacZ* P*_suf(YI-4)_*::*lacZ*) were monolysogens as described [66]. ß-galactosidase assays were carried out as described [62]. When grown in LB to OD600 about 0.5, the activity of the P*_suf+_*::l*acZ* and P*_suf_*_(*YI-4*)_::*lacZ* fusions was 4+/−10% and 600+/−10%, respectively.

Plasmids pLAI-A (IscA), pLAS-A (SufA) and pLAE-A (ErpA) were constructed by, first, PCR amplification from the MG1655 chromosomal DNA using the following primers: EcoIscA and XhoIscA for pLAI-A; EcoSufA and XhoSufA for pLAS-A; EcoErpA and XhoErpA for pLAE-A (Table S2). The PCR products were then digested by *Eco*RI and *Xho*I and ligated into the cognate sites of pBAD-I* (Amp^R^). Plasmids p(ISA1) and p(ISA2) were constructed by, first, PCR amplification from pETDuet-1(*isa1*) with primers T7 and XhoIsa1 or PCR amplification from pE-T3a-(*isa2*) with primers T7 and XhoIsa2, respectively, and subsequent T/A cloning in pGEM-T. *Xba*I-*Xho*I fragments carrying the *isa1* or *isa2* genes were then subcloned from the pGEM-T derivatives into the *Nhe*I/*Sal*I sites of pBAD-I*. This allowed the synthesis ISA1 and ISA2 proteins free of mitochondrial targeting signal.

### Suppressors Selection and Localization

In the presence of exogenously added mevalonate, the DV1145 strain (*sufA iscA* MVA^+^) forms tiny colonies after 24 h aerobic incubation. However, we noticed that fast growing clones were often seen in the streaks, indicating that suppressor mutations accumulated at high frequency. To select suppressors, we grew the DV1145 strain under anaerobiosis in the presence of MVA, then plated it onto LB plates that were incubated aerobically at 37°C. Plating efficiency in LB compared to mevalonate-supplemented LB was about 10^−4^ (Table 2). Suppressor mutants were purified twice from the LB plates after two days incubation and four independent mutants (*supSI-1* to *-4*) were retained for further analysis. Four suppressors *supYI-1* to *-4* of *iscA erpA*::*cat* MVA^+^, occurring at a lower frequency (10^−6^, Table 2), were selected in a similar way.

Strains *iscA erpA*::*cat* MVA^+^ *supYI* and *sufA iscA*::*cat* MVA^+^ *supSI* were crossed with various Hfr Tn*10* strains from the Wanner collection [67] kindly provided by M. Berlyn and the exconjugants were selected onto LB medium plates supplemented with Cam, Kan Tet arabinose and mevalonate. The exconjugants were then scored for their ability to grow in the absence of mevalonate, which indicated the presence of suppressor mutations. These experiments showed that the *supSI-1^+^* allele could be transferred at a very high frequency by the HfrC strain (76% of the Tet^R^ clones were unable to grow on LB but able to grow on LB supplemented with mevalonate) but not by the P801 and B8 Hfr (none of 80 Tet^R^ clones were unable to grow on LB) indicating that the suppressor mutation was located between the transfer starts of these two later Hfr, that is 3,19 and 7,8 min on the chromosomal map. More precise localizations were obtained by the transduction of the suppressor strains with P1 stocks made on various donor strains carrying Tn*10* insertions [58], selecting Tet^R^ transductant on LB plates supplemented with tetracycline and mevalonate and subsequently scoring the clones on plates devoid of mevalonate. Because we obtained Tet^R^ clones unable to grow in the absence of mevalonate, we concluded that the *supSI* allele was about 30% co-transducible with the *zad-220*::Tn*10* marker (3,2 min). Suppressors *supYI-1*, *-2* and *-3* were located in the same way between the injection points of KL98 and KL14 (54.3 and 68.5 min, respectively), while *supYI-4* was located between the injection points of B7 and PK19 (32 and 43.5 min, respectively). The *supYI-1*, *-2* and *-3* mutations were about 80% co-transducible with *zfh-208*::Tn*10* (57.4 min) and *supYI-4* was 30% co-transducible with *zdi-925*::Tn*10* (38.3 min). Therefore, we PCR-amplified the *erpA* region from the chromosome of the *supSI-1*containing strain using the EcoErpA and XhoErpA oligonucleotides, and its nucleotide sequence analyzed. Similarly, the *iscR* region of *supYI-1*, *-2* and *-3* containing strains and the *sufA* region of the *supYI-4* containing strain were amplified and sequenced. As control, we sequenced PCR amplifications of the regions of the parental strain used to select the suppressors.

### Computational Analyses

The complete sequences of 622 prokaryotic (581 bacterial and 41 archaeal) genomes available in February 2008 were downloaded from the NCBI FTP website (ftp.ncbi.nih.gov). The HMMER package [68] and self-written scripts were then used to search for ATC homologs in these complete genomes, requiring the presence of Fe-S_biosyn domain (Iron-sulphur cluster biosynthesis, PFAM accession number PF01521, PFAM 22.0 version) [69],[70]. Alignments E-value with the Fe-S_biosyn profile less than 0.1 were considered as significant. The corresponding sequences were subsequently analyzed with the same software in order to determine the presence of additional known functional domains. Additional BLASTP/tBLASTN searches [71] were performed in complete genomes to ensure that the ATC family was exhaustively sampled and in the *nr* database at the NCBI to retrieve eukaryotic sequences. For each homolog, the gene context, defined as the 5 neighboring genes located upstream and downstream, was investigated using self-written scripts.

The retrieved homologous sequences were aligned using CLUSTALW 1.83 [72]. The resulting alignment was then visually inspected and manually refined using ED program from the MUST package [73]. Regions where homology between amino acid positions was doubtful were removed from the phylogenomic analyses. The phylogeny of all the prokaryotic ATC was constructed using the maximum likelihood method implemented in the PHYML software [74] with a WAG model (including an estimated proportion of invariant sites). According to the high number of sequences (911) and the small number of sites (76 positions), bootstrap analysis was not performed. An in-depth phylogenomic analysis using a more restricted sequence sampling representative of the diversity of bacterial and eukaryotic *sensu stricto* ATCs was performed using the bayesian approach (58 sequences, 90 positions) implemented in program MrBAYES 3.1.2 (with a mixed substitution model and a gamma law (4 rate categories) and a proportion of invariant sites to take among-site rate variation into account [75]). The Markov chain Monte Carlo search was run with 4 chains for 1,000,000 generations, with trees being sampled every 100 generations (the first 2,500 trees were discarded as “burnin”).

## Supporting Information

Figure S1 Maximum likelihood tree of the A-type protein family. Because of the high number of sequences included (911) and the low number of amino acids positions used for the phylogenetic analysis, the tree is poorly resolved, especially for the most basal nodes. However, a number of monophyletic groups corresponding to ATC subfamilies can be identified. The scale bar represents the average number of substitutions per site.
(0.10 MB PDF)

Figure S2 The *isc* and *suf* mutations have no polar effect on downstream genes. Electrophoresis analysis of the PCR products obtained by amplification of the *hscA* (A) or *sufE* (B) genes using cDNA from various mutants. C) Example of control experiments for complete removal of contaminating DNA from the RNA preparations. Parallel PCR reactions were carried out using reverse reaction (RT) or RNA in a same dilution.
(0.88 MB PPT)

Table S1 Taxonomic distribution of ATC proteins. The taxonomic distribution, the accession number and type of each ATC detected in complete genomes available in February 2008 are indicated. ATC are shown in pink, ATC-I are shown in blue, ATC-II are shown in yellow, ATC-III are shown in green and proteins containing a truncated PF01521 domain and/or a PF01521 domain associated with additional domains are shown in orange. For additional details concerning the definition of ATC types, see text.
(0.05 MB PDF)

Table S2 Sequence (5′ to 3′) of the oligonucleotides used in this work.
(0.05 MB DOC)
