# Supplementary material for: Iron-Sulfur (Fe/S) Protein Biogenesis: Phylogenomic and Genetic Studies of A-Type Carriers
Source: PLoS Genet. 2009 May 29;5(5):e1000497. doi: 10.1371/journal.pgen.1000497 (PMC2682760; doi:10.1371/journal.pgen.1000497)
Supplement: Figure S2 — The isc and suf mutations have no polar effect on downstream genes. Electrophoresis analysis of the PCR products obtained by amplification of the hscA (A) or sufE (B) genes using cDNA from various mutants. C) Example of control experiments for complete removal of contaminating DNA from the RNA preparations. Parallel PCR reactions were carried out using reverse reaction (RT) or RNA in a same dilution. (0.88 MB PPT) [file pgen.1000497.s002.ppt]

## Slide 1
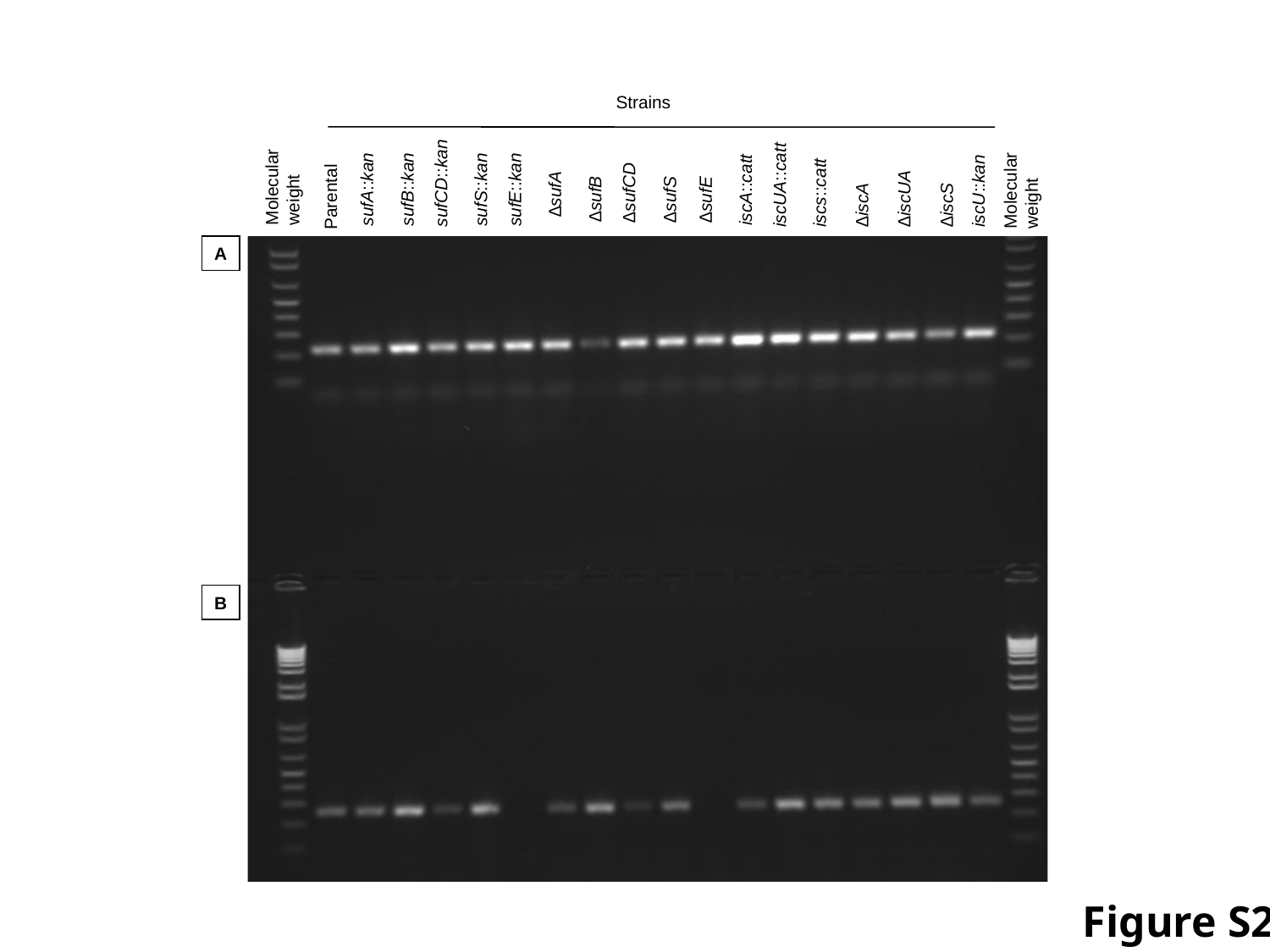

Strains
Parental
Molecular
weight
Molecular
weight
∆sufB
sufCD::kan
iscUA::catt
sufA::kan
sufB::kan
sufS::kan
sufE::kan
iscA::catt
iscU::kan
∆sufCD
iscs::catt
∆sufA
∆iscUA
∆sufS
∆sufE
∆iscA
∆iscS
A
B
Figure S2

## Slide 2
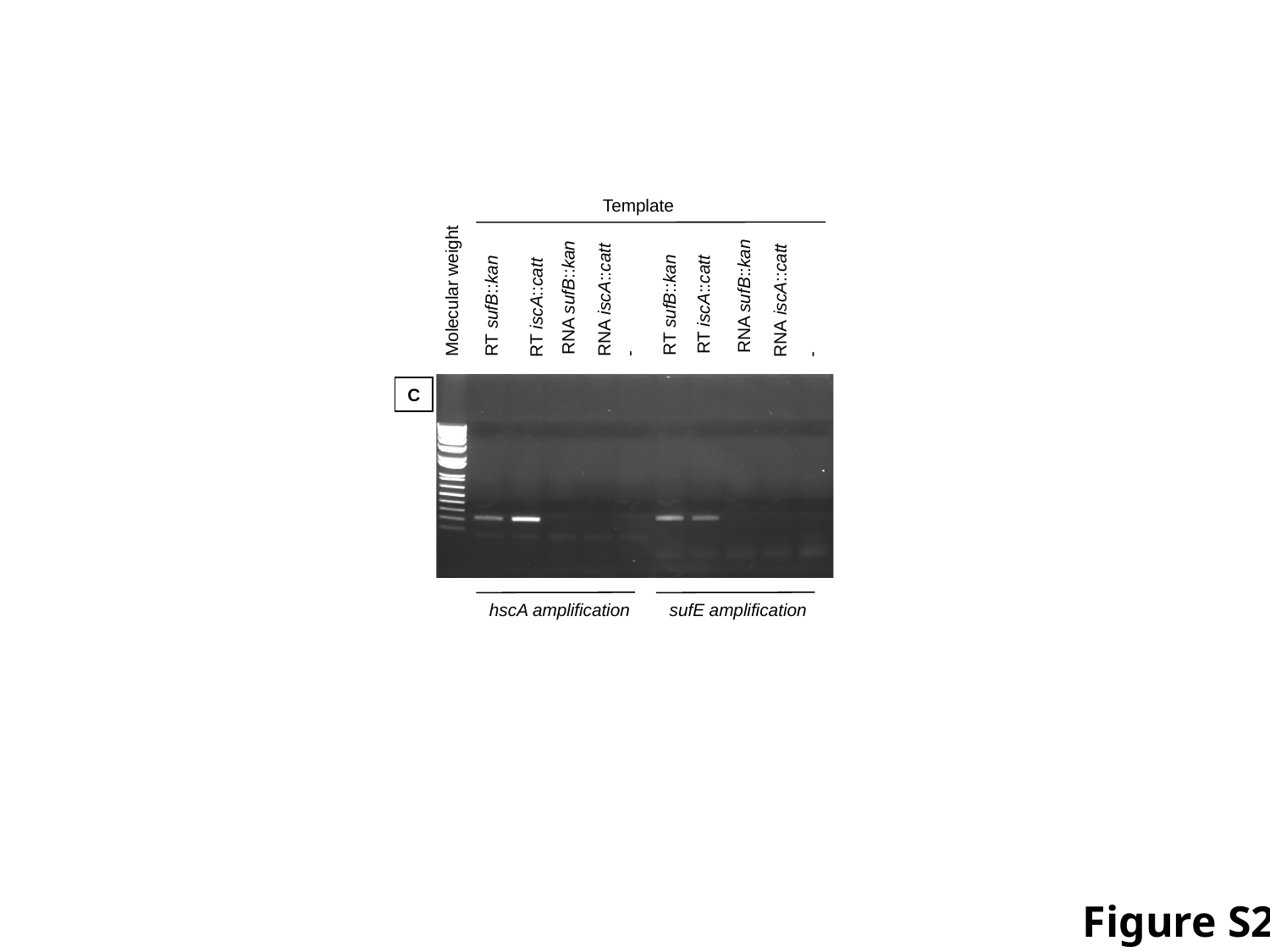

Template
Molecular weight
RNA sufB::kan
RNA sufB::kan
RNA iscA::catt
RNA iscA::catt
RT iscA::catt
RT sufB::kan
RT sufB::kan
RT iscA::catt
-
-
C
hscA amplification
sufE amplification
Figure S2
