# Supplementary material for: Iron-Sulfur (Fe/S) Protein Biogenesis: Phylogenomic and Genetic Studies of A-Type Carriers
Source: PLoS Genet. 2009 May 29;5(5):e1000497. doi: 10.1371/journal.pgen.1000497 (PMC2682760; doi:10.1371/journal.pgen.1000497)
Supplement: Table S1 — Taxonomic distribution of ATC proteins. The taxonomic distribution, the accession number and type of each ATC detected in complete genomes available in February 2008 are indicated. ATC are shown in pink, ATC-I are shown in blue, ATC-II are shown in yellow, ATC-III are shown in green and proteins containing a truncated PF01521 domain and/or a PF01521 domain associated with additional domains are shown in orange. For additional details concerning the definition of ATC types, see text. (0.05 MB PDF) [file pgen.1000497.s003.pdf]

Genome	GENES								CLASSIFICATION				
	ATC		ATC-I subfamily		ATC-II subfamily		ATC-III subfamily	Others	Kingdom	Phylum	class	Order	Family
**Eucarya**													
Unikonts - Fungi													
Ajellomyces capsulatus NAm1			XP_001536972		XP_001544630								
Aspergillus clavatus			XP_001275336		XP_001271941								
Aspergillus fumigatus A1163			XP_755370		XP_751758								
Aspergillus nidulans FGSC A4			XP_663557		XP_659578								
Aspergillus niger			XP_001397771		XP_001401965								
Botryotinia fuckeliana B05.10			XP_001546002		XP_001550237								
Chaetomium globosum CBS 148.51			XP_001221625		XP_001228527								
Coccidioides immitis RS			XP_001244913		XP_001247701								
Gibberella zeae			XP_382719		XP_391063								
Neosartorya fischeri NRRL 181			XP_001260519		XP_001266949								
Neurospora crassa OR74A			XP_962949		XP_963568								
Phaeosphaeria nodorum SN15			EAT84657		EAT77052								
Sclerotinia sclerotiorum 1980			XP_001597918		XP_001584689								
Ashbya gossypii ATCC 10895			NP_983762		NP_986633								
Candida albicans SC5314			XP_719906		XP_719203								
Candida glabrata CBS 138			XP_445480		XP_445845	XP_446532							
Debaryomyces hansenii CBS767			XP_460574		XP_456615								
Kluyveromyces lactis NRRL Y-1140			XP_451952		XP_455894								
Lodderomyces elongisporus NRRL YB-4239			XP_001526456		XP_001524006								
Pichia guilliermondii ATCC 6260			XP_001485649		XP_001486399								
Pichia stipitis CBS 6054			XP_001382658		XP_001384097								
Saccharomyces cerevisiae			NP_015392		NP_013073								
Vanderwaltozyma polyspora DSM 70294			XP_001644863		XP_001643820								
Yarrowia lipolytica CLIB122			XP_504712		XP_504136								
Schizosaccharomyces pombe			NP_596675		NP_588112								
Cryptococcus neoformans var. neoformans JEC21			XP_777984		XP_571058								
Laccaria bicolor S238N-H82			EDR09121		EDR15688								
Malassezia globosa CBS 7966			XP_001729801		XP_001731537								
Ustilago maydis 521			XP_761618		XP_756981								
Encephalitozoon cuniculi GB-M1													
Nosema bombycis													
Unikonts - Metazoa													
Danio rerio			AAI29412		NP_001020349								
Tetraodon nigroviridis			CAG10610		CAG08611								
Nematostella vectensis			XP_001625113		XP_001627048								
Strongylocentrotus purpuratus			XP_783841		XP_798918								
Aedes aegypti			XP_001652137		XP_001656446								
Anopheles gambiae str. PEST			XP_319229		XP_312196								
Apis mellifera			XP_001120329		XP_624993								
Culex pipiens quinquefasciatus			EDS34908		EDS36884								
Drosophila melanogaster			NP_651267		NP_573062								
Nasonia vitripennis			XP_001599351		XP_001608294								
Tribolium castaneum			XP_974074		XP_968263								
Gallus gallus			XP_421267		XP_001231752								
Equus caballus			XP_001490475		XP_001496112								
Homo sapiens			NP_919255		NP_112202	XP_001134052							
Monodelphis domestica			XP_001375248		XP_001362457								
Mus musculus			XP_203592		XP_504136								
Pan troglodytes			XP_001143075		XP_001141878	XP_528342							
Rattus norvegicus			NP_001102748		NP_853657								
Brugia malayi			EDP36039		EDP29041								
Caenorhabditis elegans			NP_496981		NP_741696								
Caenorhabditis briggsae			CAE65928		CAE73141								
Schistosoma japonicum			AAW27011		AAP06468								
Unikonts - Choanoflagella													
Monosiga brevicollis MX1			XP_001743260		XP_001747866								
Unikonts - Conosa													
Dictyostelium discoideum AX4			XP_638418		XP_641284								
Entamoeba dispar SAW760													
Entamoeba histolytica HM-1													
Bikonts - Plantae													
Chlamydomonas reinhardtii	XP_001691442		XP_001692587		XP_001697636								
Ostreococcus lucimarinus CCE9901			XP_001420265		XP_001419388								
Ostreococcus tauri			CAL55696		CAL55019								
Arabidopsis thaliana	AAD39571		AAM64402		AAM64677								
Oryza sativa Indica Group	NP_001056794		EAZ06779		NP_001066782								
Physcomitrella patens subsp. patens	XP_001774897<br>XP_001758765	XP_001756275	XP_001784130	XP_001769262	XP_001758568								
Vitis vinifera	CAO71750		CAO15908		CAO15918								
Guillardia theta	CAH25356		?		CAH25346								

Genome	GENES							CLASSIFICATION				
	ATC	ATC-I subfamily		ATC-II subfamily		ATC-III subfamily	Others	Kingdom	Phylum	class	Order	Family
Bikonts - Alveolata												
Babesia bovis		XP_001611819		XP_001611858								
Cryptosporidium_muris		XP_002142828										
Plasmodium berghei		XP_678370		XP_677114	XP_678755							
Plasmodium chabaudi		XP_740474		XP_734230								
Plasmodium falciparum 3D7		XP_001349582		XP_001351784	XP_001351295							
Plasmodium vivax		XP_001612996		XP_001613731	XP_001614488							
Plasmodium yoelii yoelii		XP_729917		XP_728986	XP_730789							
Theileria annulata		XP_953242		XP_953277								
Theileria parva		XP_764232		XP_764271								
Toxoplasma gondii	EEB04073	EEB02616		AAT57939								
Paramecium tetraurelia		XP_001444331		XP_001436239	XP_001449196							
Tetrahymena thermophila SB210		XP_001030255		XP_001010095								
Bikonts - Stramenopiles												
Phytophthora_infestans	Uncomplete	Uncomplete		Uncomplete								
Phytophthora_ramorum	Uncomplete	Uncomplete		Uncomplete								
Thalassiosira_pseudonana	EED96297	Uncomplete		Uncomplete								
Bikonts - (Excavata) Euglenozoa												
Trypanosoma brucei		XP_844777		XP_847384								
Trypanosoma cruzi		XP_816359		XP_812534								
Leishmania braziliensis		XP_001568236		XP_001563686								
Leishmania infantum		XP_001468947		XP_001464571								
Leishmania major		XP_843241		XP_001682162								
Bikonts - (Excavata) Diplomonadida												
Giardia lamblia ATCC 50803		XP_001707906										
Bikonts - (Excavata) Heterolobosea												
Trichomonas vaginalis G3		XP_001305704<br>XP_001583028	XP_001316269<br>XP_001305699									
**Archaea**												
Crenarchaeota												
Aeropyrum pernix K1								Archaea	Crenarchaeota	Thermoprotei	Desulfurococcales	Desulfurococcaceae
Caldivirga maquilingensis IC-167								Archaea	Crenarchaeota	Thermoprotei	Thermoproteales	Thermoproteaceae
Hyperthermus butylicus DSM 5456								Archaea	Crenarchaeota	Thermoprotei	Desulfurococcales	Pyrodictiaceae
Ignicoccus hospitalis KIN4/I								Archaea	Crenarchaeota	Thermoprotei	Desulfurococcales	Desulfurococcaceae
Metallosphaera sedula DSM 5348								Archaea	Crenarchaeota	Thermoprotei	Sulfolobales	Sulfolobaceae
Pyrobaculum aerophilum str. IM2								Archaea	Crenarchaeota	Thermoprotei	Thermoproteales	Thermoproteaceae
Pyrobaculum arsenaticum DSM 13514								Archaea	Crenarchaeota	Thermoprotei	Thermoproteales	Thermoproteaceae
Pyrobaculum calidifontis JCM 11548								Archaea	Crenarchaeota	Thermoprotei	Thermoproteales	Thermoproteaceae
Pyrobaculum islandicum DSM 4184								Archaea	Crenarchaeota	Thermoprotei	Thermoproteales	Thermoproteaceae
Staphylothermus marinus F1								Archaea	Crenarchaeota	Thermoprotei	Desulfurococcales	Desulfurococcaceae
Sulfolobus acidocaldarius DSM 639								Archaea	Crenarchaeota	Thermoprotei	Sulfolobales	Sulfolobaceae
Sulfolobus solfataricus P2								Archaea	Crenarchaeota	Thermoprotei	Sulfolobales	Sulfolobaceae
Sulfolobus tokodaii str. 7								Archaea	Crenarchaeota	Thermoprotei	Sulfolobales	Sulfolobaceae
Thermofilum pendens Hrk 5								Archaea	Crenarchaeota	Thermoprotei	Thermoproteales	Thermofilaceae
Euryarchaeota												
Archaeoglobus fulgidus DSM 4304								Archaea	Euryarchaeota	Archaeoglobi	Archaeoglobales	Archaeoglobaceae
Candidatus Methanoregula boonei 6A8								Archaea	Euryarchaeota	Methanomicrobia	Methanomicrobiales	NA
Haloarcula marismortui ATCC 43049	YP_135036							Archaea	Euryarchaeota	Halobacteria	Halobacteriales	Halobacteriaceae
Halobacterium sp. NRC-1								Archaea	Euryarchaeota	Halobacteria	Halobacteriales	Halobacteriaceae
Haloquadratum walsbyi DSM 16790	YP_658434							Archaea	Euryarchaeota	Halobacteria	Halobacteriales	Halobacteriaceae
Methanobrevibacter smithii ATCC 35061								Archaea	Euryarchaeota	Methanobacteria	Methanobacteriales	Methanobacteriaceae
Methanocaldococcus jannaschii DSM 2661								Archaea	Euryarchaeota	Methanococci	Methanococcales	Methanocaldococcaceae
Methanococcoides burtonii DSM 6242	YP_566425							Archaea	Euryarchaeota	Methanomicrobia	Methanosarcinales	Methanosarcinaceae
Methanococcus aeolicus Nankai-3								Archaea	Euryarchaeota	Methanococci	Methanococcales	Methanococcaceae
Methanococcus maripaludis C5								Archaea	Euryarchaeota	Methanococci	Methanococcales	Methanococcaceae
Methanococcus maripaludis C6								Archaea	Euryarchaeota	Methanococci	Methanococcales	Methanococcaceae
Methanococcus maripaludis C7								Archaea	Euryarchaeota	Methanococci	Methanococcales	Methanococcaceae
Methanococcus maripaludis S2								Archaea	Euryarchaeota	Methanococci	Methanococcales	Methanococcaceae
Methanococcus vannielii SB								Archaea	Euryarchaeota	Methanococci	Methanococcales	Methanococcaceae
Methanocorpusculum labreanum Z								Archaea	Euryarchaeota	Methanomicrobia	Methanomicrobiales	Methanocorpusculaceae
Methanoculleus marisnigri JR1								Archaea	Euryarchaeota	Methanomicrobia	Methanomicrobiales	Methanomicrobiaceae
Methanopyrus kandleri AV19								Archaea	Euryarchaeota	Methanopyri	Methanopyrales	Methanopyraceae
Methanosaeta thermophila PT								Archaea	Euryarchaeota	Methanomicrobia	Methanosarcinales	Methanosaetaceae
Methanosarcina acetivorans C2A	NP_615857							Archaea	Euryarchaeota	Methanomicrobia	Methanosarcinales	Methanosarcinaceae
Methanosarcina barkeri str. Fusaro	YP_307095							Archaea	Euryarchaeota	Methanomicrobia	Methanosarcinales	Methanosarcinaceae
Methanosarcina mazei Go1	NP_634040							Archaea	Euryarchaeota	Methanomicrobia	Methanosarcinales	Methanosarcinaceae
Methanosphaera stadtmanae DSM 3091								Archaea	Euryarchaeota	Methanobacteria	Methanobacteriales	Methanobacteriaceae
Methanospirillum hungatei JF-1								Archaea	Euryarchaeota	Methanomicrobia	Methanomicrobiales	Methanospirillaceae
Methanothermobacter thermautotrophicus str. Delta H								Archaea	Euryarchaeota	Methanobacteria	Methanobacteriales	Methanobacteriaceae
Natronomonas pharaonis DSM 2160	YP_326936							Archaea	Euryarchaeota	Halobacteria	Halobacteriales	Halobacteriaceae
Picrophilus torridus DSM 9790								Archaea	Euryarchaeota	Thermoplasmata	Thermoplasmatales	Picrophilaceae
Pyrococcus abyssi GE5								Archaea	Euryarchaeota	Thermococci	Thermococcales	Thermococcaceae
Pyrococcus furiosus DSM 3638								Archaea	Euryarchaeota	Thermococci	Thermococcales	Thermococcaceae
Pyrococcus horikoshii OT3								Archaea	Euryarchaeota	Thermococci	Thermococcales	Thermococcaceae

Genome	GENES						CLASSIFICATION				
	ATC		ATC-I subfamily	ATC-II subfamily	ATC-III subfamily	Others	Kingdom	Phylum	class	Order	Family
Thermococcus kodakarensis KOD1							Archaea	Euryarchaeota	Thermococci	Thermococcales	Thermococcaceae
Thermoplasma acidophilum DSM 1728							Archaea	Euryarchaeota	Thermoplasmata	Thermoplasmatales	Thermoplasmataceae
Thermoplasma volcanium GSS1							Archaea	Euryarchaeota	Thermoplasmata	Thermoplasmatales	Thermoplasmataceae
uncultured methanogenic archaeon RC-I	YP_685378						Archaea	Euryarchaeota	NA	NA	NA
Nanoarchaeum equitans Kin4-M							Archaea	Nanoarchaeota	NA	NA	NA
Thaumarchaeota											
Nitrosopumilus maritimus SCM1	YP_001582637						Archaea	Crenarchaeota	Thermoprotei	Nitrosopumilales	Nitrosopumilaceae
**Bacteria**											
Acidobacteria											
Acidobacteria bacterium Ellin345	YP_589561	YP_590657					Bacteria	Acidobacteria	Acidobacteria class	Acidobacteriales	Acidobacteriaceae
Solibacter usitatus Ellin6076	YP_825449						Bacteria	Acidobacteria	Solibacteres	Solibacterales	Solibacteraceae
Actinobacteria											
Acidothermus cellulolyticus 11B	YP_872713	YP_873636					Bacteria	Actinobacteria	Actinobacteria class	Actinomycetales	Acidothermaceae
Arthrobacter aurescens TC1	YP_947957						Bacteria	Actinobacteria	Actinobacteria class	Actinomycetales	Micrococcaceae
Arthrobacter sp. FB24	YP_831698						Bacteria	Actinobacteria	Actinobacteria class	Actinomycetales	Micrococcaceae
Bifidobacterium adolescentis ATCC 15703							Bacteria	Actinobacteria	Actinobacteria class	Bifidobacteriales	Bifidobacteriaceae
Bifidobacterium longum NCC2705							Bacteria	Actinobacteria	Actinobacteria class	Bifidobacteriales	Bifidobacteriaceae
Clavibacter michiganensis subsp. michiganensis NCPPB 382	YP_001222585						Bacteria	Actinobacteria	Actinobacteria class	Actinomycetales	Microbacteriaceae
Corynebacterium diphtheriae NCTC 13129	NP_939973						Bacteria	Actinobacteria	Actinobacteria class	Actinomycetales	Corynebacteriaceae
Corynebacterium efficiens YS-314	NP_738699						Bacteria	Actinobacteria	Actinobacteria class	Actinomycetales	Corynebacteriaceae
Corynebacterium glutamicum ATCC 13032	YP_226439						Bacteria	Actinobacteria	Actinobacteria class	Actinomycetales	Corynebacteriaceae
Corynebacterium glutamicum ATCC 13032	NP_601401						Bacteria	Actinobacteria	Actinobacteria class	Actinomycetales	Corynebacteriaceae
Corynebacterium glutamicum R	YP_001138980						Bacteria	Actinobacteria	Actinobacteria class	Actinomycetales	Corynebacteriaceae
Corynebacterium jeikeium K411	YP_250493						Bacteria	Actinobacteria	Actinobacteria class	Actinomycetales	Corynebacteriaceae
Frankia alni ACN14a	YP_715305				YP_716928		Bacteria	Actinobacteria	Actinobacteria class	Actinomycetales	Frankiaceae
Frankia sp. CcI3	YP_482207				YP_483552		Bacteria	Actinobacteria	Actinobacteria class	Actinomycetales	Frankiaceae
Frankia sp. EAN1pec	YP_001506141				YP_001511110		Bacteria	Actinobacteria	Actinobacteria class	Actinomycetales	Frankiaceae
Kineococcus radiotolerans SRS30216	YP_001362989						Bacteria	Actinobacteria	Actinobacteria class	Actinomycetales	Kineosporiaceae
Leifsonia xyli subsp. xyli str. CTCB07	YP_062419						Bacteria	Actinobacteria	Actinobacteria class	Actinomycetales	Microbacteriaceae
Mycobacterium avium 104	YP_881491						Bacteria	Actinobacteria	Actinobacteria class	Actinomycetales	Mycobacteriaceae
Mycobacterium avium subsp. paratuberculosis K-10	NP_960878						Bacteria	Actinobacteria	Actinobacteria class	Actinomycetales	Mycobacteriaceae
Mycobacterium bovis AF2122/97	NP_855876						Bacteria	Actinobacteria	Actinobacteria class	Actinomycetales	Mycobacteriaceae
Mycobacterium bovis BCG str. Pasteur 1173P2	YP_978309						Bacteria	Actinobacteria	Actinobacteria class	Actinomycetales	Mycobacteriaceae
Mycobacterium gilvum PYR-GCK	YP_001134210						Bacteria	Actinobacteria	Actinobacteria class	Actinomycetales	Mycobacteriaceae
Mycobacterium leprae TN	NP_301657						Bacteria	Actinobacteria	Actinobacteria class	Actinomycetales	Mycobacteriaceae
Mycobacterium smegmatis str. MC2 155	YP_888549						Bacteria	Actinobacteria	Actinobacteria class	Actinomycetales	Mycobacteriaceae
Mycobacterium sp. JLS	YP_001071582						Bacteria	Actinobacteria	Actinobacteria class	Actinomycetales	Mycobacteriaceae
Mycobacterium sp. KMS	YP_939348						Bacteria	Actinobacteria	Actinobacteria class	Actinomycetales	Mycobacteriaceae
Mycobacterium sp. MCS	YP_640465						Bacteria	Actinobacteria	Actinobacteria class	Actinomycetales	Mycobacteriaceae
Mycobacterium tuberculosis CDC1551	NP_336732						Bacteria	Actinobacteria	Actinobacteria class	Actinomycetales	Mycobacteriaceae
Mycobacterium tuberculosis F11	YP_001288165						Bacteria	Actinobacteria	Actinobacteria class	Actinomycetales	Mycobacteriaceae
Mycobacterium tuberculosis H37Ra	YP_001283544						Bacteria	Actinobacteria	Actinobacteria class	Actinomycetales	Mycobacteriaceae
Mycobacterium tuberculosis H37Rv	NP_216720						Bacteria	Actinobacteria	Actinobacteria class	Actinomycetales	Mycobacteriaceae
Mycobacterium ulcerans Agy99	YP_907178						Bacteria	Actinobacteria	Actinobacteria class	Actinomycetales	Mycobacteriaceae
Mycobacterium vanbaalenii PYR-1	YP_954368						Bacteria	Actinobacteria	Actinobacteria class	Actinomycetales	Mycobacteriaceae
Nocardia farcinica IFM 10152	YP_117915						Bacteria	Actinobacteria	Actinobacteria class	Actinomycetales	Nocardiaceae
Nocardioides sp. JS614	YP_924335						Bacteria	Actinobacteria	Actinobacteria class	Actinomycetales	Nocardioidaceae
Propionibacterium acnes KPA171202	YP_055415						Bacteria	Actinobacteria	Actinobacteria class	Actinomycetales	Propionibacteriaceae
Renibacterium salmoninarum ATCC 33209	YP_001624735						Bacteria	Actinobacteria	Actinobacteria class	Actinomycetales	Micrococcaceae
Rhodococcus sp. RHA1	YP_701125						Bacteria	Actinobacteria	Actinobacteria class	Actinomycetales	Nocardiaceae
Rubrobacter xylanophilus DSM 9941	YP_645498						Bacteria	Actinobacteria	Actinobacteria class	Rubrobacterales	Rubrobacteraceae
Saccharopolyspora erythraea NRRL 2338	YP_001103898						Bacteria	Actinobacteria	Actinobacteria class	Actinomycetales	Pseudonocardiaceae
Salinispora arenicola CNS-205	YP_001538329	YP_001535882					Bacteria	Actinobacteria	Actinobacteria class	Actinomycetales	Micromonosporaceae
Salinispora tropica CNB-440	YP_001160116	YP_001157940					Bacteria	Actinobacteria	Actinobacteria class	Actinomycetales	Micromonosporaceae
Streptomyces avermitilis MA-4680	NP_827218						Bacteria	Actinobacteria	Actinobacteria class	Actinomycetales	Streptomycetaceae
Streptomyces coelicolor A3(2)	NP_626417						Bacteria	Actinobacteria	Actinobacteria class	Actinomycetales	Streptomycetaceae
Thermobifida fusca YX	YP_289072						Bacteria	Actinobacteria	Actinobacteria class	Actinomycetales	Nocardiopsaceae
Tropheryma whipplei TW08/27	NP_789457						Bacteria	Actinobacteria	Actinobacteria class	Actinomycetales	Cellulomonadaceae
Tropheryma whipplei str. Twist	NP_787370						Bacteria	Actinobacteria	Actinobacteria class	Actinomycetales	Cellulomonadaceae
Aquificae											
Aquifex aeolicus VF5	NP_214277						Bacteria	Aquificae	Aquificae class	Aquificales	Aquificaceae
Bacteroidetes/Chlorobi											
Bacteroides fragilis NCTC 9343							Bacteria	Bacteroidetes	Bacteroidia	Bacteroidales	Bacteroidaceae
Bacteroides fragilis YCH46							Bacteria	Bacteroidetes	Bacteroidia	Bacteroidales	Bacteroidaceae
Bacteroides thetaiotaomicron VPI-5482							Bacteria	Bacteroidetes	Bacteroidia	Bacteroidales	Bacteroidaceae
Bacteroides vulgatus ATCC 8482							Bacteria	Bacteroidetes	Bacteroidia	Bacteroidales	Bacteroidaceae
Candidatus Sulcia muelleri GWSS							Bacteria	Bacteroidetes	Flavobacteria	Flavobacteriales	NA
Cytophaga hutchinsonii ATCC 33406	YP_679118	YP_677389					Bacteria	Bacteroidetes	Sphingobacteria	Sphingobacteriales	Flexibacteraceae
Flavobacterium johnsoniae UW101	YP_001193536						Bacteria	Bacteroidetes	Flavobacteria	Flavobacteriales	Flavobacteriaceae
Flavobacterium psychrophilum JIP02/86	YP_001297250						Bacteria	Bacteroidetes	Flavobacteria	Flavobacteriales	Flavobacteriaceae
Gramella forsetii KT0803	YP_863147						Bacteria	Bacteroidetes	Flavobacteria	Flavobacteriales	Flavobacteriaceae
Parabacteroides distasonis ATCC 8503							Bacteria	Bacteroidetes	Bacteroidia	Bacteroidales	Porphyromonadaceae
Porphyromonas gingivalis W83							Bacteria	Bacteroidetes	Bacteroidia	Bacteroidales	Porphyromonadaceae

Genome	GENES								CLASSIFICATION				
	ATC		ATC-I subfamily	ATC-II subfamily	ATC-III subfamily		Others		Kingdom	Phylum	class	Order	Family
Salinibacter ruber DSM 13855	YP_445512	YP_445442							Bacteria	Bacteroidetes	Sphingobacteria	Sphingobacteriales	NA
Chlorobium chlorochromatii CaD3									Bacteria	Chlorobi	Chlorobia	Chlorobiales	Chlorobiaceae
Chlorobium phaeobacteroides DSM 266									Bacteria	Chlorobi	Chlorobia	Chlorobiales	Chlorobiaceae
Chlorobium tepidum TLS									Bacteria	Chlorobi	Chlorobia	Chlorobiales	Chlorobiaceae
Pelodictyon luteolum DSM 273									Bacteria	Chlorobi	Chlorobia	Chlorobiales	Chlorobiaceae
Prosthecochloris vibrioformis DSM 265									Bacteria	Chlorobi	Chlorobia	Chlorobiales	Chlorobiaceae
Chloroflexi													
Chloroflexus aurantiacus J-10-fl	YP_001633672								Bacteria	Chloroflexi	Chloroflexi class	Chloroflexales	Chloroflexaceae
Dehalococcoides ethenogenes 195									Bacteria	Chloroflexi	Dehalococcoidetes	NA	NA
Dehalococcoides sp. BAV1									Bacteria	Chloroflexi	Dehalococcoidetes	NA	NA
Dehalococcoides sp. CBDB1									Bacteria	Chloroflexi	Dehalococcoidetes	NA	NA
Herpetosiphon aurantiacus ATCC 23779	YP_001545460								Bacteria	Chloroflexi	Chloroflexi class	Herpetosiphonales	Herpetosiphonaceae
Roseiflexus castenholzii DSM 13941	YP_001431868								Bacteria	Chloroflexi	Chloroflexi class	Chloroflexales	Chloroflexaceae
Roseiflexus sp. RS-1	YP_001276442								Bacteria	Chloroflexi	Chloroflexi class	Chloroflexales	Chloroflexaceae
Cyanobacteria													
Acaryochloris marina MBIC11017	YP_001517485	YP_001520006							Bacteria	Cyanobacteria	NA	NA	NA
Anabaena variabilis ATCC 29413	YP_320724	YP_321810			YP_324751	YP_324439			Bacteria	Cyanobacteria	NA	Nostocales	Nostocaceae
Gloeobacter violaceus PCC 7421	NP_927328	NP_925051							Bacteria	Cyanobacteria	Gloeobacteria	Gloeobacterales	NA
Nostoc sp. PCC 7120	NP_486425	NP_488381			NP_485474				Bacteria	Cyanobacteria	NA	Nostocales	Nostocaceae
Prochlorococcus marinus str. AS9601	YP_001008529								Bacteria	Cyanobacteria	NA	Prochlorales	Prochlorococcaceae
Prochlorococcus marinus str. MIT 9211	YP_001550017								Bacteria	Cyanobacteria	NA	Prochlorales	Prochlorococcaceae
Prochlorococcus marinus str. MIT 9215	YP_001483339								Bacteria	Cyanobacteria	NA	Prochlorales	Prochlorococcaceae
Prochlorococcus marinus str. MIT 9301	YP_001090357								Bacteria	Cyanobacteria	NA	Prochlorales	Prochlorococcaceae
Prochlorococcus marinus str. MIT 9303	YP_001018617								Bacteria	Cyanobacteria	NA	Prochlorales	Prochlorococcaceae
Prochlorococcus marinus str. MIT 9312	YP_396617								Bacteria	Cyanobacteria	NA	Prochlorales	Prochlorococcaceae
Prochlorococcus marinus str. MIT 9313	NP_895794								Bacteria	Cyanobacteria	NA	Prochlorales	Prochlorococcaceae
Prochlorococcus marinus str. MIT 9515	YP_001010446								Bacteria	Cyanobacteria	NA	Prochlorales	Prochlorococcaceae
Prochlorococcus marinus str. NATL1A	YP_001014019								Bacteria	Cyanobacteria	NA	Prochlorales	Prochlorococcaceae
Prochlorococcus marinus str. NATL2A	YP_292676								Bacteria	Cyanobacteria	NA	Prochlorales	Prochlorococcaceae
Prochlorococcus marinus subsp. marinus str. CCMP1375	NP_874531								Bacteria	Cyanobacteria	NA	Prochlorales	Prochlorococcaceae
Prochlorococcus marinus subsp. pastoris str. CCMP1986	NP_892237								Bacteria	Cyanobacteria	NA	Prochlorales	Prochlorococcaceae
Synechococcus elongatus PCC 6301	YP_171343								Bacteria	Cyanobacteria	NA	Chroococcales	NA
Synechococcus elongatus PCC 7942	YP_399926								Bacteria	Cyanobacteria	NA	Chroococcales	NA
Synechococcus sp. CC9311	YP_731758								Bacteria	Cyanobacteria	NA	Chroococcales	NA
Synechococcus sp. CC9605	YP_382644								Bacteria	Cyanobacteria	NA	Chroococcales	NA
Synechococcus sp. CC9902	YP_376350								Bacteria	Cyanobacteria	NA	Chroococcales	NA
Synechococcus sp. JA-2-3B'a(2-13)	YP_477862								Bacteria	Cyanobacteria	NA	Chroococcales	NA
Synechococcus sp. JA-3-3Ab	YP_475074								Bacteria	Cyanobacteria	NA	Chroococcales	NA
Synechococcus sp. RCC307	YP_001226530								Bacteria	Cyanobacteria	NA	Chroococcales	NA
Synechococcus sp. WH 7803	YP_001225948								Bacteria	Cyanobacteria	NA	Chroococcales	NA
Synechococcus sp. WH 8102	NP_898305								Bacteria	Cyanobacteria	NA	Chroococcales	NA
Synechocystis sp. PCC 6803	NP_440066	NP_442892							Bacteria	Cyanobacteria	NA	Chroococcales	NA
Thermosynechococcus elongatus BP-1	NP_681657	NP_681254							Bacteria	Cyanobacteria	NA	Chroococcales	NA
Trichodesmium erythraeum IMS101	YP_723452	YP_720401			YP_723625				Bacteria	Cyanobacteria	NA	Oscillatoriales	NA
Deinococcus/Thermus													
Deinococcus geothermalis DSM 11300	YP_603795								Bacteria	Deinococcus-Thermus	Deinococci	Deinococcales	Deinococcaceae
Deinococcus radiodurans R1	NP_294162								Bacteria	Deinococcus-Thermus	Deinococci	Deinococcales	Deinococcaceae
Thermus thermophilus HB27	YP_005241								Bacteria	Deinococcus-Thermus	Deinococci	Thermales	Thermaceae
Thermus thermophilus HB8	YP_144901								Bacteria	Deinococcus-Thermus	Deinococci	Thermales	Thermaceae
Firmicutes													
Alkaliphilus metalliredigens QYMF									Bacteria	Firmicutes	Clostridia	Clostridiales	Clostridiaceae
Alkaliphilus oremlandii OhILAs									Bacteria	Firmicutes	Clostridia	Clostridiales	Clostridiaceae
Aster yellows witches'-broom phytoplasma AYWB									Bacteria	Firmicutes	Mollicutes	Acholeplasmatales	Acholeplasmataceae
Bacillus amyloliquefaciens FZB42	YP_001422489						YP_001421381		Bacteria	Firmicutes	Bacilli	Bacillales	Bacillaceae
Bacillus anthracis str. 'Ames Ancestor'	YP_021824						YP_018991	YP_020300	Bacteria	Firmicutes	Bacilli	Bacillales	Bacillaceae
Bacillus anthracis str. Ames	NP_847353						NP_844730	NP_845929	Bacteria	Firmicutes	Bacilli	Bacillales	Bacillaceae
Bacillus anthracis str. Sterne	YP_031048						YP_028448	YP_029656	Bacteria	Firmicutes	Bacilli	Bacillales	Bacillaceae
Bacillus cereus ATCC 10987	NP_982162	NP_981366					NP_978683	NP_979922	Bacteria	Firmicutes	Bacilli	Bacillales	Bacillaceae
Bacillus cereus ATCC 14579	NP_834607						NP_832047	NP_833337	Bacteria	Firmicutes	Bacilli	Bacillales	Bacillaceae
Bacillus cereus E33L	YP_084897	YP_086239					YP_083701		Bacteria	Firmicutes	Bacilli	Bacillales	Bacillaceae
Bacillus cereus subsp. cytotoxis NVH 391-98	YP_001376743						YP_001375498		Bacteria	Firmicutes	Bacilli	Bacillales	Bacillaceae
Bacillus clausii KSM-K16	YP_176427						YP_175492	YP_175637	Bacteria	Firmicutes	Bacilli	Bacillales	Bacillaceae
Bacillus halodurans C-125	NP_244277						NP_243016	NP_243118	Bacteria	Firmicutes	Bacilli	Bacillales	Bacillaceae
Bacillus licheniformis ATCC 14580	YP_080497						YP_079222		Bacteria	Firmicutes	Bacilli	Bacillales	Bacillaceae
Bacillus licheniformis ATCC 14580	YP_092920						YP_091639		Bacteria	Firmicutes	Bacilli	Bacillales	Bacillaceae
Bacillus pumilus SAFR-032	YP_001488091						YP_001486944		Bacteria	Firmicutes	Bacilli	Bacillales	Bacillaceae
Bacillus subtilis subsp. subtilis str. 168	NP_391096						NP_389688		Bacteria	Firmicutes	Bacilli	Bacillales	Bacillaceae
Bacillus thuringiensis serovar konkukian str. 97-27	YP_038957						YP_036451	YP_037684	Bacteria	Firmicutes	Bacilli	Bacillales	Bacillaceae
Bacillus thuringiensis str. Al Hakam	YP_897176						YP_894905	YP_895999	Bacteria	Firmicutes	Bacilli	Bacillales	Bacillaceae
Caldicellulosiruptor saccharolyticus DSM 8903									Bacteria	Firmicutes	Clostridia	Thermoanaerobacterales	Thermoanaerobacterales Family III. Incertae Sedis
Carboxydothermus hydrogenoformans Z-2901									Bacteria	Firmicutes	Clostridia	Thermoanaerobacterales	Thermoanaerobacteraceae
Clostridium acetobutylicum ATCC 824									Bacteria	Firmicutes	Clostridia	Clostridiales	Clostridiaceae
Clostridium beijerinckii NCIMB 8052									Bacteria	Firmicutes	Clostridia	Clostridiales	Clostridiaceae

Genome	GENES					CLASSIFICATION				
	ATC	ATC-I subfamily	ATC-II subfamily	ATC-III subfamily	Others	Kingdom	Phylum	class	Order	Family
Clostridium botulinum A str. ATCC 19397						Bacteria	Firmicutes	Clostridia	Clostridiales	Clostridiaceae
Clostridium botulinum A str. ATCC 3502						Bacteria	Firmicutes	Clostridia	Clostridiales	Clostridiaceae
Clostridium botulinum A str. Hall						Bacteria	Firmicutes	Clostridia	Clostridiales	Clostridiaceae
Clostridium botulinum F str. Langeland						Bacteria	Firmicutes	Clostridia	Clostridiales	Clostridiaceae
Clostridium difficile 630						Bacteria	Firmicutes	Clostridia	Clostridiales	Clostridiaceae
Clostridium kluyveri DSM 555						Bacteria	Firmicutes	Clostridia	Clostridiales	Clostridiaceae
Clostridium novyi NT						Bacteria	Firmicutes	Clostridia	Clostridiales	Clostridiaceae
Clostridium perfringens ATCC 13124	YP_694596					Bacteria	Firmicutes	Clostridia	Clostridiales	Clostridiaceae
Clostridium perfringens SM101	YP_697468					Bacteria	Firmicutes	Clostridia	Clostridiales	Clostridiaceae
Clostridium perfringens str. 13						Bacteria	Firmicutes	Clostridia	Clostridiales	Clostridiaceae
Clostridium phytofermentans ISDg						Bacteria	Firmicutes	Clostridia	Clostridiales	Clostridiaceae
Clostridium tetani E88						Bacteria	Firmicutes	Clostridia	Clostridiales	Clostridiaceae
Clostridium thermocellum ATCC 27405						Bacteria	Firmicutes	Clostridia	Clostridiales	Clostridiaceae
Desulfitobacterium hafniense Y51	YP_518284					Bacteria	Firmicutes	Clostridia	Clostridiales	Peptococcaceae
Desulfotomaculum reducens MI-1						Bacteria	Firmicutes	Clostridia	Clostridiales	Peptococcaceae
Enterococcus faecalis V583					NP_816527	Bacteria	Firmicutes	Bacilli	Lactobacillales	Enterococcaceae
Geobacillus kaustophilus HTA426	YP_148809				YP_147418	Bacteria	Firmicutes	Bacilli	Bacillales	Bacillaceae
Geobacillus thermodenitrificans NG80-2	YP_001126996				YP_001125528	Bacteria	Firmicutes	Bacilli	Bacillales	Bacillaceae
Lactobacillus acidophilus NCFM						Bacteria	Firmicutes	Bacilli	Lactobacillales	Lactobacillaceae
Lactobacillus brevis ATCC 367					YP_795168	Bacteria	Firmicutes	Bacilli	Lactobacillales	Lactobacillaceae
Lactobacillus casei ATCC 334					YP_806879	Bacteria	Firmicutes	Bacilli	Lactobacillales	Lactobacillaceae
Lactobacillus delbrueckii subsp. bulgaricus ATCC 11842						Bacteria	Firmicutes	Bacilli	Lactobacillales	Lactobacillaceae
Lactobacillus delbrueckii subsp. bulgaricus ATCC BAA-365						Bacteria	Firmicutes	Bacilli	Lactobacillales	Lactobacillaceae
Lactobacillus gasseri ATCC 33323						Bacteria	Firmicutes	Bacilli	Lactobacillales	Lactobacillaceae
Lactobacillus helveticus DPC 4571						Bacteria	Firmicutes	Bacilli	Lactobacillales	Lactobacillaceae
Lactobacillus johnsonii NCC 533						Bacteria	Firmicutes	Bacilli	Lactobacillales	Lactobacillaceae
Lactobacillus plantarum WCFS1					NP_785164	Bacteria	Firmicutes	Bacilli	Lactobacillales	Lactobacillaceae
Lactobacillus reuteri F275					YP_001271807	Bacteria	Firmicutes	Bacilli	Lactobacillales	Lactobacillaceae
Lactobacillus sakei subsp. sakei 23K					YP_395979	Bacteria	Firmicutes	Bacilli	Lactobacillales	Lactobacillaceae
Lactobacillus salivarius UCC118						Bacteria	Firmicutes	Bacilli	Lactobacillales	Lactobacillaceae
Lactococcus lactis subsp. cremoris MG1363						Bacteria	Firmicutes	Bacilli	Lactobacillales	Streptococcaceae
Lactococcus lactis subsp. cremoris SK11						Bacteria	Firmicutes	Bacilli	Lactobacillales	Streptococcaceae
Lactococcus lactis subsp. lactis Il1403						Bacteria	Firmicutes	Bacilli	Lactobacillales	Streptococcaceae
Leuconostoc mesenteroides subsp. mesenteroides ATCC 8293						Bacteria	Firmicutes	Bacilli	Lactobacillales	Leuconostocaceae
Listeria innocua Clip11262					NP_470280	Bacteria	Firmicutes	Bacilli	Bacillales	Listeriaceae
Listeria monocytogenes EGD-e					NP_464469	Bacteria	Firmicutes	Bacilli	Bacillales	Listeriaceae
Listeria monocytogenes str. 4b F2365					YP_013565	Bacteria	Firmicutes	Bacilli	Bacillales	Listeriaceae
Listeria welshimeri serovar 6b str. SLCC5334					YP_849121	Bacteria	Firmicutes	Bacilli	Bacillales	Listeriaceae
Mesoplasma florum L1						Bacteria	Firmicutes	Mollicutes	Entomoplasmatales	Entomoplasmataceae
Moorella thermoacetica ATCC 39073						Bacteria	Firmicutes	Clostridia	Thermoanaerobacterales	Thermoanaerobacteraceae
Mycoplasma agalactiae PG2						Bacteria	Firmicutes	Mollicutes	Mycoplasmatales	Mycoplasmataceae
Mycoplasma capricolum subsp. capricolum ATCC 27343						Bacteria	Firmicutes	Mollicutes	Mycoplasmatales	Mycoplasmataceae
Mycoplasma gallisepticum R						Bacteria	Firmicutes	Mollicutes	Mycoplasmatales	Mycoplasmataceae
Mycoplasma genitalium G37						Bacteria	Firmicutes	Mollicutes	Mycoplasmatales	Mycoplasmataceae
Mycoplasma hyopneumoniae 232						Bacteria	Firmicutes	Mollicutes	Mycoplasmatales	Mycoplasmataceae
Mycoplasma hyopneumoniae 7448						Bacteria	Firmicutes	Mollicutes	Mycoplasmatales	Mycoplasmataceae
Mycoplasma hyopneumoniae J						Bacteria	Firmicutes	Mollicutes	Mycoplasmatales	Mycoplasmataceae
Mycoplasma mobile 163K						Bacteria	Firmicutes	Mollicutes	Mycoplasmatales	Mycoplasmataceae
Mycoplasma mycoides subsp. mycoides SC str. PG1						Bacteria	Firmicutes	Mollicutes	Mycoplasmatales	Mycoplasmataceae
Mycoplasma penetrans HF-2						Bacteria	Firmicutes	Mollicutes	Mycoplasmatales	Mycoplasmataceae
Mycoplasma pneumoniae M129						Bacteria	Firmicutes	Mollicutes	Mycoplasmatales	Mycoplasmataceae
Mycoplasma pulmonis UAB CTIP						Bacteria	Firmicutes	Mollicutes	Mycoplasmatales	Mycoplasmataceae
Mycoplasma synoviae 53						Bacteria	Firmicutes	Mollicutes	Mycoplasmatales	Mycoplasmataceae
Oceanobacillus iheyensis HTE831	NP_693274				NP_692607	Bacteria	Firmicutes	Bacilli	Bacillales	Bacillaceae
Oenococcus oeni PSU-1						Bacteria	Firmicutes	Bacilli	Lactobacillales	Leuconostocaceae
Onion yellows phytoplasma OY-M						Bacteria	Firmicutes	Mollicutes	Acholeplasmatales	Acholeplasmataceae
Pediococcus pentosaceus ATCC 25745						Bacteria	Firmicutes	Bacilli	Lactobacillales	Lactobacillaceae
Pelotomaculum thermopropionicum SI						Bacteria	Firmicutes	Clostridia	Clostridiales	Peptococcaceae
Staphylococcus aureus RF122	YP_416296				YP_416686	Bacteria	Firmicutes	Bacilli	Bacillales	Staphylococcaceae
Staphylococcus aureus subsp. aureus COL	YP_185812				YP_186240	Bacteria	Firmicutes	Bacilli	Bacillales	Staphylococcaceae
Staphylococcus aureus subsp. aureus JH1	YP_001316102				YP_001316578	Bacteria	Firmicutes	Bacilli	Bacillales	Staphylococcaceae
Staphylococcus aureus subsp. aureus JH9	YP_001246317				YP_001246784	Bacteria	Firmicutes	Bacilli	Bacillales	Staphylococcaceae
Staphylococcus aureus subsp. aureus MRSA252	YP_040324				YP_040769	Bacteria	Firmicutes	Bacilli	Bacillales	Staphylococcaceae
Staphylococcus aureus subsp. aureus MSSA476	YP_042937				YP_043416	Bacteria	Firmicutes	Bacilli	Bacillales	Staphylococcaceae
Staphylococcus aureus subsp. aureus MW2	NP_645639				NP_646056	Bacteria	Firmicutes	Bacilli	Bacillales	Staphylococcaceae
Staphylococcus aureus subsp. aureus Mu3	YP_001441525				YP_001441930	Bacteria	Firmicutes	Bacilli	Bacillales	Staphylococcaceae
Staphylococcus aureus subsp. aureus Mu50	NP_371464				NP_371876	Bacteria	Firmicutes	Bacilli	Bacillales	Staphylococcaceae
Staphylococcus aureus subsp. aureus N315	NP_374062				NP_374465	Bacteria	Firmicutes	Bacilli	Bacillales	Staphylococcaceae
Staphylococcus aureus subsp. aureus NCTC 8325	YP_499430				YP_499877	Bacteria	Firmicutes	Bacilli	Bacillales	Staphylococcaceae
Staphylococcus aureus subsp. aureus USA300	YP_493543				YP_493945	Bacteria	Firmicutes	Bacilli	Bacillales	Staphylococcaceae
Staphylococcus aureus subsp. aureus USA300_TCH1516	YP_001574801				YP_001575176	Bacteria	Firmicutes	Bacilli	Bacillales	Staphylococcaceae
Staphylococcus aureus subsp. aureus str. Newman					YP_001332299	Bacteria	Firmicutes	Bacilli	Bacillales	Staphylococcaceae
Staphylococcus epidermidis ATCC 12228	NP_764189				NP_764589	Bacteria	Firmicutes	Bacilli	Bacillales	Staphylococcaceae

Genome	GENES					CLASSIFICATION				
	ATC	ATC-I subfamily	ATC-II subfamily	ATC-III subfamily	Others	Kingdom	Phylum	class	Order	Family
Staphylococcus epidermidis RP62A	YP_188118				YP_188502	Bacteria	Firmicutes	Bacilli	Bacillales	Staphylococcaceae
Staphylococcus haemolyticus JCSC1435	YP_253926				YP_253471	Bacteria	Firmicutes	Bacilli	Bacillales	Staphylococcaceae
Staphylococcus saprophyticus subsp. saprophyticus ATCC 15305	YP_301925				YP_301496	Bacteria	Firmicutes	Bacilli	Bacillales	Staphylococcaceae
Streptococcus agalactiae 2603V/R						Bacteria	Firmicutes	Bacilli	Lactobacillales	Streptococcaceae
Streptococcus agalactiae A909						Bacteria	Firmicutes	Bacilli	Lactobacillales	Streptococcaceae
Streptococcus agalactiae NEM316						Bacteria	Firmicutes	Bacilli	Lactobacillales	Streptococcaceae
Streptococcus gordonii str. Challis substr. CH1						Bacteria	Firmicutes	Bacilli	Lactobacillales	Streptococcaceae
Streptococcus mutans UA159						Bacteria	Firmicutes	Bacilli	Lactobacillales	Streptococcaceae
Streptococcus pneumoniae D39						Bacteria	Firmicutes	Bacilli	Lactobacillales	Streptococcaceae
Streptococcus pneumoniae R6						Bacteria	Firmicutes	Bacilli	Lactobacillales	Streptococcaceae
Streptococcus pneumoniae TIGR4						Bacteria	Firmicutes	Bacilli	Lactobacillales	Streptococcaceae
Streptococcus pyogenes M1 GAS						Bacteria	Firmicutes	Bacilli	Lactobacillales	Streptococcaceae
Streptococcus pyogenes MGAS10270						Bacteria	Firmicutes	Bacilli	Lactobacillales	Streptococcaceae
Streptococcus pyogenes MGAS10394						Bacteria	Firmicutes	Bacilli	Lactobacillales	Streptococcaceae
Streptococcus pyogenes MGAS10750						Bacteria	Firmicutes	Bacilli	Lactobacillales	Streptococcaceae
Streptococcus pyogenes MGAS2096						Bacteria	Firmicutes	Bacilli	Lactobacillales	Streptococcaceae
Streptococcus pyogenes MGAS315						Bacteria	Firmicutes	Bacilli	Lactobacillales	Streptococcaceae
Streptococcus pyogenes MGAS5005						Bacteria	Firmicutes	Bacilli	Lactobacillales	Streptococcaceae
Streptococcus pyogenes MGAS6180						Bacteria	Firmicutes	Bacilli	Lactobacillales	Streptococcaceae
Streptococcus pyogenes MGAS8232						Bacteria	Firmicutes	Bacilli	Lactobacillales	Streptococcaceae
Streptococcus pyogenes MGAS9429						Bacteria	Firmicutes	Bacilli	Lactobacillales	Streptococcaceae
Streptococcus pyogenes SSI-1						Bacteria	Firmicutes	Bacilli	Lactobacillales	Streptococcaceae
Streptococcus pyogenes str. Manfredo						Bacteria	Firmicutes	Bacilli	Lactobacillales	Streptococcaceae
Streptococcus sanguinis SK36						Bacteria	Firmicutes	Bacilli	Lactobacillales	Streptococcaceae
Streptococcus suis 05ZYH33						Bacteria	Firmicutes	Bacilli	Lactobacillales	Streptococcaceae
Streptococcus suis 98HAH33						Bacteria	Firmicutes	Bacilli	Lactobacillales	Streptococcaceae
Streptococcus thermophilus CNRZ1066						Bacteria	Firmicutes	Bacilli	Lactobacillales	Streptococcaceae
Streptococcus thermophilus LMD-9						Bacteria	Firmicutes	Bacilli	Lactobacillales	Streptococcaceae
Streptococcus thermophilus LMG 18311						Bacteria	Firmicutes	Bacilli	Lactobacillales	Streptococcaceae
Symbiobacterium thermophilum IAM 14863	YP_074812					Bacteria	Firmicutes	Clostridia	Clostridiales	Clostridiales Family XVIII. Incertae Sedis
Syntrophomonas wolfei subsp. wolfei str. Goettingen						Bacteria	Firmicutes	Clostridia	Clostridiales	Syntrophomonadaceae
Thermoanaerobacter tengcongensis MB4						Bacteria	Firmicutes	Clostridia	Thermoanaerobacterales	Thermoanaerobacteraceae
Ureaplasma parvum serovar 3 str. ATCC 700970						Bacteria	Firmicutes	Mollicutes	Mycoplasmatales	Mycoplasmataceae
Fusobacteria										
Fusobacterium nucleatum subsp. nucleatum ATCC 25586						Bacteria	Fusobacteria	Fusobacteria class	Fusobacteriales	Fusobacteriaceae
PVC										
Candidatus Protochlamydia amoebophila UWE25		YP_008084				Bacteria	PVC_Chlamydiae	Chlamydiae class	Chlamydiales	Parachlamydiaceae
Chlamydia muridarum Nigg						Bacteria	PVC_Chlamydiae	Chlamydiae class	Chlamydiales	Chlamydiaceae
Chlamydia trachomatis A/HAR-13						Bacteria	PVC_Chlamydiae	Chlamydiae class	Chlamydiales	Chlamydiaceae
Chlamydia trachomatis D/UW-3/CX						Bacteria	PVC_Chlamydiae	Chlamydiae class	Chlamydiales	Chlamydiaceae
Chlamydophila abortus S26/3						Bacteria	PVC_Chlamydiae	Chlamydiae class	Chlamydiales	Chlamydiaceae
Chlamydophila caviae GPIC						Bacteria	PVC_Chlamydiae	Chlamydiae class	Chlamydiales	Chlamydiaceae
Chlamydophila felis Fe/C-56						Bacteria	PVC_Chlamydiae	Chlamydiae class	Chlamydiales	Chlamydiaceae
Chlamydophila pneumoniae AR39						Bacteria	PVC_Chlamydiae	Chlamydiae class	Chlamydiales	Chlamydiaceae
Chlamydophila pneumoniae CWL029						Bacteria	PVC_Chlamydiae	Chlamydiae class	Chlamydiales	Chlamydiaceae
Chlamydophila pneumoniae J138						Bacteria	PVC_Chlamydiae	Chlamydiae class	Chlamydiales	Chlamydiaceae
Chlamydophila pneumoniae TW-183						Bacteria	PVC_Chlamydiae	Chlamydiae class	Chlamydiales	Chlamydiaceae
Rhodopirellula baltica SH 1	NP_866308 NP_866203					Bacteria	PVC_Planctomycetes	Planctomycetacia	Planctomycetales	Planctomycetaceae
Alphaproteobacteria										
Acidiphilium cryptum JF-5			YP_001235380			Bacteria	Proteobacteria	Alphaproteobacteria	Rhodospirillales	Acetobacteraceae
Agrobacterium tumefaciens str. C58		NP_354701	NP_354803			Bacteria	Proteobacteria	Alphaproteobacteria	Rhizobiales	Rhizobiaceae
Anaplasma marginale str. St. Maries		YP_153930				Bacteria	Proteobacteria	Alphaproteobacteria	Rickettsiales	Anaplasmataceae
Anaplasma phagocytophilum HZ		YP_505380	YP_505258			Bacteria	Proteobacteria	Alphaproteobacteria	Rickettsiales	Anaplasmataceae
Azorhizobium caulinodans ORS 571		YP_001525724	YP_001526525	YP_001526325 YP_001526332		Bacteria	Proteobacteria	Alphaproteobacteria	Rhizobiales	Xanthobacteraceae
Bartonella bacilliformis KC583		YP_989048	YP_988921			Bacteria	Proteobacteria	Alphaproteobacteria	Rhizobiales	Bartonellaceae
Bartonella henselae str. Houston-1		YP_033782	YP_033552			Bacteria	Proteobacteria	Alphaproteobacteria	Rhizobiales	Bartonellaceae
Bartonella quintana str. Toulouse		YP_032396	YP_032188			Bacteria	Proteobacteria	Alphaproteobacteria	Rhizobiales	Bartonellaceae
Bartonella tribocorum CIP 105476		YP_001609727	YP_001609691			Bacteria	Proteobacteria	Alphaproteobacteria	Rhizobiales	Bartonellaceae
Bradyrhizobium japonicum USDA 110		NP_771399	NP_770984	NP_768395		Bacteria	Proteobacteria	Alphaproteobacteria	Rhizobiales	Bradyrhizobiaceae
Bradyrhizobium sp. BTAi1		YP_001240369	YP_001239947	YP_001241757 YP_001241750		Bacteria	Proteobacteria	Alphaproteobacteria	Rhizobiales	Bradyrhizobiaceae
Bradyrhizobium sp. ORS278		YP_001206032	YP_001205552	YP_001207319 YP_001207312		Bacteria	Proteobacteria	Alphaproteobacteria	Rhizobiales	Bradyrhizobiaceae
Brucella abortus biovar 1 str. 9-941		YP_221613	YP_221673			Bacteria	Proteobacteria	Alphaproteobacteria	Rhizobiales	Brucellaceae
Brucella canis ATCC 23365		YP_001592722	YP_001592782			Bacteria	Proteobacteria	Alphaproteobacteria	Rhizobiales	Brucellaceae
Brucella melitensis 16M		NP_540008	NP_539951			Bacteria	Proteobacteria	Alphaproteobacteria	Rhizobiales	Brucellaceae
Brucella melitensis biovar Abortus 2308		YP_414319	YP_414381			Bacteria	Proteobacteria	Alphaproteobacteria	Rhizobiales	Brucellaceae
Brucella ovis ATCC 25840		YP_001258849	YP_001258912			Bacteria	Proteobacteria	Alphaproteobacteria	Rhizobiales	Brucellaceae
Brucella suis 1330		NP_697888	NP_697950			Bacteria	Proteobacteria	Alphaproteobacteria	Rhizobiales	Brucellaceae
Brucella suis ATCC 23445		YP_001627550	YP_001627612			Bacteria	Proteobacteria	Alphaproteobacteria	Rhizobiales	Brucellaceae
Candidatus Pelagibacter ubique HTCC1062		YP_266380	YP_266167			Bacteria	Proteobacteria	Alphaproteobacteria	Rickettsiales	NA
Caulobacter crescentus CB15		NP_420816	NP_420665			Bacteria	Proteobacteria	Alphaproteobacteria	Caulobacterales	Caulobacteraceae
Dinoroseobacter shibae DFL 12		YP_001533073	YP_001533686			Bacteria	Proteobacteria	Alphaproteobacteria	Rhodobacterales	Rhodobacteraceae
Ehrlichia canis str. Jake		YP_303004	YP_303049			Bacteria	Proteobacteria	Alphaproteobacteria	Rickettsiales	Anaplasmataceae
Ehrlichia chaffeensis str. Arkansas		YP_507494	YP_507441			Bacteria	Proteobacteria	Alphaproteobacteria	Rickettsiales	Anaplasmataceae

Genome	GENES						CLASSIFICATION				
	ATC	ATC-I subfamily	ATC-II subfamily	ATC-III subfamily		Others	Kingdom	Phylum	class	Order	Family
Ehrlichia ruminantium str. Gardel		YP_196307	YP_196353				Bacteria	Proteobacteria	Alphaproteobacteria	Rickettsiales	Anaplasmataceae
Ehrlichia ruminantium str. Welgevonden		YP_180238	YP_180282				Bacteria	Proteobacteria	Alphaproteobacteria	Rickettsiales	Anaplasmataceae
Ehrlichia ruminantium str. Welgevonden		YP_197261	YP_197309				Bacteria	Proteobacteria	Alphaproteobacteria	Rickettsiales	Anaplasmataceae
Erythrobacter litoralis HTCC2594		YP_459478	YP_459437				Bacteria	Proteobacteria	Alphaproteobacteria	Sphingomonadales	Erythrobacteraceae
Gluconacetobacter diazotrophicus PAl 5		YP_001602472	YP_001603285	YP_001600730			Bacteria	Proteobacteria	Alphaproteobacteria	Rhodospirillales	Acetobacteraceae
Gluconobacter oxydans 621H		YP_192146					Bacteria	Proteobacteria	Alphaproteobacteria	Rhodospirillales	Acetobacteraceae
Granulibacter bethesdensis CGDNIH1		YP_745083	YP_745140				Bacteria	Proteobacteria	Alphaproteobacteria	Rhodospirillales	Acetobacteraceae
Hyphomonas neptunium ATCC 15444		YP_760749	YP_761301				Bacteria	Proteobacteria	Alphaproteobacteria	Rhodobacterales	Hyphomonadaceae
Jannaschia sp. CCS1		YP_509446	YP_510467				Bacteria	Proteobacteria	Alphaproteobacteria	Rhodobacterales	Rhodobacteraceae
Magnetospirillum magneticum AMB-1		YP_421894	YP_422389	YP_420926			Bacteria	Proteobacteria	Alphaproteobacteria	Rhodospirillales	Rhodospirillaceae
Maricaulis maris MCS10		YP_757145	YP_756551				Bacteria	Proteobacteria	Alphaproteobacteria	Rhodobacterales	Hyphomonadaceae
Mesorhizobium loti MAFF303099		NP_102759	NP_101914	NP_106517			Bacteria	Proteobacteria	Alphaproteobacteria	Rhizobiales	Phyllobacteriaceae
Mesorhizobium sp. BNC1		YP_674374	YP_674335				Bacteria	Proteobacteria	Alphaproteobacteria	Rhizobiales	Phyllobacteriaceae
Methylobacterium extorquens PA1		YP_001640849	YP_001641424				Bacteria	Proteobacteria	Alphaproteobacteria	Rhizobiales	Methylobacteriaceae
Neorickettsia sennetsu str. Miyayama			YP_506191				Bacteria	Proteobacteria	Alphaproteobacteria	Rickettsiales	Anaplasmataceae
Nitrobacter hamburgensis X14		YP_577030	YP_577575				Bacteria	Proteobacteria	Alphaproteobacteria	Rhizobiales	Bradyrhizobiaceae
Nitrobacter winogradskyi Nb-255		YP_318422	YP_318272				Bacteria	Proteobacteria	Alphaproteobacteria	Rhizobiales	Bradyrhizobiaceae
Novosphingobium aromaticivorans DSM 12444		YP_495438	YP_495484				Bacteria	Proteobacteria	Alphaproteobacteria	Sphingomonadales	Sphingomonadaceae
Ochrobactrum anthropi ATCC 49188		YP_001370896	YP_001370792				Bacteria	Proteobacteria	Alphaproteobacteria	Rhizobiales	Brucellaceae
Orientia tsutsugamushi Boryong		YP_001248423	YP_001248705				Bacteria	Proteobacteria	Alphaproteobacteria	Rickettsiales	Rickettsiaceae
Paracoccus denitrificans PD1222		YP_914735	YP_918064				Bacteria	Proteobacteria	Alphaproteobacteria	Rhodobacterales	Rhodobacteraceae
Parvibaculum lavamentivorans DS-1		YP_001414284	YP_001414519				Bacteria	Proteobacteria	Alphaproteobacteria	Rhizobiales	Phyllobacteriaceae
Rhizobium etli CFN 42		YP_469336	YP_469754	NP_659818			Bacteria	Proteobacteria	Alphaproteobacteria	Rhizobiales	Rhizobiaceae
Rhizobium leguminosarum bv. viciae 3841		YP_767637	YP_768160				Bacteria	Proteobacteria	Alphaproteobacteria	Rhizobiales	Rhizobiaceae
Rhodobacter sphaeroides 2.4.1		YP_352714	YP_352000	YP_353605			Bacteria	Proteobacteria	Alphaproteobacteria	Rhodobacterales	Rhodobacteraceae
Rhodobacter sphaeroides ATCC 17025		YP_001167402	YP_001166555	YP_001167461			Bacteria	Proteobacteria	Alphaproteobacteria	Rhodobacterales	Rhodobacteraceae
Rhodobacter sphaeroides ATCC 17029		YP_001043198	YP_001042487	YP_001044059			Bacteria	Proteobacteria	Alphaproteobacteria	Rhodobacterales	Rhodobacteraceae
Rhodopseudomonas palustris BisA53		YP_781546	YP_781879	YP_783439	YP_783423		Bacteria	Proteobacteria	Alphaproteobacteria	Rhizobiales	Bradyrhizobiaceae
Rhodopseudomonas palustris BisB18		YP_532376	YP_532702	YP_534312	YP_534295		Bacteria	Proteobacteria	Alphaproteobacteria	Rhizobiales	Bradyrhizobiaceae
Rhodopseudomonas palustris BisB5		YP_569926	YP_569592	YP_568204	YP_568222		Bacteria	Proteobacteria	Alphaproteobacteria	Rhizobiales	Bradyrhizobiaceae
Rhodopseudomonas palustris CGA009		NP_948197	NP_947812	NP_949962	NP_949944		Bacteria	Proteobacteria	Alphaproteobacteria	Rhizobiales	Bradyrhizobiaceae
Rhodopseudomonas palustris HaA2		YP_486365	YP_486601	YP_484582	YP_484600		Bacteria	Proteobacteria	Alphaproteobacteria	Rhizobiales	Bradyrhizobiaceae
Rhodospirillum rubrum ATCC 11170		YP_426868	YP_427652	YP_427358		YP_425855	Bacteria	Proteobacteria	Alphaproteobacteria	Rhodospirillales	Rhodospirillaceae
Rickettsia akari str. Hartford		YP_001492940	YP_001493571				Bacteria	Proteobacteria	Alphaproteobacteria	Rickettsiales	Rickettsiaceae
Rickettsia bellii OSU 85-389		YP_001495517	YP_001496178				Bacteria	Proteobacteria	Alphaproteobacteria	Rickettsiales	Rickettsiaceae
Rickettsia bellii RML369-C		YP_538469	YP_538115				Bacteria	Proteobacteria	Alphaproteobacteria	Rickettsiales	Rickettsiaceae
Rickettsia canadensis str. McKiel		YP_001491798	YP_001492199				Bacteria	Proteobacteria	Alphaproteobacteria	Rickettsiales	Rickettsiaceae
Rickettsia conorii str. Malish 7		NP_359730	NP_360365				Bacteria	Proteobacteria	Alphaproteobacteria	Rickettsiales	Rickettsiaceae
Rickettsia felis URRWXCal2		YP_246127	YP_246859				Bacteria	Proteobacteria	Alphaproteobacteria	Rickettsiales	Rickettsiaceae
Rickettsia massiliae MTU5		YP_001498953	YP_001499485				Bacteria	Proteobacteria	Alphaproteobacteria	Rickettsiales	Rickettsiaceae
Rickettsia prowazekii str. Madrid E		NP_220457	NP_220861				Bacteria	Proteobacteria	Alphaproteobacteria	Rickettsiales	Rickettsiaceae
Rickettsia rickettsii str. 'Sheila Smith'		YP_001494209	YP_001494845				Bacteria	Proteobacteria	Alphaproteobacteria	Rickettsiales	Rickettsiaceae
Rickettsia typhi str. Wilmington		YP_067037	YP_067426				Bacteria	Proteobacteria	Alphaproteobacteria	Rickettsiales	Rickettsiaceae
Roseobacter denitrificans OCh 114		YP_683361	YP_683166				Bacteria	Proteobacteria	Alphaproteobacteria	Rhodobacterales	Rhodobacteraceae
Silicibacter pomeroyi DSS-3		YP_167721	YP_167830				Bacteria	Proteobacteria	Alphaproteobacteria	Rhodobacterales	Rhodobacteraceae
Silicibacter sp. TM1040		YP_612896	YP_612760				Bacteria	Proteobacteria	Alphaproteobacteria	Rhodobacterales	Rhodobacteraceae
Sinorhizobium medicae WSM419		YP_001326842	YP_001327143				Bacteria	Proteobacteria	Alphaproteobacteria	Rhizobiales	Rhizobiaceae
Sinorhizobium meliloti 1021		NP_385627	NP_385836				Bacteria	Proteobacteria	Alphaproteobacteria	Rhizobiales	Rhizobiaceae
Sphingomonas wittichii RW1		YP_001264395	YP_001263403				Bacteria	Proteobacteria	Alphaproteobacteria	Sphingomonadales	Sphingomonadaceae
Sphingopyxis alaskensis RB2256		YP_617223	YP_615721				Bacteria	Proteobacteria	Alphaproteobacteria	Sphingomonadales	Sphingomonadaceae
Wolbachia endosymbiont of Drosophila melanogaster		NP_966468	NP_966636				Bacteria	Proteobacteria	Alphaproteobacteria	Rickettsiales	Rickettsiaceae
Wolbachia endosymbiont strain TRS of Brugia malayi		YP_197858	YP_198587				Bacteria	Proteobacteria	Alphaproteobacteria	Rickettsiales	Rickettsiaceae
Xanthobacter autotrophicus Py2		YP_001419121	YP_001419491	YP_001415022	YP_001415029	YP_001415065	Bacteria	Proteobacteria	Alphaproteobacteria	Rhizobiales	Xanthobacteraceae
Zymomonas mobilis subsp. mobilis ZM4		YP_163137	YP_162164	YP_163567			Bacteria	Proteobacteria	Alphaproteobacteria	Sphingomonadales	Sphingomonadaceae
Betaproteobacteria											
Acidovorax avenae subsp. citrulli AAC00-1		YP_972364	YP_970794				Bacteria	Proteobacteria	Betaproteobacteria	Burkholderiales	Comamonadaceae
Acidovorax sp. JS42		YP_984951	YP_986387				Bacteria	Proteobacteria	Betaproteobacteria	Burkholderiales	Comamonadaceae
Azoarcus sp. BH72		YP_934261	YP_933518	YP_932058			Bacteria	Proteobacteria	Betaproteobacteria	Rhodocyclales	Rhodocyclaceae
Azoarcus sp. EbN1		YP_157522	YP_160686				Bacteria	Proteobacteria	Betaproteobacteria	Rhodocyclales	Rhodocyclaceae
Bordetella bronchiseptica RB50		NP_890887	NP_888821				Bacteria	Proteobacteria	Betaproteobacteria	Burkholderiales	Alcaligenaceae
Bordetella parapertussis 12822		NP_886032	NP_884288				Bacteria	Proteobacteria	Betaproteobacteria	Burkholderiales	Alcaligenaceae
Bordetella pertussis Tohama I		NP_881537	NP_880507				Bacteria	Proteobacteria	Betaproteobacteria	Burkholderiales	Alcaligenaceae
Bordetella petrii		YP_001629200	YP_001631385				Bacteria	Proteobacteria	Betaproteobacteria	Burkholderiales	Alcaligenaceae
Burkholderia ambifaria AMMD		YP_772468	YP_774051				Bacteria	Proteobacteria	Betaproteobacteria	Burkholderiales	Burkholderiaceae
Burkholderia cenocepacia AU 1054		YP_620085	YP_625791				Bacteria	Proteobacteria	Betaproteobacteria	Burkholderiales	Burkholderiaceae
Burkholderia cenocepacia HI2424		YP_834327	YP_835768				Bacteria	Proteobacteria	Betaproteobacteria	Burkholderiales	Burkholderiaceae
Burkholderia mallei ATCC 23344		YP_103902	YP_103326				Bacteria	Proteobacteria	Betaproteobacteria	Burkholderiales	Burkholderiaceae
Burkholderia mallei NCTC 10229		YP_001027102	YP_001029046				Bacteria	Proteobacteria	Betaproteobacteria	Burkholderiales	Burkholderiaceae
Burkholderia mallei NCTC 10247		YP_001082047	YP_001081034				Bacteria	Proteobacteria	Betaproteobacteria	Burkholderiales	Burkholderiaceae
Burkholderia mallei SAVP1		YP_991606	YP_993527				Bacteria	Proteobacteria	Betaproteobacteria	Burkholderiales	Burkholderiaceae
Burkholderia multivorans ATCC 17616		YP_001580883	YP_001579333				Bacteria	Proteobacteria	Betaproteobacteria	Burkholderiales	Burkholderiaceae
Burkholderia pseudomallei 1106a		YP_001067649	YP_001066906				Bacteria	Proteobacteria	Betaproteobacteria	Burkholderiales	Burkholderiaceae
Burkholderia pseudomallei 1710b		YP_334790	YP_334117				Bacteria	Proteobacteria	Betaproteobacteria	Burkholderiales	Burkholderiaceae

Genome	GENES								CLASSIFICATION				
	ATC		ATC-I subfamily		ATC-II subfamily		ATC-III subfamily	Others	Kingdom	Phylum	class	Order	Family
Burkholderia pseudomallei 668			YP_001060387		YP_001059623				Bacteria	Proteobacteria	Betaproteobacteria	Burkholderiales	Burkholderiaceae
Burkholderia pseudomallei K96243			YP_109503		YP_108883				Bacteria	Proteobacteria	Betaproteobacteria	Burkholderiales	Burkholderiaceae
Burkholderia sp. 383			YP_368012		YP_369668				Bacteria	Proteobacteria	Betaproteobacteria	Burkholderiales	Burkholderiaceae
Burkholderia thailandensis E264			YP_441782		YP_442407				Bacteria	Proteobacteria	Betaproteobacteria	Burkholderiales	Burkholderiaceae
Burkholderia vietnamiensis G4			YP_001118494	YP_001115171	YP_001115170	YP_001120040	YP_001115176		Bacteria	Proteobacteria	Betaproteobacteria	Burkholderiales	Burkholderiaceae
Burkholderia xenovorans LB400			YP_560377	YP_553872	YP_553873	YP_559452	YP_553867		Bacteria	Proteobacteria	Betaproteobacteria	Burkholderiales	Burkholderiaceae
Chromobacterium violaceum ATCC 12472			NP_903364		NP_900762				Bacteria	Proteobacteria	Betaproteobacteria	Neisseriales	Neisseriaceae
Dechloromonas aromatica RCB			YP_283880		YP_285168		YP_284732		Bacteria	Proteobacteria	Betaproteobacteria	Rhodocyclales	Rhodocyclaceae
Delftia acidovorans SPH-1			YP_001566925		YP_001565013				Bacteria	Proteobacteria	Betaproteobacteria	Burkholderiales	Comamonadaceae
Herminiimonas arsenicoxydans			YP_001098603		YP_001100493				Bacteria	Proteobacteria	Betaproteobacteria	Burkholderiales	Oxalobacteraceae
Janthinobacterium sp. Marseille			YP_001351989		YP_001352944				Bacteria	Proteobacteria	Betaproteobacteria	Burkholderiales	Oxalobacteraceae
Methylibium petroleiphilum PM1			YP_001022341		YP_001021451				Bacteria	Proteobacteria	Betaproteobacteria	Burkholderiales	NA
Methylobacillus flagellatus KT			YP_544172		YP_544915				Bacteria	Proteobacteria	Betaproteobacteria	Methylophilales	Methylophilaceae
Neisseria gonorrhoeae FA 1090			YP_208481		YP_207772				Bacteria	Proteobacteria	Betaproteobacteria	Neisseriales	Neisseriaceae
Neisseria meningitidis 053442			YP_001598657		YP_001599425				Bacteria	Proteobacteria	Betaproteobacteria	Neisseriales	Neisseriaceae
Neisseria meningitidis FAM18			YP_974597		YP_975332				Bacteria	Proteobacteria	Betaproteobacteria	Neisseriales	Neisseriaceae
Neisseria meningitidis MC58			NP_273601		NP_274397				Bacteria	Proteobacteria	Betaproteobacteria	Neisseriales	Neisseriaceae
Neisseria meningitidis Z2491			NP_283537		NP_284312				Bacteria	Proteobacteria	Betaproteobacteria	Neisseriales	Neisseriaceae
Nitrosomonas europaea ATCC 19718			NP_841469		NP_841492				Bacteria	Proteobacteria	Betaproteobacteria	Nitrosomonadales	Nitrosomonadaceae
Nitrosomonas eutropha C91			YP_747751		YP_747460				Bacteria	Proteobacteria	Betaproteobacteria	Nitrosomonadales	Nitrosomonadaceae
Nitrosospira multiformis ATCC 25196			YP_411256		YP_411394				Bacteria	Proteobacteria	Betaproteobacteria	Nitrosomonadales	Nitrosomonadaceae
Polaromonas naphthalenivorans CJ2			YP_981802	YP_980744	YP_981801	YP_982517	YP_981811		Bacteria	Proteobacteria	Betaproteobacteria	Burkholderiales	Comamonadaceae
Polaromonas sp. JS666			YP_550900		YP_549005				Bacteria	Proteobacteria	Betaproteobacteria	Burkholderiales	Comamonadaceae
Polynucleobacter sp. QLW-P1DMWA-1			YP_001156659		YP_001156269				Bacteria	Proteobacteria	Betaproteobacteria	Burkholderiales	Burkholderiaceae
Ralstonia eutropha H16			YP_725003		YP_725669				Bacteria	Proteobacteria	Betaproteobacteria	Burkholderiales	Burkholderiaceae
Ralstonia eutropha JMP134			YP_294697		YP_295282				Bacteria	Proteobacteria	Betaproteobacteria	Burkholderiales	Burkholderiaceae
Ralstonia metallidurans CH34			YP_582569		YP_583182				Bacteria	Proteobacteria	Betaproteobacteria	Burkholderiales	Burkholderiaceae
Ralstonia solanacearum GMI1000			NP_518615		NP_519142				Bacteria	Proteobacteria	Betaproteobacteria	Burkholderiales	Burkholderiaceae
Rhodoferax ferrireducens T118			YP_522006		YP_523116	YP_523434			Bacteria	Proteobacteria	Betaproteobacteria	Burkholderiales	Comamonadaceae
Thiobacillus denitrificans ATCC 25259			YP_314214		YP_314924			YP_316230	Bacteria	Proteobacteria	Betaproteobacteria	Hydrogenophilales	Hydrogenophilaceae
Verminephrobacter eiseniae EF01-2			YP_997403		YP_997139				Bacteria	Proteobacteria	Betaproteobacteria	Burkholderiales	Comamonadaceae
Deltaproteobacteria													
Anaeromyxobacter dehalogenans 2CP-C	YP_463807	YP_466681							Bacteria	Proteobacteria	Deltaproteobacteria	Myxococcales	Myxococcaceae
Anaeromyxobacter sp. Fw109-5	YP_001377836	YP_001380754							Bacteria	Proteobacteria	Deltaproteobacteria	Myxococcales	Myxococcaceae
Bdellovibrio bacteriovorus HD100	NP_968109								Bacteria	Proteobacteria	Deltaproteobacteria	Bdellovibrionales	Bdellovibrionaceae
Desulfococcus oleovorans Hxd3									Bacteria	Proteobacteria	Deltaproteobacteria	Desulfobacterales	Desulfobacteraceae
Desulfotalea psychrophila LSv54	YP_064724								Bacteria	Proteobacteria	Deltaproteobacteria	Desulfobacterales	Desulfobulbaceae
Desulfovibrio desulfuricans G20	YP_388510								Bacteria	Proteobacteria	Deltaproteobacteria	Desulfovibrionales	Desulfovibrionaceae
Desulfovibrio vulgaris subsp. vulgaris DP4	YP_967132	YP_968064							Bacteria	Proteobacteria	Deltaproteobacteria	Desulfovibrionales	Desulfovibrionaceae
Desulfovibrio vulgaris subsp. vulgaris str. Hildenborough	YP_010601	YP_009583							Bacteria	Proteobacteria	Deltaproteobacteria	Desulfovibrionales	Desulfovibrionaceae
Geobacter metallireducens GS-15									Bacteria	Proteobacteria	Deltaproteobacteria	Desulfuromonadales	Geobacteraceae
Geobacter sulfurreducens PCA	NP_952260								Bacteria	Proteobacteria	Deltaproteobacteria	Desulfuromonadales	Geobacteraceae
Geobacter uraniireducens Rf4									Bacteria	Proteobacteria	Deltaproteobacteria	Desulfuromonadales	Geobacteraceae
Lawsonia intracellularis PHE/MN1-00									Bacteria	Proteobacteria	Deltaproteobacteria	Desulfovibrionales	Desulfovibrionaceae
Myxococcus xanthus DK 1622	YP_633159	YP_630808							Bacteria	Proteobacteria	Deltaproteobacteria	Myxococcales	Myxococcaceae
Pelobacter carbinolicus DSM 2380									Bacteria	Proteobacteria	Deltaproteobacteria	Desulfuromonadales	Pelobacteraceae
Pelobacter propionicus DSM 2379									Bacteria	Proteobacteria	Deltaproteobacteria	Desulfuromonadales	Pelobacteraceae
Sorangium cellulosum 'So ce 56'	YP_001617980	YP_001616513							Bacteria	Proteobacteria	Deltaproteobacteria	Myxococcales	Polyangiaceae
Syntrophobacter fumaroxidans MPOB									Bacteria	Proteobacteria	Deltaproteobacteria	Syntrophobacterales	Syntrophobacteraceae
Syntrophus aciditrophicus SB									Bacteria	Proteobacteria	Deltaproteobacteria	Syntrophobacterales	Syntrophaceae
Epsilonproteobacteria													
Arcobacter butzleri RM4018									Bacteria	Proteobacteria	Epsilonproteobacteria	Campylobacterales	Campylobacteraceae
Campylobacter concisus 13826									Bacteria	Proteobacteria	Epsilonproteobacteria	Campylobacterales	Campylobacteraceae
Campylobacter curvus 525.92									Bacteria	Proteobacteria	Epsilonproteobacteria	Campylobacterales	Campylobacteraceae
Campylobacter fetus subsp. fetus 82-40									Bacteria	Proteobacteria	Epsilonproteobacteria	Campylobacterales	Campylobacteraceae
Campylobacter hominis ATCC BAA-381									Bacteria	Proteobacteria	Epsilonproteobacteria	Campylobacterales	Campylobacteraceae
Campylobacter jejuni RM1221									Bacteria	Proteobacteria	Epsilonproteobacteria	Campylobacterales	Campylobacteraceae
Campylobacter jejuni subsp. doylei 269.97									Bacteria	Proteobacteria	Epsilonproteobacteria	Campylobacterales	Campylobacteraceae
Campylobacter jejuni subsp. jejuni 81-176									Bacteria	Proteobacteria	Epsilonproteobacteria	Campylobacterales	Campylobacteraceae
Campylobacter jejuni subsp. jejuni 81116									Bacteria	Proteobacteria	Epsilonproteobacteria	Campylobacterales	Campylobacteraceae
Campylobacter jejuni subsp. jejuni NCTC 11168									Bacteria	Proteobacteria	Epsilonproteobacteria	Campylobacterales	Campylobacteraceae
Helicobacter acinonychis str. Sheeba									Bacteria	Proteobacteria	Epsilonproteobacteria	Campylobacterales	Helicobacteraceae
Helicobacter hepaticus ATCC 51449									Bacteria	Proteobacteria	Epsilonproteobacteria	Campylobacterales	Helicobacteraceae
Helicobacter pylori 26695									Bacteria	Proteobacteria	Epsilonproteobacteria	Campylobacterales	Helicobacteraceae
Helicobacter pylori HPAG1									Bacteria	Proteobacteria	Epsilonproteobacteria	Campylobacterales	Helicobacteraceae
Helicobacter pylori J99									Bacteria	Proteobacteria	Epsilonproteobacteria	Campylobacterales	Helicobacteraceae
Nitratiruptor sp. SB155-2									Bacteria	Proteobacteria	Epsilonproteobacteria	NA	NA
Sulfurimonas denitrificans DSM 1251									Bacteria	Proteobacteria	Epsilonproteobacteria	Campylobacterales	Helicobacteraceae
Sulfurovum sp. NBC37-1									Bacteria	Proteobacteria	Epsilonproteobacteria	NA	NA
Wolinella succinogenes DSM 1740									Bacteria	Proteobacteria	Epsilonproteobacteria	Campylobacterales	Helicobacteraceae
Gammaproteobacteria													
Acinetobacter baumannii ATCC 17978			YP_001083110		YP_001084659			YP_001084013	Bacteria	Proteobacteria	Gammaproteobacteria	Pseudomonadales	Moraxellaceae
Acinetobacter sp. ADP1			YP_044817		YP_046089			YP_045762	Bacteria	Proteobacteria	Gammaproteobacteria	Pseudomonadales	Moraxellaceae

Genome	ATC	ATC-I subfamily		ATC-II subfamily		ATC-III subfamily	Others		Kingdom	Phylum	class	Order	Family
	GENES								CLASSIFICATION				
Actinobacillus pleuropneumoniae L20		YP_001054151		YP_001053630			YP_001052862		Bacteria	Proteobacteria	Gammaproteobacteria	Pasteurellales	Pasteurellaceae
Actinobacillus succinogenes 130Z		YP_001344331		YP_001344171			YP_001343609		Bacteria	Proteobacteria	Gammaproteobacteria	Pasteurellales	Pasteurellaceae
Aeromonas hydrophila subsp. hydrophila ATCC 7966		YP_857987		YP_856285			YP_854827		Bacteria	Proteobacteria	Gammaproteobacteria	Aeromonadales	Aeromonadaceae
Aeromonas salmonicida subsp. salmonicida A449		YP_001140702		YP_001142382			YP_001143789		Bacteria	Proteobacteria	Gammaproteobacteria	Aeromonadales	Aeromonadaceae
Alcanivorax borkumensis SK2		YP_692082		YP_693589			YP_692910		Bacteria	Proteobacteria	Gammaproteobacteria	Oceanospirillales	Alcanivoracaceae
Alkalilimnicola ehrlichei MLHE-1		YP_741279		YP_742086			YP_743287		Bacteria	Proteobacteria	Gammaproteobacteria	Chromatiales	Ectothiorhodospiraceae
Baumannia cicadellinicola str. Hc (Homalodisca coagulata)		YP_588679		YP_588904			YP_588921		Bacteria	Proteobacteria	Gammaproteobacteria	NA	NA
Buchnera aphidicola str. APS (Acyrthosiphon pisum)		NP_240042		NP_239954			NP_240350		Bacteria	Proteobacteria	Gammaproteobacteria	Enterobacteriales	Enterobacteriaceae
Buchnera aphidicola str. Bp (Baizongia pistaciae)		NP_777820		NP_777745			NP_778086		Bacteria	Proteobacteria	Gammaproteobacteria	Enterobacteriales	Enterobacteriaceae
Buchnera aphidicola str. Cc (Cinara cedri)		YP_802703		YP_802646			YP_802901		Bacteria	Proteobacteria	Gammaproteobacteria	Enterobacteriales	Enterobacteriaceae
Buchnera aphidicola str. Sg (Schizaphis graminum)		NP_660558		NP_660472			NP_660856		Bacteria	Proteobacteria	Gammaproteobacteria	Enterobacteriales	Enterobacteriaceae
Candidatus Blochmannia floridanus		NP_878461		NP_878650			NP_878848		Bacteria	Proteobacteria	Gammaproteobacteria	Enterobacteriales	Enterobacteriaceae
Candidatus Blochmannia pennsylvanicus str. BPEN		YP_277673		YP_277869			YP_278076		Bacteria	Proteobacteria	Gammaproteobacteria	Enterobacteriales	Enterobacteriaceae
Candidatus Carsonella ruddii PV									Bacteria	Proteobacteria	Gammaproteobacteria	NA	NA
Candidatus Ruthia magnifica str. Cm (Calyptogena magnifica)		YP_904237		YP_903797			YP_903650	YP_904045	Bacteria	Proteobacteria	Gammaproteobacteria	NA	NA
Candidatus Vesicomyosocius okutanii HA		YP_001219784		YP_001219372			YP_001219232	YP_001219612	Bacteria	Proteobacteria	Gammaproteobacteria	NA	NA
Chromohalobacter salexigens DSM 3043		YP_575342		YP_574894			YP_573939		Bacteria	Proteobacteria	Gammaproteobacteria	Oceanospirillales	Halomonadaceae
Citrobacter koseri ATCC BAA-895		YP_001454731		YP_001453283	YP_001451853		YP_001456316		Bacteria	Proteobacteria	Gammaproteobacteria	Enterobacteriales	Enterobacteriaceae
Colwellia psychrerythraea 34H		YP_271269		YP_267877			YP_266987		Bacteria	Proteobacteria	Gammaproteobacteria	Alteromonadales	Colwelliaceae
Coxiella burnetii Dugway 5J108-111		YP_001423541		YP_001424790					Bacteria	Proteobacteria	Gammaproteobacteria	Legionellales	Coxiellaceae
Coxiella burnetii RSA 331		YP_001597697		YP_001597197					Bacteria	Proteobacteria	Gammaproteobacteria	Legionellales	Coxiellaceae
Coxiella burnetii RSA 493		NP_820855		NP_820344					Bacteria	Proteobacteria	Gammaproteobacteria	Legionellales	Coxiellaceae
Dichelobacter nodosus VCS1703A									Bacteria	Proteobacteria	Gammaproteobacteria	Cardiobacteriales	Cardiobacteriaceae
Enterobacter sakazakii ATCC BAA-894		YP_001439242		YP_001438180	YP_001436837		YP_001440339		Bacteria	Proteobacteria	Gammaproteobacteria	Enterobacteriales	Enterobacteriaceae
Enterobacter sp. 638		YP_001175434		YP_001176490	YP_001177740		YP_001178533		Bacteria	Proteobacteria	Gammaproteobacteria	Enterobacteriales	Enterobacteriaceae
Erwinia carotovora subsp. atroseptica SCRI1043		YP_051395		YP_049956	YP_051324		YP_052221		Bacteria	Proteobacteria	Gammaproteobacteria	Enterobacteriales	Enterobacteriaceae
Escherichia coli 536		YP_668105		YP_669535	YP_670423		YP_671377		Bacteria	Proteobacteria	Gammaproteobacteria	Enterobacteriales	Enterobacteriaceae
Escherichia coli APEC O1		YP_851356		YP_852776	YP_853669		YP_859002		Bacteria	Proteobacteria	Gammaproteobacteria	Enterobacteriales	Enterobacteriaceae
Escherichia coli CFT073		NP_752141		NP_753974	NP_754935		NP_756055		Bacteria	Proteobacteria	Gammaproteobacteria	Enterobacteriales	Enterobacteriaceae
Escherichia coli E24377A		YP_001461327		YP_001462978	YP_001463851		YP_001464872		Bacteria	Proteobacteria	Gammaproteobacteria	Enterobacteriales	Enterobacteriaceae
Escherichia coli HS		YP_001456941		YP_001458467	YP_001459321		YP_001460209		Bacteria	Proteobacteria	Gammaproteobacteria	Enterobacteriales	Enterobacteriaceae
Escherichia coli K12		NP_414698		NP_416199	NP_417023		NP_417873		Bacteria	Proteobacteria	Gammaproteobacteria	Enterobacteriales	Enterobacteriaceae
Escherichia coli O157:H7 EDL933		NP_285852		NP_288118	NP_289085		NP_289954		Bacteria	Proteobacteria	Gammaproteobacteria	Enterobacteriales	Enterobacteriaceae
Escherichia coli O157:H7 str. Sakai		NP_308187		NP_310418	NP_311421		NP_312283		Bacteria	Proteobacteria	Gammaproteobacteria	Enterobacteriales	Enterobacteriaceae
Escherichia coli UTI89		YP_539213		YP_540883	YP_541841		YP_542875		Bacteria	Proteobacteria	Gammaproteobacteria	Enterobacteriales	Enterobacteriaceae
Escherichia coli W3110		AP_000817		AP_002304	AP_003114		AP_004376		Bacteria	Proteobacteria	Gammaproteobacteria	Enterobacteriales	Enterobacteriaceae
Francisella tularensis subsp. holarctica FTA		YP_001429051		YP_001428838					Bacteria	Proteobacteria	Gammaproteobacteria	Thiotrichales	Francisellaceae
Francisella tularensis subsp. holarctica OSU18		YP_763929		YP_763778					Bacteria	Proteobacteria	Gammaproteobacteria	Thiotrichales	Francisellaceae
Francisella tularensis subsp. holarctica		YP_514182		YP_513998					Bacteria	Proteobacteria	Gammaproteobacteria	Thiotrichales	Francisellaceae
Francisella tularensis subsp. novicida U112		YP_898259		YP_898397					Bacteria	Proteobacteria	Gammaproteobacteria	Thiotrichales	Francisellaceae
Francisella tularensis subsp. tularensis FSC198		YP_666848		YP_666735					Bacteria	Proteobacteria	Gammaproteobacteria	Thiotrichales	Francisellaceae
Francisella tularensis subsp. tularensis SCHU S4		YP_169716		YP_169603					Bacteria	Proteobacteria	Gammaproteobacteria	Thiotrichales	Francisellaceae
Francisella tularensis subsp. tularensis WY96-3418		YP_001122377		YP_001122292					Bacteria	Proteobacteria	Gammaproteobacteria	Thiotrichales	Francisellaceae
Haemophilus ducreyi 35000HP		NP_873214		NP_873561			NP_872959		Bacteria	Proteobacteria	Gammaproteobacteria	Pasteurellales	Pasteurellaceae
Haemophilus influenzae 86-028NP		YP_249433		YP_248091			YP_248145		Bacteria	Proteobacteria	Gammaproteobacteria	Pasteurellales	Pasteurellaceae
Haemophilus influenzae PittEE		YP_001290491		YP_001290094			YP_001290040		Bacteria	Proteobacteria	Gammaproteobacteria	Pasteurellales	Pasteurellaceae
Haemophilus influenzae PittGG		YP_001291894		YP_001292268			YP_001292383		Bacteria	Proteobacteria	Gammaproteobacteria	Pasteurellales	Pasteurellaceae
Haemophilus influenzae Rd KW20		NP_439864		NP_438537			NP_438594		Bacteria	Proteobacteria	Gammaproteobacteria	Pasteurellales	Pasteurellaceae
Haemophilus somnus 129PT		YP_719037		YP_718491			YP_719701		Bacteria	Proteobacteria	Gammaproteobacteria	Pasteurellales	Pasteurellaceae
Hahella chejuensis KCTC 2396		YP_437314		YP_432698			YP_433769		Bacteria	Proteobacteria	Gammaproteobacteria	Oceanospirillales	Hahellaceae
Halorhodospira halophila SL1		YP_001002457		YP_001003359					Bacteria	Proteobacteria	Gammaproteobacteria	Chromatiales	Ectothiorhodospiraceae
Idiomarina loihiensis L2TR		YP_156621		YP_154542			YP_154640		Bacteria	Proteobacteria	Gammaproteobacteria	Alteromonadales	Idiomarinaceae
Klebsiella pneumoniae subsp. pneumoniae MGH 78578		YP_001333861		YP_001335799	YP_001336500		YP_001337438		Bacteria	Proteobacteria	Gammaproteobacteria	Enterobacteriales	Enterobacteriaceae
Legionella pneumophila str. Corby		YP_001250126		YP_001250495					Bacteria	Proteobacteria	Gammaproteobacteria	Legionellales	Legionellaceae
Legionella pneumophila str. Lens		YP_126689		YP_127046					Bacteria	Proteobacteria	Gammaproteobacteria	Legionellales	Legionellaceae
Legionella pneumophila str. Paris		YP_123667		YP_124026					Bacteria	Proteobacteria	Gammaproteobacteria	Legionellales	Legionellaceae
Legionella pneumophila subsp. pneumophila str. Philadelphia 1		YP_095417		YP_095770					Bacteria	Proteobacteria	Gammaproteobacteria	Legionellales	Legionellaceae
Mannheimia succiniciproducens MBEL55E		YP_088499		YP_088915			YP_089424		Bacteria	Proteobacteria	Gammaproteobacteria	Pasteurellales	Pasteurellaceae
Marinobacter aquaeolei VT8		YP_957979		YP_960416			YP_958796		Bacteria	Proteobacteria	Gammaproteobacteria	Alteromonadales	Alteromonadaceae
Marinomonas sp. MWYL1		YP_001339878		YP_001340212			YP_001341201		Bacteria	Proteobacteria	Gammaproteobacteria	Oceanospirillales	Oceanospirillaceae
Methylococcus capsulatus str. Bath		YP_112783	YP_113251	YP_115278	YP_112784	YP_112742			Bacteria	Proteobacteria	Gammaproteobacteria	Methylococcales	Methylococcaceae
Nitrosococcus oceani ATCC 19707		YP_343082		YP_343652					Bacteria	Proteobacteria	Gammaproteobacteria	Chromatiales	Chromatiaceae
Pasteurella multocida subsp. multocida str. Pm70		NP_245395		NP_245257			NP_246496		Bacteria	Proteobacteria	Gammaproteobacteria	Pasteurellales	Pasteurellaceae
Photobacterium profundum SS9		YP_128756		YP_128967			YP_128418		Bacteria	Proteobacteria	Gammaproteobacteria	Vibrionales	Vibrionaceae
Photorhabdus luminescens subsp. laumondii TTO1		NP_928240		NP_929857	NP_930505		NP_927563		Bacteria	Proteobacteria	Gammaproteobacteria	Enterobacteriales	Enterobacteriaceae
Pseudoalteromonas atlantica T6c		YP_660127		YP_660820			YP_663786		Bacteria	Proteobacteria	Gammaproteobacteria	Alteromonadales	Pseudoalteromonadaceae
Pseudoalteromonas haloplanktis TAC125		YP_340749		YP_341159			YP_341338		Bacteria	Proteobacteria	Gammaproteobacteria	Alteromonadales	Pseudoalteromonadaceae
Pseudomonas aeruginosa PA7		YP_001346201		YP_001346685			YP_001348805		Bacteria	Proteobacteria	Gammaproteobacteria	Pseudomonadales	Pseudomonadaceae
Pseudomonas aeruginosa PAO1		NP_249356		NP_252501			NP_250538		Bacteria	Proteobacteria	Gammaproteobacteria	Pseudomonadales	Pseudomonadaceae
Pseudomonas aeruginosa UCBPP-PA14		YP_788835		YP_789320			YP_791394		Bacteria	Proteobacteria	Gammaproteobacteria	Pseudomonadales	Pseudomonadaceae
Pseudomonas entomophila L48		YP_606232		YP_606729			YP_608921		Bacteria	Proteobacteria	Gammaproteobacteria	Pseudomonadales	Pseudomonadaceae
Pseudomonas fluorescens Pf-5		YP_262669		YP_262043			YP_260762		Bacteria	Proteobacteria	Gammaproteobacteria	Pseudomonadales	Pseudomonadaceae
Pseudomonas fluorescens PfO-1		YP_350829		YP_350338			YP_348833		Bacteria	Proteobacteria	Gammaproteobacteria	Pseudomonadales	Pseudomonadaceae

Genome	GENES							CLASSIFICATION				
	ATC	ATC-I subfamily	ATC-II subfamily		ATC-III subfamily	Others		Kingdom	Phylum	class	Order	Family
Pseudomonas mendocina ymp		YP_001189409	YP_001188989			YP_001187653		Bacteria	Proteobacteria	Gammaproteobacteria	Pseudomonadales	Pseudomonadaceae
Pseudomonas putida F1		YP_001265820	YP_001266220			YP_001268627		Bacteria	Proteobacteria	Gammaproteobacteria	Pseudomonadales	Pseudomonadaceae
Pseudomonas putida KT2440		NP_742599	NP_743005			NP_744527		Bacteria	Proteobacteria	Gammaproteobacteria	Pseudomonadales	Pseudomonadaceae
Pseudomonas stutzeri A1501		YP_001171302			YP_001171886	YP_001172722		Bacteria	Proteobacteria	Gammaproteobacteria	Pseudomonadales	Pseudomonadaceae
Pseudomonas syringae pv. phaseolicola 1448A		YP_272983	YP_273569			YP_274816		Bacteria	Proteobacteria	Gammaproteobacteria	Pseudomonadales	Pseudomonadaceae
Pseudomonas syringae pv. syringae B728a		YP_237636	YP_234328			YP_235543		Bacteria	Proteobacteria	Gammaproteobacteria	Pseudomonadales	Pseudomonadaceae
Pseudomonas syringae pv. tomato str. DC3000		NP_790452	NP_791251			NP_792541		Bacteria	Proteobacteria	Gammaproteobacteria	Pseudomonadales	Pseudomonadaceae
Psychrobacter arcticus 273-4		YP_263344	YP_264761			YP_265193		Bacteria	Proteobacteria	Gammaproteobacteria	Pseudomonadales	Moraxellaceae
Psychrobacter cryohalolentis K5		YP_579312	YP_580919			YP_581463		Bacteria	Proteobacteria	Gammaproteobacteria	Pseudomonadales	Moraxellaceae
Psychrobacter sp. PRwf-1		YP_001278957	YP_001280522			YP_001280737		Bacteria	Proteobacteria	Gammaproteobacteria	Pseudomonadales	Moraxellaceae
Psychromonas ingrahamii 37		YP_942308	YP_942750			YP_944488		Bacteria	Proteobacteria	Gammaproteobacteria	Alteromonadales	Psychromonadaceae
Saccharophagus degradans 2-40		YP_526310	YP_526892			YP_527481		Bacteria	Proteobacteria	Gammaproteobacteria	Alteromonadales	Alteromonadaceae
Salmonella enterica subsp. arizonae serovar 62:z4,z23:--		YP_001571790	YP_001570646	YP_001569416		YP_001573034		Bacteria	Proteobacteria	Gammaproteobacteria	Enterobacteriales	Enterobacteriaceae
Salmonella enterica subsp. enterica serovar Choleraesuis str. SC-B67		YP_215191	YP_216376	YP_217522		YP_218430		Bacteria	Proteobacteria	Gammaproteobacteria	Enterobacteriales	Enterobacteriaceae
Salmonella enterica subsp. enterica serovar Paratyphi A str. ATCC 9150		YP_149552	YP_150732	YP_149656		YP_152501		Bacteria	Proteobacteria	Gammaproteobacteria	Enterobacteriales	Enterobacteriaceae
Salmonella enterica subsp. enterica serovar Paratyphi B str. SPB7		YP_001586537	YP_001588182	YP_001586660		YP_001590519		Bacteria	Proteobacteria	Gammaproteobacteria	Enterobacteriales	Enterobacteriaceae
Salmonella enterica subsp. enterica serovar Typhi Ty2		NP_804087	NP_805043	NP_804190		NP_807605		Bacteria	Proteobacteria	Gammaproteobacteria	Enterobacteriales	Enterobacteriaceae
Salmonella enterica subsp. enterica serovar Typhi str. CT18		NP_454812	NP_456155	NP_457072		NP_458393		Bacteria	Proteobacteria	Gammaproteobacteria	Enterobacteriales	Enterobacteriaceae
Salmonella typhimurium LT2		NP_459209	NP_460334	NP_461476		NP_462414		Bacteria	Proteobacteria	Gammaproteobacteria	Enterobacteriales	Enterobacteriaceae
Serratia proteamaculans 568		YP_001477017	YP_001478408	YP_001479849		YP_001480855		Bacteria	Proteobacteria	Gammaproteobacteria	Enterobacteriales	Enterobacteriaceae
Shewanella amazonensis SB2B		YP_926720	YP_927172			YP_929374		Bacteria	Proteobacteria	Gammaproteobacteria	Alteromonadales	Shewanellaceae
Shewanella baltica OS155		YP_001049551	YP_001050757			YP_001048545		Bacteria	Proteobacteria	Gammaproteobacteria	Alteromonadales	Shewanellaceae
Shewanella baltica OS185		YP_001365422	YP_001366585			YP_001368371		Bacteria	Proteobacteria	Gammaproteobacteria	Alteromonadales	Shewanellaceae
Shewanella baltica OS195		YP_001553676	YP_001554928			YP_001556742		Bacteria	Proteobacteria	Gammaproteobacteria	Alteromonadales	Shewanellaceae
Shewanella denitrificans OS217		YP_563817	YP_562468			YP_564526		Bacteria	Proteobacteria	Gammaproteobacteria	Alteromonadales	Shewanellaceae
Shewanella frigidimarina NCIMB 400		YP_751654	YP_751105			YP_748945		Bacteria	Proteobacteria	Gammaproteobacteria	Alteromonadales	Shewanellaceae
Shewanella loihica PV-4		YP_001093147	YP_001094440			YP_001092237		Bacteria	Proteobacteria	Gammaproteobacteria	Alteromonadales	Shewanellaceae
Shewanella oneidensis MR-1		NP_716924	NP_717862			NP_720135		Bacteria	Proteobacteria	Gammaproteobacteria	Alteromonadales	Shewanellaceae
Shewanella pealeana ATCC 700345		YP_001500847	YP_001501350			YP_001503855		Bacteria	Proteobacteria	Gammaproteobacteria	Alteromonadales	Shewanellaceae
Shewanella putrefaciens CN-32		YP_001182650	YP_001183667			YP_001185300		Bacteria	Proteobacteria	Gammaproteobacteria	Alteromonadales	Shewanellaceae
Shewanella sediminis HAW-EB3		YP_001472834	YP_001474604			YP_001471920		Bacteria	Proteobacteria	Gammaproteobacteria	Alteromonadales	Shewanellaceae
Shewanella sp. ANA-3		YP_870697	YP_869913			YP_871626		Bacteria	Proteobacteria	Gammaproteobacteria	Alteromonadales	Shewanellaceae
Shewanella sp. MR-4		YP_735017	YP_733872			YP_735924		Bacteria	Proteobacteria	Gammaproteobacteria	Alteromonadales	Shewanellaceae
Shewanella sp. MR-7		YP_739012	YP_737870			YP_739914		Bacteria	Proteobacteria	Gammaproteobacteria	Alteromonadales	Shewanellaceae
Shewanella sp. W3-18-1		YP_964412	YP_963253			YP_961526		Bacteria	Proteobacteria	Gammaproteobacteria	Alteromonadales	Shewanellaceae
Shigella boydii Sb227		YP_406703	YP_408924	YP_407893		YP_409718		Bacteria	Proteobacteria	Gammaproteobacteria	Enterobacteriales	Enterobacteriaceae
Shigella dysenteriae Sd197		YP_401894	YP_404260			YP_405119		Bacteria	Proteobacteria	Gammaproteobacteria	Enterobacteriales	Enterobacteriaceae
Shigella flexneri 2a str. 2457T		NP_835887	NP_837369	NP_838089		NP_839472		Bacteria	Proteobacteria	Gammaproteobacteria	Enterobacteriales	Enterobacteriaceae
Shigella flexneri 2a str. 301		NP_706104	NP_707583	NP_708367		NP_709191		Bacteria	Proteobacteria	Gammaproteobacteria	Enterobacteriales	Enterobacteriaceae
Shigella flexneri 5 str. 8401		YP_687731	YP_689181	YP_689979		YP_690769		Bacteria	Proteobacteria	Gammaproteobacteria	Enterobacteriales	Enterobacteriaceae
Shigella sonnei Ss046		YP_309196	YP_310410	YP_311473		YP_312336		Bacteria	Proteobacteria	Gammaproteobacteria	Enterobacteriales	Enterobacteriaceae
Sodalis glossinidius str. 'morsitans'		YP_454181	YP_455111			YP_456005		Bacteria	Proteobacteria	Gammaproteobacteria	Enterobacteriales	Enterobacteriaceae
Thiomicrospira crunogena XCL-2		YP_390619	YP_390888					Bacteria	Proteobacteria	Gammaproteobacteria	Thiotrichales	Piscirickettsiaceae
Vibrio cholerae O1 biovar eltor str. N16961		NP_230276	NP_230399			NP_232347		Bacteria	Proteobacteria	Gammaproteobacteria	Vibrionales	Vibrionaceae
Vibrio cholerae O395		YP_001216121	YP_001216235			YP_001218208		Bacteria	Proteobacteria	Gammaproteobacteria	Vibrionales	Vibrionaceae
Vibrio fischeri ES114		YP_205517	YP_204002			YP_205844		Bacteria	Proteobacteria	Gammaproteobacteria	Vibrionales	Vibrionaceae
Vibrio harveyi ATCC BAA-1116		YP_001446588	YP_001444277			YP_001443843		Bacteria	Proteobacteria	Gammaproteobacteria	Vibrionales	Vibrionaceae
Vibrio parahaemolyticus RIMD 2210633		NP_798853	NP_796977			NP_796525		Bacteria	Proteobacteria	Gammaproteobacteria	Vibrionales	Vibrionaceae
Vibrio vulnificus CMCP6		NP_760568	NP_759432			NP_759840		Bacteria	Proteobacteria	Gammaproteobacteria	Vibrionales	Vibrionaceae
Vibrio vulnificus YJ016		NP_935520	NP_933550			NP_933020		Bacteria	Proteobacteria	Gammaproteobacteria	Vibrionales	Vibrionaceae
Wigglesworthia glossinidia endosymbiont of Glossina brevipalpis		NP_871077	NP_871364					Bacteria	Proteobacteria	Gammaproteobacteria	Enterobacteriales	Enterobacteriaceae
Xanthomonas axonopodis pv. citri str. 306		NP_640852	NP_641951			NP_643807	NP_642434	Bacteria	Proteobacteria	Gammaproteobacteria	Xanthomonadales	Xanthomonadaceae
Xanthomonas campestris pv. campestris str. 8004		YP_241601	YP_243741			YP_241891	YP_243179	Bacteria	Proteobacteria	Gammaproteobacteria	Xanthomonadales	Xanthomonadaceae
Xanthomonas campestris pv. campestris str. ATCC 33913		NP_635880	NP_636933			NP_638714	NP_637444	Bacteria	Proteobacteria	Gammaproteobacteria	Xanthomonadales	Xanthomonadaceae
Xanthomonas campestris pv. vesicatoria str. 85-10		YP_362265	YP_363391			YP_365357	YP_364160	Bacteria	Proteobacteria	Gammaproteobacteria	Xanthomonadales	Xanthomonadaceae
Xanthomonas oryzae pv. oryzae KACC10331		YP_199164	YP_201056			YP_199729	YP_201705	Bacteria	Proteobacteria	Gammaproteobacteria	Xanthomonadales	Xanthomonadaceae
Xanthomonas oryzae pv. oryzae MAFF 311018		YP_449517	YP_451324			YP_450017	YP_451948	Bacteria	Proteobacteria	Gammaproteobacteria	Xanthomonadales	Xanthomonadaceae
Xylella fastidiosa 9a5c		NP_297695	NP_299839			NP_299673	NP_299880	Bacteria	Proteobacteria	Gammaproteobacteria	Xanthomonadales	Xanthomonadaceae
Xylella fastidiosa Temecula1		NP_779858	NP_780127			NP_779607	NP_780159	Bacteria	Proteobacteria	Gammaproteobacteria	Xanthomonadales	Xanthomonadaceae
Yersinia enterocolitica subsp. enterocolitica 8081		YP_001005080	YP_001006412	YP_001005391		YP_001008139		Bacteria	Proteobacteria	Gammaproteobacteria	Enterobacteriales	Enterobacteriaceae
Yersinia pestis Angola		YP_001605553	YP_001607003	YP_001605038		YP_001608065		Bacteria	Proteobacteria	Gammaproteobacteria	Enterobacteriales	Enterobacteriaceae
Yersinia pestis Antiqua		YP_652794	YP_651660	YP_652245		YP_653249		Bacteria	Proteobacteria	Gammaproteobacteria	Enterobacteriales	Enterobacteriaceae
Yersinia pestis CO92		NP_406849	NP_405939	NP_406398		NP_403783		Bacteria	Proteobacteria	Gammaproteobacteria	Enterobacteriales	Enterobacteriaceae
Yersinia pestis KIM		NP_668137	NP_669249	NP_668658		NP_671196		Bacteria	Proteobacteria	Gammaproteobacteria	Enterobacteriales	Enterobacteriaceae
Yersinia pestis Nepal516		YP_646635	YP_647788	YP_647173		YP_649865		Bacteria	Proteobacteria	Gammaproteobacteria	Enterobacteriales	Enterobacteriaceae
Yersinia pestis Pestoides F		YP_001164303	YP_001162123	YP_001163587		YP_001161452		Bacteria	Proteobacteria	Gammaproteobacteria	Enterobacteriales	Enterobacteriaceae
Yersinia pestis biovar Microtus str. 91001		NP_991696	NP_993521	NP_993879		NP_991529		Bacteria	Proteobacteria	Gammaproteobacteria	Enterobacteriales	Enterobacteriaceae
Yersinia pseudotuberculosis IP 31758		YP_001402285	YP_001400716	YP_001400151		YP_001402938		Bacteria	Proteobacteria	Gammaproteobacteria	Enterobacteriales	Enterobacteriaceae
Yersinia pseudotuberculosis IP 32953		YP_069285	YP_070829	YP_071364		YP_072254		Bacteria	Proteobacteria	Gammaproteobacteria	Enterobacteriales	Enterobacteriaceae
Unclassified Proteobacteria												
Magnetococcus sp. MC-1		YP_864466	YP_866957			YP_867249		Bacteria	Proteobacteria	NA	NA	NA
Spirochaetes												
Borrelia afzelii PKo								Bacteria	Spirochaetes	Spirochaetes class	Spirochaetales	Spirochaetaceae

Genome	GENES					CLASSIFICATION				
	ATC	ATC-I subfamily	ATC-II subfamily	ATC-III subfamily	Others	Kingdom	Phylum	class	Order	Family
Borrelia burgdorferi B31						Bacteria	Spirochaetes	Spirochaetes class	Spirochaetales	Spirochaetaceae
Borrelia garinii PBi						Bacteria	Spirochaetes	Spirochaetes class	Spirochaetales	Spirochaetaceae
Leptospira borgpetersenii serovar Hardjo-bovis JB197						Bacteria	Spirochaetes	Spirochaetes class	Spirochaetales	Leptospiraceae
Leptospira borgpetersenii serovar Hardjo-bovis L550						Bacteria	Spirochaetes	Spirochaetes class	Spirochaetales	Leptospiraceae
Leptospira interrogans serovar Copenhageni str. Fiocruz L1-130						Bacteria	Spirochaetes	Spirochaetes class	Spirochaetales	Leptospiraceae
Leptospira interrogans serovar Lai str. 56601						Bacteria	Spirochaetes	Spirochaetes class	Spirochaetales	Leptospiraceae
Treponema denticola ATCC 35405						Bacteria	Spirochaetes	Spirochaetes class	Spirochaetales	Spirochaetaceae
Treponema pallidum subsp. pallidum str. Nichols						Bacteria	Spirochaetes	Spirochaetes class	Spirochaetales	Spirochaetaceae
Thermotogae										
Fervidobacterium nodosum Rt17-B1						Bacteria	Thermotogae	Thermotogae class	Thermotogales	Thermotogaceae
Petrotoga mobilis SJ95						Bacteria	Thermotogae	Thermotogae class	Thermotogales	Thermotogaceae
Thermosipho melanesiensis BI429						Bacteria	Thermotogae	Thermotogae class	Thermotogales	Thermotogaceae
Thermotoga lettingae TMO						Bacteria	Thermotogae	Thermotogae class	Thermotogales	Thermotogaceae
Thermotoga maritima MSB8						Bacteria	Thermotogae	Thermotogae class	Thermotogales	Thermotogaceae
Thermotoga petrophila RKU-1						Bacteria	Thermotogae	Thermotogae class	Thermotogales	Thermotogaceae
