# Supplementary material for: Iron-Sulfur (Fe/S) Protein Biogenesis: Phylogenomic and Genetic Studies of A-Type Carriers
Source: PLoS Genet. 2009 May 29;5(5):e1000497. doi: 10.1371/journal.pgen.1000497 (PMC2682760; doi:10.1371/journal.pgen.1000497)
Supplement: Table S2 — Sequence (5′ to 3′) of the oligonucleotides used in this work. (0.05 MB DOC) [file pgen.1000497.s004.doc]

**Table S2.** Sequence (5’ to 3’) of the oligonucleotides used in this work.

| **Primer** | **Sequence** |
| --- | --- |
|  | RT-PCR |
| hscARevRT | CAGTTTTTCTTCGAGTTGTTCGGCACTGC |
| hscAsensRT | ATGGAACAGTTGGAGCTGCGC |
| sufErevRT | GCTAAGTGCAGCGGCTTTGGCGCG |
| sufEsensRT | AGTCAGGTGTGGATTGTCATGCGC |
|  | Cloning |
| EcoIscA | GCATGAATTCATGTCGATTACACTGAGCGAC |
| XhoIscA | GCCTCTCGAGTCAAACGTGGAAGCTTTCGCCG |
| EcoSufA | GCATGAATTCATGAGTGATGACGTAGCACTG |
| XhoSufA | GCCTCTCGAGTTAGATACTAAAGGAAGAACCGCAAC |
| EcoErpA | GCATGAATTCATGGACATGCATTCAGGAACC |
| XhoErpA | GCCTCTCGAGCTATACCCCAAAGCTTTCGCCAC |
| XhoIsa1 | GCCTCTCGAGTTAAACCATGAAACTCTCGCC |
| XhoIsa2 | GCCTCTCGAGTTAATTTTCAATATCAAAACTACTTCC |
| T7 | TAATACGACTCACTATAGGG |
|  | Construction of ∆*iscA*::*cat* and ∆*iscUA*::*cat* deletions |
| iscAUP | aaagcaaacgtgaagcaaaataagagttgaggtttggttcatatgaatatcctcctta |
| iscADO | cacgcgcaggcgaccacggtggggttatcggtatgcgcagtgtaggctggagctgcttc |
| iscU | GCATGAATTCATGGCTTACAGCGAAAAAGT |
| iscA | GCCTTCTCGAGTCAAACGTGGAAGCTTTCGCC |
|  | Construction of the P*_suf_*::*lacZ* gene fusions |
| sufUP | AAAGAATTCGCATGTTTTACGGTAAAGCC |
| sufDO | AAAGGATCCCATGTCTTACTTCACCTCAAAC |
