# Supplementary material for: Iron-Sulfur (Fe/S) Protein Biogenesis: Phylogenomic and Genetic Studies of A-Type Carriers
Source: PLoS Genet. 2009 May 29;5(5):e1000497. doi: 10.1371/journal.pgen.1000497 (PMC2682760; doi:10.1371/journal.pgen.1000497)

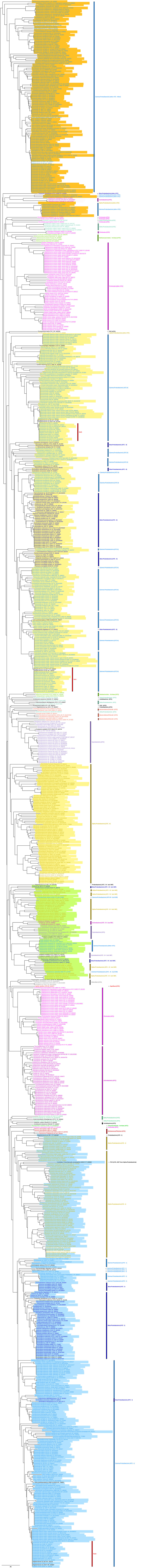

Supplement: Figure S1 — Maximum likelihood tree of the A-type protein family. Because of the high number of sequences included (911) and the low number of amino acids positions used for the phylogenetic analysis, the tree is poorly resolved, especially for the most basal nodes. However, a number of monophyletic groups corresponding to ATC subfamilies can be identified. The scale bar represents the average number of substitutions per site. (0.10 MB PDF) [file pgen.1000497.s001.pdf]
